# Dynamics and durability of HIV-1 neutralization are determined by viral replication

**DOI:** 10.1038/s41591-023-02582-3

**Published:** 2023-11-13

**Authors:** Philipp Schommers, Dae Sung Kim, Maike Schlotz, Christoph Kreer, Ralf Eggeling, Anna Hake, Melanie Stecher, Juyeon Park, Caelan E. Radford, Adam S. Dingens, Meryem S. Ercanoglu, Henning Gruell, Stanley Odidika, Marten Dahlhaus, Lutz Gieselmann, Elvin Ahmadov, Rene Y. Lawong, Eva Heger, Elena Knops, Christoph Wyen, Tim Kümmerle, Katja Römer, Stefan Scholten, Timo Wolf, Christoph Stephan, Isabelle Suárez, Nagarajan Raju, Anurag Adhikari, Stefan Esser, Hendrik Streeck, Ralf Duerr, Aubin J. Nanfack, Susan Zolla-Pazner, Christof Geldmacher, Otto Geisenberger, Arne Kroidl, Wiston William, Lucas Maganga, Nyanda Elias Ntinginya, Ivelin S. Georgiev, Jörg J. Vehreschild, Michael Hoelscher, Gerd Fätkenheuer, Jason J. Lavinder, Jesse D. Bloom, Michael S. Seaman, Clara Lehmann, Nico Pfeifer, George Georgiou, Florian Klein

**Affiliations:** 1 Institute of Virology, Faculty of Medicine and University Hospital Cologne, University of Cologne, Cologne, Germany; 2 Department I of Internal Medicine, Faculty of Medicine and University Hospital Cologne, University of Cologne, Cologne, Germany; 3 Center for Molecular Medicine Cologne (CMMC), Cologne, Germany; 4 German Center for Infection Research (DZIF), partner site Bonn-Cologne, Cologne, Germany; 5 Department of Biomedical Engineering, University of Texas at Austin, Austin, TX USA; 6 Methods in Medical Informatics, Department of Computer Science, University of Tübingen, Tübingen, Germany; 7 Institute for Bioinformatics and Medical Informatics, University of Tübingen, Tübingen, Germany; 8 Research Group Computational Biology, Max Planck Institute for Informatics, Saarbrücken, Germany; 9 Saarland Informatics Campus, Saarbrücken, Germany; 10 Department of Molecular Biosciences, University of Texas at Austin, Austin, TX USA; 11 Molecular and Cellular Biology Graduate Program, University of Washington, and Basic Sciences Division, Fred Hutch Cancer Center, Seattle, WA USA; 12 Basic Sciences Division and Computational Biology Program, Fred Hutchinson Cancer Center, Seattle, WA USA; 13 Praxis am Ebertplatz, Cologne, Germany; 14 Gemeinschaftspraxis Gotenring, Cologne, Germany; 15 Praxis Hohenstaufenring, Cologne, Germany; 16 Infectious Diseases Division, Goethe University Frankfurt, University Hospital, Frankfurt am Main, Germany; 17 Vanderbilt Vaccine Center, Vanderbilt University Medical Center, Nashville, TN USA; 18 Department of Pathology, Microbiology and Immunology, Vanderbilt University Medical Center, Nashville, TN USA; 19 Department of Infection and Immunology, Kathmandu Research Institute for Biological Sciences, Lalitpur, Nepal; 20 Department of Dermatology, University Hospital Essen, University Duisburg-Essen, Essen, Germany; 21 Institute of Virology, Medical Faculty, University Bonn, Bonn, Germany; 22 Department of Microbiology, New York University School of Medicine, New York City, NY USA; 23 Department of Medicine, NYU Grossman School of Medicine, New York City, NY USA; 24 Vaccine Center, NYU Grossman School of Medicine, New York City, NY USA; 25 Medical Diagnostic Center, Yaoundé, Cameroon; 26 Chantal Biya International Reference Centre for Research on HIV/AIDS Prevention and Management (CIRCB), Yaoundé, Cameroon; 27 Department of Medicine, Division of Infectious Diseases, Icahn School of Medicine at Mount Sinai, New York City, NY USA; 28 Department of Microbiology, Icahn School of Medicine, New York City, NY USA; 29 Division of Infectious Diseases and Tropical Medicine, University Hospital, LMU Munich, Munich, Germany; 30 German Center for Infection Research (DZIF), Partner Site Munich, Munich, Germany; 31 Fraunhofer Institute for Translational Medicine and Pharmacology ITMP, Immunology, Infection and Pandemic Research, Munich, Germany; 32 Mbeya Medical Research Centre, National Institute for Medical Research, Mbeya, Tanzania; 33 Vanderbilt Institute for Infection, Immunology and Inflammation, Vanderbilt University Medical Center, Nashville, TN USA; 34 Department of Computer Science, Vanderbilt University, Nashville, TN USA; 35 Center for Structural Biology, Vanderbilt University, Nashville, TN USA; 36 Unit Global Health, Helmholtz Zentrum München, German Research Center for Environmental Health (HMGU), Neuherberg, Germany; 37 Department of Chemical Engineering, University of Texas at Austin, Austin, TX USA; 38 Howard Hughes Medical Institute, Seattle, WA USA; 39 Center for Virology and Vaccine Research, Beth Israel Deaconess Medical Center, Harvard Medical School, Boston, MA USA

**Keywords:** Infection, HIV infections, HIV infections

## Abstract

Human immunodeficiency virus type 1 (HIV-1)-neutralizing antibodies (nAbs) that prevent infection are the main goal of HIV vaccine discovery. But as no nAb-eliciting vaccines are yet available, only data from HIV-1 neutralizers—persons with HIV-1 who naturally develop broad and potent nAbs—can inform about the dynamics and durability of nAb responses in humans, knowledge which is crucial for the design of future HIV-1 vaccine regimens. To address this, we assessed HIV-1-neutralizing immunoglobulin G (IgG) from 2,354 persons with HIV-1 on or off antiretroviral therapy (ART). Infection with non-clade B viruses, CD4^+^ T cell counts <200 µl^−1^, being off ART and a longer time off ART were independent predictors of a more potent and broad neutralization. In longitudinal analyses, we found nAb half-lives of 9.3 and 16.9 years in individuals with no- or low-level viremia, respectively, and 4.0 years in persons who newly initiated ART. Finally, in a potent HIV-1 neutralizer, we identified lower fractions of serum nAbs and of nAb-encoding memory B cells after ART initiation, suggesting that a decreasing neutralizing serum activity after antigen withdrawal is due to lower levels of nAbs. These results collectively show that HIV-1-neutralizing responses can persist for several years, even at low antigen levels, suggesting that an HIV-1 vaccine may elicit a durable nAb response.

## Abstract (web summary)

Human immunodeficiency virus type 1 (HIV-1)-neutralizing responses can persist for several years, even at low antigen levels, suggesting that an HIV-1 vaccine may be able to elicit a durable antibody response.

## Main

Neutralizing antibodies (nAbs) block viral infection by directly binding circulating viruses, thus preventing these viruses from infecting their intended target cells^1^. In addition, they can eliminate infected cells through antibody-mediated effector functions^2^. As a result, nAbs are crucial to both preventing and overcoming viral infections and are considered one of the most important correlates of vaccine-mediated protection^3^.

In human immunodeficiency virus type 1 (HIV-1) infection, elicited circulating antibodies are mostly strain-specific and are not able to prevent replication of the broad diversity of viral strains that evolve in a person infected with HIV-1 (ref. ^4^). Yet, broadly neutralizing antibodies (bNAbs) targeting up to 99% of tested HIV-1 variants have been isolated from persons with an extraordinary HIV-1 neutralizing serum response (that is, elite neutralizers). These bNAbs have been demonstrated to prevent infection in humanized mice and in nonhuman primate models of HIV-1 infection^5–8^. Furthermore, a pooled analysis of two multicenter trials showed that intravenous infusions of the bNAb VRC01 were associated with reduced incidence of infection by VRC01-sensitive HIV-1 strains, although it did not show protection from HIV-1 acquisition overall compared to placebo^9^. Further analyses of these two studies revealed that serum titers and the potency against infecting strains of passively infused bNAbs correlate with protection from HIV-1 infection^10^. This indicates that effective HIV-1 protection could be provided by passive infusion of broader and more potent bNAbs, or combinations of such antibodies. Moreover, it indicates that a vaccine that is able to elicit potent and broad nAbs in humans at high titers could effectively protect from HIV-1 infection. Therefore, the induction of nAbs has become a main goal for vaccine development^11^.

As a result, several different nAb-inducing vaccine candidates and strategies are being developed and immunogens that aim to initiate bNAb development are currently being evaluated in early clinical trials^12–15^. For example, the germline-targeting priming vaccine candidate eOD-GT8 elicits VRC01-class B cell responses in humans^13^.

Longitudinal studies of elite neutralizers and HIV-1 neutralization screenings of large cohorts identified several factors associated with the development of a broad and potent neutralizing serum response^16–28^. Most of these factors reflect the exposure to antigen, such as high viral loads, a high viral diversity and specific HIV-1 subtypes, that were independent predictors for neutralization in a Swiss cohort of about 4,500 HIV-1-infected persons^21^.

However, most studies investigated neutralizing serum activity only in individuals who did not receive antiretroviral therapy (ART) and thus had high viral loads resulting in high antigen levels. Moreover, little is known about the dynamics and durability of the HIV-1 nAb responses. Compared to nAbs against, for example, severe acute respiratory syndrome coronavirus 2 (SARS-CoV-2) and most other viral pathogens, potent anti-HIV-1 nAbs are often highly mutated as well as occasionally autoreactive. These features are believed to present an obstacle for induction and may also be critical for the durability of protective HIV-1 nAb responses following vaccination^3,11^. Thus, information on the durability and dynamics of nAb responses in humans is crucial and highly relevant for future vaccine strategies.

Although the absence of a nAb-inducing vaccine prevents testing nAb longevity upon vaccination, other models need to be taken into account. In natural infection, individuals usually present high viral loads resulting in a continuous stimulation of the immune system and the nAb response. However, when HIV-1-infected persons start ART medication, viral replication is almost completely shut down following no detection of HIV-1 in the blood. Therefore, investigating the longevity of bNAbs after antigen withdrawal by ART medication can be important as this setting resembles the antigen dynamics of a desirable nAb-inducing vaccine in which the immunogen that leads to bNAb development vanishes over time^11^.

Here we report on the largest international study of HIV-1 neutralization dynamics, sampling over 2,300 individuals. By performing immunoglobulin G (IgG) isolation, we determined the neutralizing activity in participants on and off ART and assessed the association between different levels of viral replication and nAb responses^29^. We were thereby able to (1) determine factors driving IgG-neutralizing activity, (2) describe the neutralization activity of highly potent elite neutralizers and (3) longitudinally decipher the nAb activity in response to different viral load dynamics. Finally, we performed a detailed molecular analysis of monoclonal antibodies comprising the serological response over time in an HIV-1-infected individual with potent neutralizing serum activity after ART initiation.

## Results

### Cohorts show differing but not clade-specific neutralization

**Fig. 1:**
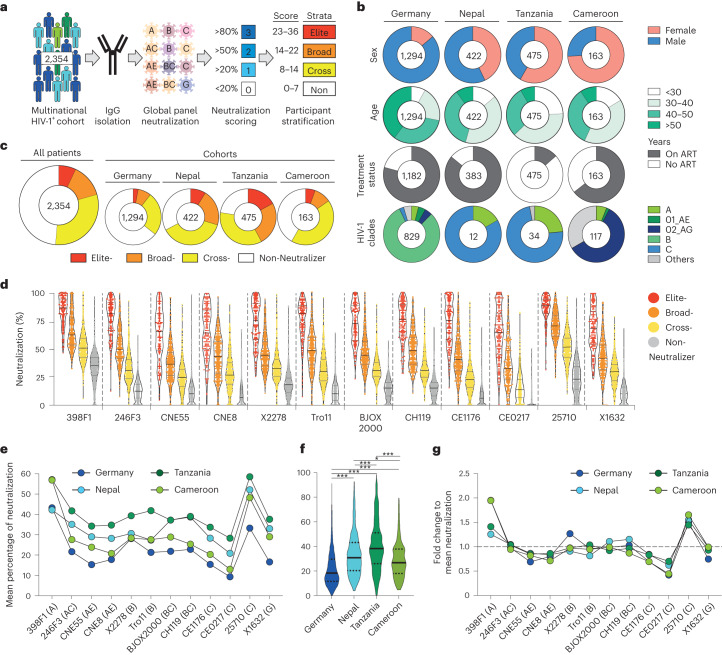
Neutralization screening of a large multinational cohort. **a**, Isolated IgGs of 2,354 HIV-1-infected individuals from a multinational study were tested against the global panel. The neutralization against each of the 12 viruses received a score between 0 and 3. Scores were later added to build neutralization strata. **b**, Clinical and virological characteristics of individuals from the different countries. Numbers indicate the amount of people for whom the respective characteristic was available. **c**, Neutralization strata of all individuals and of each respective cohort. **d**, Violin plots of the neutralization of participants’ IgGs against the global panel pseudoviruses. Individuals were stratified according to the strata as shown in **c**. Black bars indicate median, and gray bars indicate the respective quartiles. **e**, Average neutralization of each cohort against the respective 12 global panel viruses. **f**, Violin plots show the average neutralization against the whole global panel (Germany, *n* = 1,294; Nepal, *n* = 422; Tanzania, *n* = 475 and Cameroon, *n* = 163). Black bars indicate median, and dotted bars indicate the respective quartiles. One-way ANOVA with Tukey’s correction for multiple comparisons was used to compare average neutralization between groups. *P = 0.021, ***P < 0.001. **g**, Fold change of the mean neutralization of the respective cohorts against the global panel, when normalized to the mean activity against all 12 strains (**f**).

**Extended Data Fig. 1:**
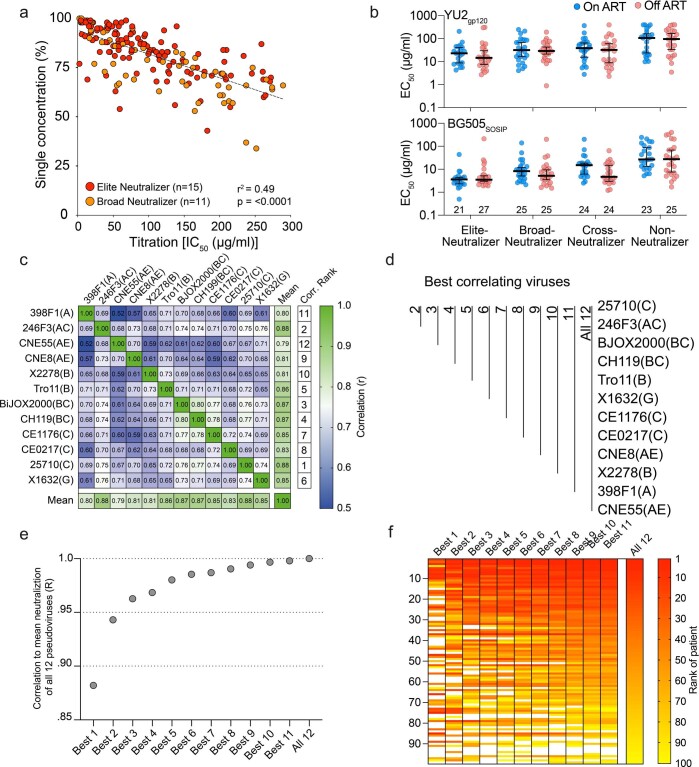
Validation and optimization of single concentration neutralization screening. (**a**) Global panel IgG neutralization of patient IgGs from elite- and broad neutralizers. Each dot represents neutralization of a single individual against a single pseudovirus. X-axis shows the 50% inhibitory concentration (IC_50_) values that were determined by IgG titration. Y-axis shows percent inhibition at 300 µg/ml. R2 was calculated using the Pearson correlation coefficient. P-value is two-tailed. (**b**) Top and bottom graphs show binding against YU2_gp120_ and BG505_SOSIP.664_, respectively, as measured by ELISA. Numbers indicate the number of individuals tested per group. Black bars indicate the median with the 95% confidence intervals. (**c**) Pearson’s correlation of the neutralization screening results between all 12 global panel strains (middle section) and between each strain to the mean of all global panel strains among all 2,354 tested individuals. Viruses were ranked from best to worst correlation of the single respective virus to the mean of all 12 viruses. (**d**) Overview of the combinations of the best correlating viruses as identified in **c**. (**e**) Pearson’s correlation of the mean of combinations of the best correlating viruses as depicted in **c** and **d** to the mean neutralization against all 12 pseudoviruses in 2,354 tested individuals. (**f**) Ranking of individuals according to the reduced pseudovirus sets. Individuals were ranked by their mean neutralization results against the best correlating virus mixes. Right bar indicates the ranking of individuals according to their mean neutralization against all 12 viruses. This ranking also sets the color per individual in the other ranking (white bars: individuals not among the top 100 participants when using mean of all 12 viruses).

To study the neutralizing activity in a large multinational cohort, we tested samples from 2,354 individuals infected with HIV-1 from Central Europe, Central and Eastern Africa and Asia for their HIV-1-neutralizing activity. Participants’ IgG antibodies were isolated from serum or plasma and tested against a panel of 12 pseudoviruses (global panel) that is representative of the worldwide circulating HIV-1 strains (Fig. 1a)^30^. Isolation of IgG prevents other plasma factors from interacting with the neutralization assay and thus allowed us to also test individuals that were on ART at study inclusion. The majority of individuals were sampled in Germany (*n* = 1,294), followed by Tanzania (*n* = 475), Nepal (*n* = 422) and Cameroon (*n* = 163; Fig. 1b). Neutralization was tested at a single participant’s IgG concentration of 300 µg ml^−1^ against each respective virus. The resulting percentage neutralization of this single-concentration high-throughput approach significantly correlated with the 50% inhibitory concentration (IC_50_) values that were determined by IgG titration in a subset of 26 elite- and cross-neutralizers (*r*^2^ = 0.49, *P* < 0.001; Extended Data Fig. 1a). The IgG neutralization of the 12 pseudoviruses was assigned individual scores between 0 and 3 points based on their potency against the respective virus. These points were finally added up to describe each individual’s overall IgG neutralization activity, taking into account its potency and breadth. This allowed us to stratify individuals into non-neutralizer (*n* = 1146, 48.6%), cross-neutralizer (*n* = 722, 30.6%), broad neutralizer (*n* = 165, 7.0%) or elite neutralizer (*n* = 165, 7.0 %) categories (Fig. 1a,c). In line with their neutralizing activity, we also found a more potent IgG binding against viral envelope proteins in elite neutralizers compared to participants with a weaker neutralizing activity (Extended Data Fig. 1b). Of note, the average neutralizing activity against only three viruses (25710, 246F3 and BJOX2000) is highly predictive for the global panel neutralization (*r*^2^ = 0.93, *P* < 0.001) and would have identified 19 of the 24 top 1% neutralizing individuals of our cohort (Extended Data Fig. 1c–f). Taken together, the use of our single-concentration approach on a lower number of viruses might facilitate screening of larger cohorts in the future.

The distribution of neutralizing activities significantly varied per cohort with Tanzania having the largest fraction of elite neutralizers (17.0%), followed by Nepal, Cameroon and Germany (8.8%, 5.5% and 2.9%, respectively, *P* = 0.01; Fig. 1c). The performed stratification allowed for discrimination of the neutralizing activity between non-neutralizer, cross-neutralizer, broad neutralizer and elite neutralizer against all tested viruses (*P* = <0.0001 for all 12 viruses; Fig. 1d). Interestingly, even though the average neutralization against the global panel was significantly different between cohorts (Fig. 1e,f), the fold change of the neutralization against each respective global panel strain compared to the average neutralization was similar between the different cohorts, resulting in a comparable neutralization pattern (Fig. 1g). Thus, the differing clinical and virological characteristics between our cohorts did not have an effect on the IgG neutralization pattern. In particular, the difference in predominantly circulating strains (predominant clade (% of total); Germany, clade B (81%); Nepal, clade C (83%); Tanzania, clade C (75%) and Cameroon, clade 02-AG (59%)) did not shape the neutralization pattern against the multiclade global panel.

### Stronger neutralization linked to increased CD4bs targeting

**Fig. 2:**
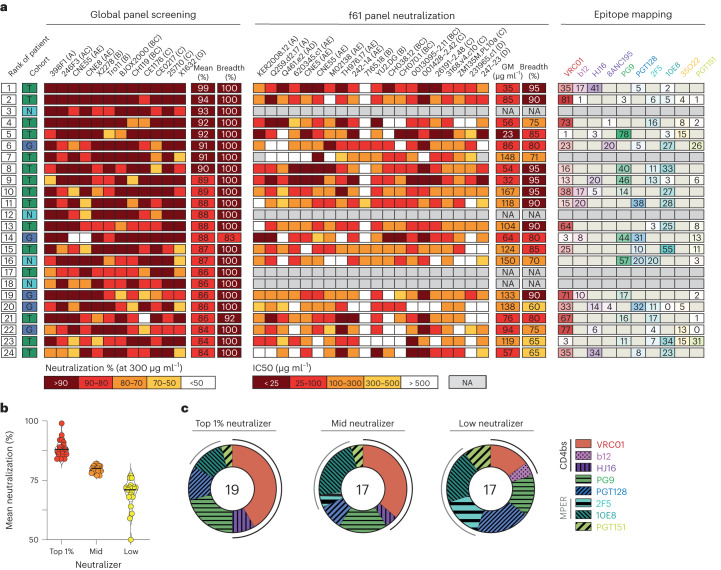
Identification of elite neutralizers with extremely potent and broad serum activity. **a**, Top 1% (*n* = 24) of all 2,354 tested individuals after ranking them according to their mean neutralization (%) against all 12 pseudoviruses. Left, results of the single dilution screening against the global panel. First column indicates the ranking of each individual. Second column indicates the respective cohort (green, Tanzania (T); turquoise, Nepal (N) and blue, Germany (G)). Middle, neutralization activity (IC_50_ (µg ml^−1^)) against the f61 fingerprint panel. Right, delineation scores of f61-panel-based computational epitope mapping. Values are rounded, which may result in totals of 101 or 99 for some lines/individuals. **b**, Strata that were used for epitope analysis in **c**. Shown is the average neutralizing activity against the global panel of 53 persons (top neutralizer, *n* = 19; mid neutralizer, *n* = 17 and low neutralizer, n = 17). Black bars show median average neutralization. Dotted bars show quartiles. **c**, Pie charts showing the number of individuals that were stratified as top-, mid- or low-neutralizers, based on their average neutralization (**b**). Slices represent the mapped epitopes. Delineation scores >30% were counted as mapped epitope per individual. Outer lines indicate epitope mapping toward the CD4-binding site (black bar) or the MPER region (gray bar). GM, geometric mean.

After ranking individuals based on their IgG-neutralizing activity, the best 1% of all screened 2,354 individuals (*n* = 24) were found to have an exceptionally broad (breadth of 83–100%) and potent (mean neutralization at 300 µg ml^−1^ of 84–99%) neutralizing IgG activity (Fig. 2a). The top neutralizing serum in our cohort was from a participant from Tanzania and neutralized all global panel strains with a mean neutralization at 300 µg ml^−1^ of 99%.

**Extended Data Fig. 2:**
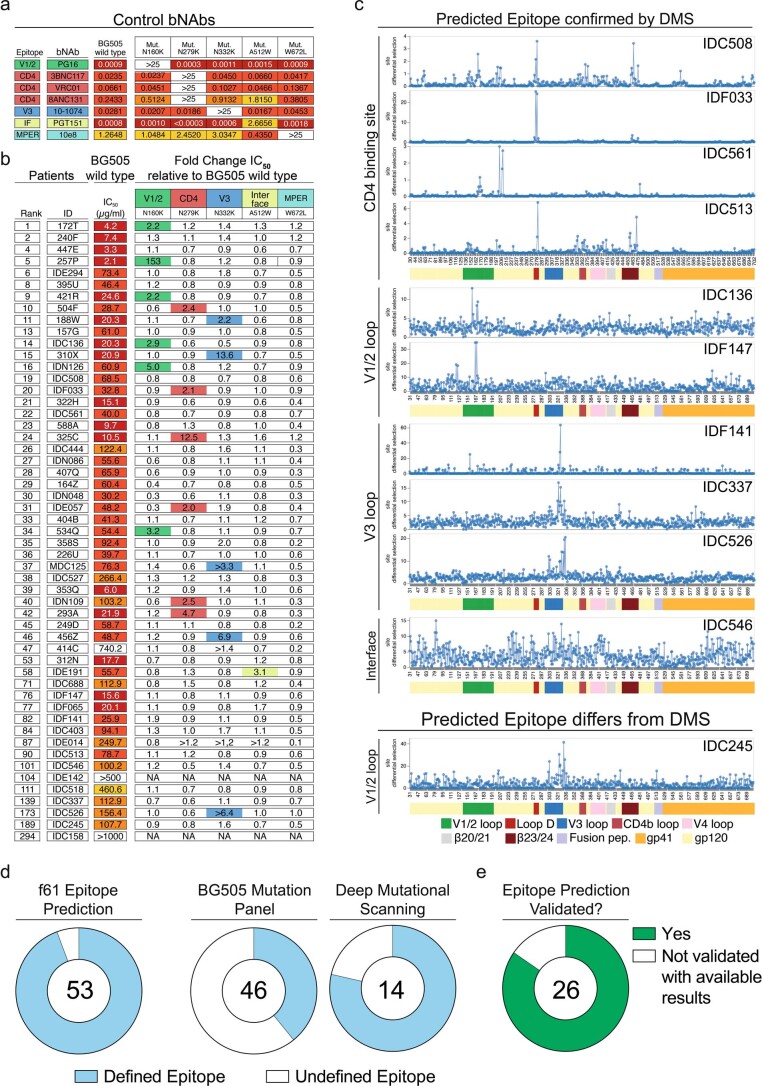
Validation of f61-panel based epitope predictions. (**a**) Broadly neutralizing antibody (bNAb) IC_50_ values (µg/ml) against wild type and mutated BG505_T332N_ pseudovirus. First column indicates the epitopes of the respective tested bNAbs. (**b**) Participants’ IgG IC_50_ values (in µg/ml) against wildtype and mutated BG505_T332N_ pseudovirus. First column indicates the neutralization rank of each individual among the 2,354 HIV infected individuals, followed by each participant’s IDs and each individuals IgGs IC_50_ (µg/ml) against wild type BG505_T332N_. Right panel shows the change in IgG sensitivity (fold change of IC_50_) for BG505_T332N_ mutants compared with the wild type. Defined epitopes (fold change of ≥2 against a mutated BG505_T332N_ pseudovirus) were marked in the respective colors. (**c**) Escape at all sites from participants’ IgGs in the BF520 Env ectodomain (for participants IDC508, IDF003, IDC561 and IDC513) as defined by the lentivirus deep mutational scanning assay and in the BG505 Env ectodomain (all other participants) as defined by the HIV DMS assay. Positive values represent sites where mutations cause escape. Prediction of f61-panel of the respective participant is shown on the left. Colored panel shows the respective epitopes of Env. The numbers at the bottom of each panel indicate the HIV-1 Env residues numbered according to HIV-1_HXB2_. IDC561 shows escape at binding sites of the CD4 binding site bNAb 1–18, isolated from this individual. Thus, IDC561 was counted as individual for which the predicted epitope was confirmed. (**d**) Pie charts show the results from the f61-panel based computational epitope prediction (left), from the BG505 mutation panel (middle) and from the deep mutational scanning (DMS, right). Slices indicate the fraction of individuals. Predicted scores >30% in f61 panel count as ‘defined’. Numbers indicate n of individuals tested. For the BG505 mutation panel, individuals for whom the f61 epitope prediction did not result in a defined epitope (n = 3) and individuals with only low potency against BG505 wild type (n = 4) were excluded for this analysis. (**e**) Pie chart shows combined results from the experimental validation of the f61 panel-based prediction by either the BG505 mutation panel or DMS. A predicted epitope was counted as validated when the epitope with highest f61 delineation score in each individual was also identified by at least one of the other assays. Slices indicate the fraction of individuals. Number indicates the number of individuals in whom f61-based prediction and at least one of the BG505 mutation panel or DMS resulted in a defined epitope.

IgG epitope mapping in 19/24 of the top 1% neutralizers revealed that a larger fraction of the neutralizing responses in these individuals was directed against the CD4-binding site (CD4bs). Of all calculated epitopes, 41.7%, 8.3% and 0% mapped as the CD4bs bNAbs VRC01, HJ16 and b12, respectively (Fig. 2a). Other epitopes were targeted less frequently with 21% targeting the V1/V2-loop (PG9-like), 13% targeting the V3-glycan (PGT128-like) and 13% targeting the membrane-proximal external region (MPER; 10E8-like). We further compared the IgG epitope mapping of these top neutralizers (*n* = 19) with the mapped targets of serum antibodies in two groups of individuals with weaker neutralizing activity (mid neutralizer: *n* = 17 and low neutralizer: *n* = 17; Fig. 2b and Supplementary Table 1). At lower serum IgG-neutralizing levels, the proportion of CD4bs-directed neutralizing responses decreased while the proportion of MPER-targeting responses increased (CD4bs-directed bNAb activity: top: 50%, mid: 36%, low: 20%; MPER-directed bNAb activity: top: 13%, mid: 27%; low: 35%, *P* < 0.001; Fig. 2c). These IgG specificities were computationally predicted based on each participant’s IgG activity against the f61 fingerprint panel. To validate these predictions, we further tested participant’s IgGs against a panel of mutated BG505_T332N_ pseudoviruses and in deep mutational scanning (DMS) assays (Extended Data Fig. 2a–c)^31,32^. Using these methods, we identified the targeted epitope in 26 individuals (epitope identified by BG505 panel: 18 of 46 (39%) participants; by DMS: 11 of 14 (79%) participants; epitope for three participants was identified using both methods); in 22 (85%) individuals, the herein identified targeted epitope matched the computationally predicted epitope (Extended Data Fig. 2d,e).

In conclusion, neutralizing IgG serum activity of top neutralizing individuals shows a strong preference for targeting the CD4bs, while the targeted epitopes in lower tier neutralizing individuals appear more evenly distributed over the HIV-1 envelope.

### Antigen exposure and clade determine neutralizing activity

**Fig. 3:**
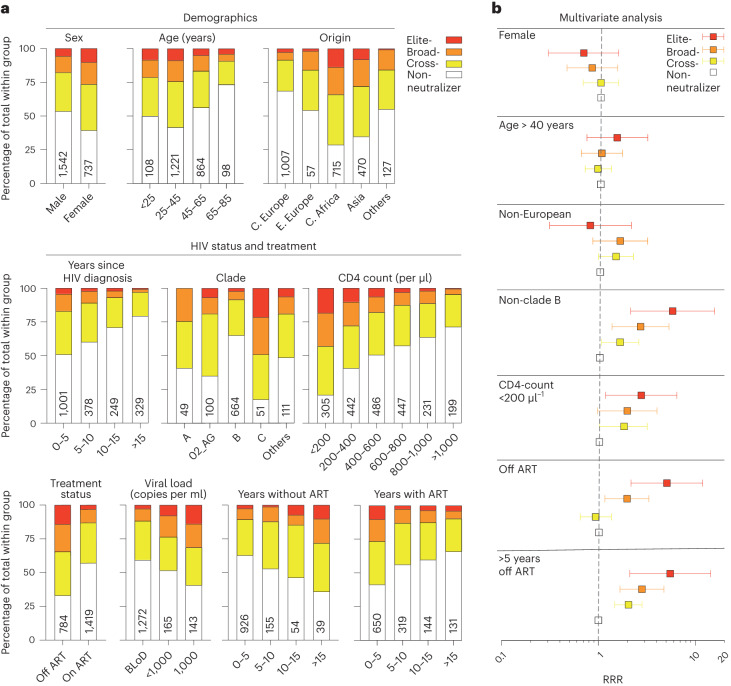
HIV-1 infection history determines the neutralizing activity. **a**, Clinical characteristics of individuals. All individuals for whom the specific clinical information was available were included in the respective analysis. Columns indicate the percentage of elite neutralizer, broad neutralizer, cross-neutralizer or non-neutralizer in each respective group. Numbers in columns indicate the size of the respective group (C. Europe, Central Europe; E. Europe, Eastern Europe; C. Africa, Central Africa; BLoD, below the limit of detection). **b**, Multinomial logistic Cox-regression model to assess the impact of demographic characteristics and HIV-1 infection history on the neutralization capacity. Individuals for whom all factors were available have been included in this analysis (*n* = 942). Squares indicate the RRR together with the 95% confidence intervals.

We further assessed which clinical and virological factors influence the development of IgG neutralization activity. In an exploratory analysis, we found females, people of younger age and people from Asia or Central Africa to be more likely to have detectable neutralizing serum activity (Fig. 3a). Furthermore, a shorter time since HIV-1 diagnosis, infection with a clade C strain, a lower CD4^+^ T cell count, not receiving ART, higher viral loads, a longer period without ART and a shorter period with ART since their first HIV-1 diagnosis was associated with a more active neutralizing IgG serum response (Fig. 3a). To exclude the interdependency of these variables and determine independent predictors for developing a neutralizing response, we used a multinomial logistic Cox-regression model using key variables of the explorative analysis in a subset of individuals with all variables available (*n* = 942). This model found infection with a non-clade B strain (relative risk ratio (RRR): 5.83, 95% confidence interval (95% CI): 2.15–15.88), a CD4^+^ T cell count below 200 µl^−1^ (RRR: 2.71, 95% CI: 1.16–6.32), being off ART at study inclusion (RRR: 5.00, 95% CI: 2.13–11.73) and a longer period of being off ART before study inclusion regardless of the current ART status (RRR: 5.52, 95% CI: 2.11–14.44) to be strong independent predictors for neutralization activity (Fig. 3b).

In summary, we found a strong association of non-clade B strains and of a longer antigen exposure with IgG neutralization activity. Interestingly, three variables—CD4^+^ T cell count, the current as well as the former treatment status—that depend on ART intake were independently associated with serum IgG-neutralizing activity in our cohort.

### Viral dynamics determine the durability of HIV-1 neutralization

**Fig. 4:**
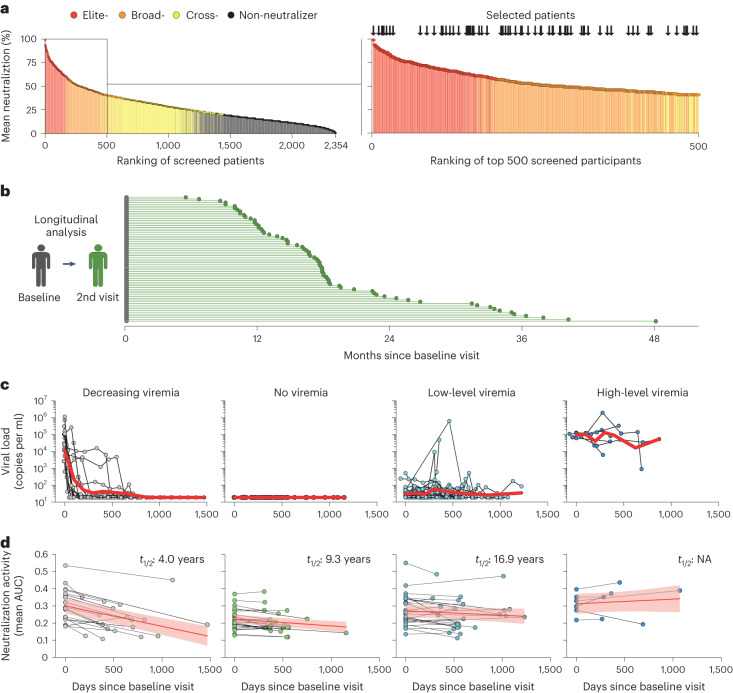
Durability of HIV-1 neutralization is determined by the amount of antigen exposure. **a**, Left, the ranking of all tested persons (*n* = 2,354) according to their average neutralization against all global panel strains in the screening. Colors refer to the respective neutralization strata of each person. Right, the top 500 persons. Arrows indicate persons that have been selected for further analysis (*n* = 71). **b**, Time in months between baseline visit (gray dots) and second visit (green dots). **c**, HIV-1 RNA plasma copies (copies per ml) of individuals between baseline and second visit. Individuals were grouped based on the cumulative viral load of each individual over time (decreasing viremia, *n* = 18; no viremia, *n* = 22; low-level viremia, *n* = 25 and high-level viremia, *n* = 6). Red lines show the geometric mean of viral loads. **d**, Neutralization dynamics of individuals between baseline and second visit. Dots are the mean AUCs of the in vitro IgG neutralization curves against the global panel pseudoviruses at each respective visit. Each pair of dots represents the neutralization dynamics of one person. Red lines show a learned linear mixed model describing AUC changes between the baseline and second visit. Light red areas indicate the 95% confidence bands. Half-lives (*t*_1/2_) were calculated from the slope of separate mixed models fitted to the binary logarithm of the mean AUC.

**Extended Data Fig. 3:**
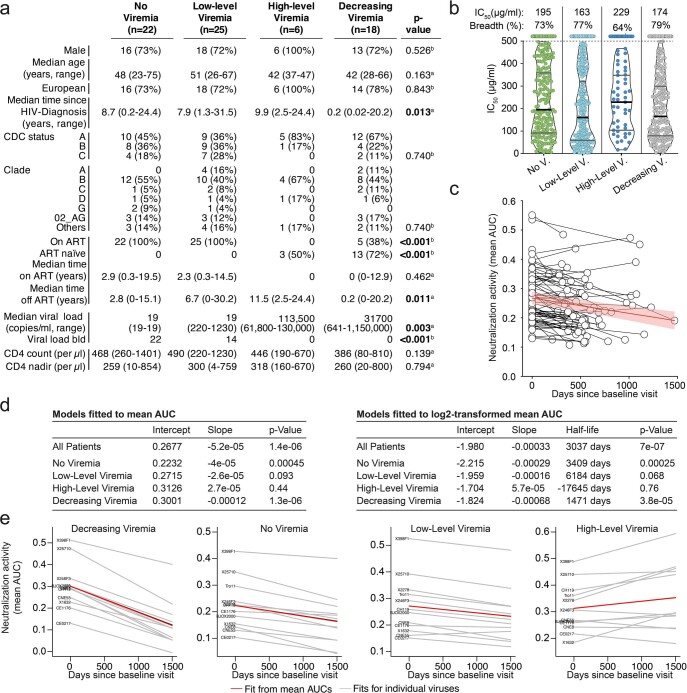
Durability of HIV-1 neutralization is determined by the amount of antigen exposure. (**a**) Clinical characteristics of the groups that were selected by differing viremia over time. P-values marked with ‘a’ were calculated by one-way ANOVA. P-values marked with ‘b’ were calculated using Pearson’s Chi-square test. (**b**) Neutralization activity against the global panel. Each dot represents a single participant’s IgGs against one virus. Black bars indicate the median. Gray bars indicate the upper and lower quartile. Depicted IC_50_ values at the top indicate the median IC_50_ of all individuals against all viruses. Breadth values indicate the median neutralization breadth of all individuals in the respective group. No significant differences were found between the median IC_50_s between each respective group (p > 0.05). (**c**) Neutralization dynamics of individuals between baseline and 2^nd^ visit. Dots are the mean areas under the curves (AUC) of the *in vitro* IgG neutralization curves against the global panel pseudoviruses at each respective visit. Each pair of dots represent the neutralization dynamics of one participant. The red line shows a learned linear mixed model describing AUC changes between baseline- and 2nd visit. Light red areas indicate the 95% confidence bands. (**d**) Slope indicates the daily increase (positive values) or decrease (negative values) of neutralization activity in the respective groups. P-values indicate the significance of a comparison between the here used model of AUC changes over time and a model that ignores the time variable (R-function stats::anova). The left table shows models fitted to the mean AUC, which are used for visualization. The right table shows models fitted to the log2-transformed mean AUC, which are used for the calculation of half-life estimates. (**e**) Gray lines show learned linear mixed models describing AUC changes between baseline- and 2^nd^ visit per virus. Red lines show the mean fitted line of all viruses.

**Extended Data Fig. 4:**
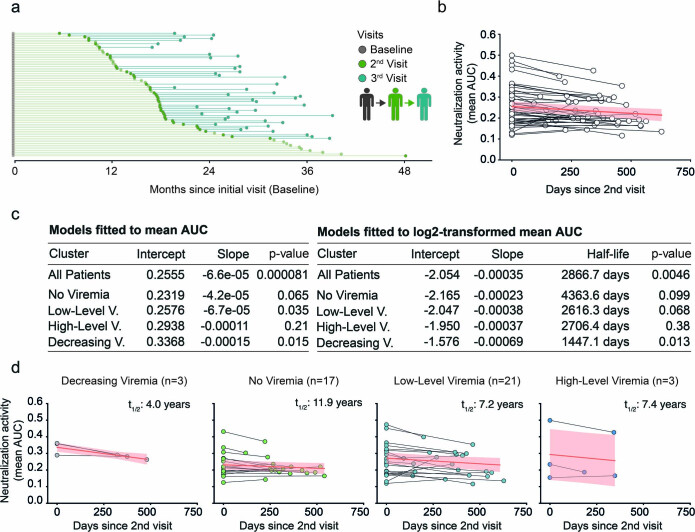
HIV-1 neutralization dynamics between 2^nd^ and 3^rd^ visit. (**a**) Time in months between baseline visit (gray dots), 2^nd^ visit (green dots) and 3^rd^ visit (turquoise dots). 44 individuals had blood draws at 3 visits. (**b**) Neutralization dynamics of individuals between 2^nd^ and 3^rd^ visit. Dots are the mean areas under the curves (AUC) of the IgG neutralization curves against the global panel pseudoviruses at each respective visit. Each pair of dots represent the neutralization dynamics of one individual. The red line shows a learned linear mixed model describing AUC changes between 2nd and 3rd visit. Light red areas indicate the 95% confidence bands. (**c**) Fitted linear mixed models in detail. Slope indicates the daily increase (positive values) or decrease (negative values) of neutralization activity in the respective groups. P-values indicate the significance of a comparison between the here used model of AUC changes over time and a model that ignores the time variable (R-function stats::anova). The left table shows models fitted to the mean AUC, which are used for visualization. The right table shows models fitted to the log2-transformed mean AUC, which are used for the calculation of half-life estimates. (**d**) Neutralization dynamics of participants between 2^nd^ visit and 3^rd^ visit. Dots are the mean areas under the neutralization curves (AUC) of the *in vitro* IgG neutralization curves against the global panel pseudoviruses at each respective visit. Each pair of dots represent the neutralization dynamics of one participant. Red lines show a learned linear mixed model describing AUC changes between baseline- and 2^nd^ visit. Light red areas indicate the 95% confidence bands. Half-lifes (t1/2) were calculated from the slope of separate mixed models fitted to the binary logarithm of the mean AUC.

In the vast majority of individuals, ART intake determines HIV-1 replication and therefore serum antigen levels. Given that the history of ART intake had such a profound effect on the IgG-neutralizing activity, we were interested in knowing how longitudinal antigen dynamics influence the IgG-neutralizing activity. To this end, we longitudinally collected samples of 71 randomly selected elite and broad-neutralizing participants to test them for their IgG-neutralizing activity (Fig. 4a, Extended Data Fig. 3a and Supplementary Table 2). Median time from the baseline visit to the second and third study visit (third visit was available for 44 of 71 individuals) was 1.5 and 2.3 years, respectively (Fig. 4b and Extended Data Fig. 4a). Based on their plasma viral loads that were determined on and between the sampling timepoints, neutralizers were grouped into people that had no viremia in between timepoints (*n* = 22), low-level viremia (*n* = 25), high-level viremia (*n* = 6) or a decreasing viremia after ART initiation (*n* = 18; Fig. 4c). Other clinical characteristics and IgG-neutralizing activities at baseline visit were comparable between these four groups (Extended Data Fig. 3a,b). Neutralizing activity was compared between timepoints by measuring the area under the neutralization curve (AUC) for an individual’s isolated IgG against each virus of the global panel (Supplementary Tables 3 and 4). Between the first two visits, the neutralization serum IgG activity of the whole cohort of 71 participants declined with a calculated half-life of 8.3 years (slope −0.000052; Extended Data Fig. 3c,d). However, the comparison of neutralization dynamics in the different antigen-level groups revealed substantial differences. With increasing amounts of antigen between the two visits, we found a slower decline (no viremia group: slope −0.00004; low-level viremia group: slope −0.000026), and in the case of the high-level viremia group, an increasing neutralization activity (slope 0.000027). Interestingly, the strongest decline in neutralizing serum IgG activity over time was found in the group with decreasing viremia (Fig. 4d and Extended Data Fig. 3d,e). The decline of neutralizing activity in this group (slope −0.00012) led to a calculated half-life of IgG-neutralizing activity of 4.0 years, compared to 9.3 and 16.9 years in the nonviremic and low-level viremic groups, respectively (Fig. 4d and Extended Data Fig. 3d). The weaker mean IgG-neutralizing activity over time in these groups was also found when the activity against each single virus was analyzed (Extended Data Fig. 3e). Moreover, similar results were found between the second and third visit, where we identified a further decline in neutralizing activity of the overall cohort (Extended Data Fig. 4a,b) as well as in the different groups (Extended Data Fig. 4c,d).

We conclude that IgG-neutralizing activity shows a strong initial decline after suppression of viremia due to ART intake. In contrast, a relatively stable neutralizing activity with decade-long half-lives was found in individuals with no viremia or low-level viral replication.

### Fewer nAb-encoding B cells post viremia suppression in IDC561

**Fig. 5:**
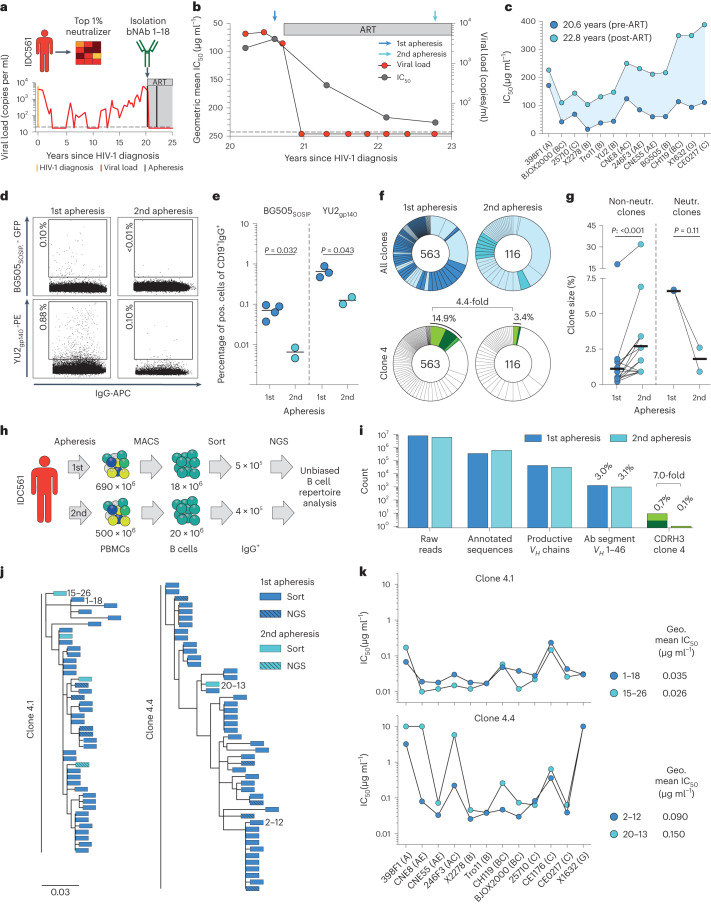
Neutralization dynamics in elite neutralizer IDC561. **a**, Clinical data of individual IDC561. **b**, Mean IgG neutralization of IDC561’s IgG against an extended global panel (12 strains + YU2 + BG505) and viral loads over time. Dotted line indicates the limit of viral detection. **c**, Neutralization activity of IDC561’ IgG from the two apheresis timepoints against the extended global panel (excluding CE1176). **d**, Representative dot blots of BG505_SOSIP.664_- (top) or YU2_gp140_-reactive (bottom) IgG^+^ B cells at first and second apheresis, respectively. Numbers indicate the frequency of HIV-1 envelope-reactive IgG^+^ B cells from the parental gate. **e**, Frequency of BG505_SOSIP.664_- (top) or YU2_gp140_-reactive IgG^+^ B cells from the parental gate as found in individual sorts (BG505: first apheresis, *n* = 4 and second apheresis, *n* = 2; YU2: first apheresis, *n* = 3 and second apheresis, *n* = 2). Lines indicate mean percentage. Two-tailed *t*-test was used for comparison. **f**, Pie charts show the numbers of clonal heavy-chain sequences identified. Individual clones are represented by slices. Light blue slices: clones that have been identified at both aphereses. Green slices: clone 4. **g**, Clone size (% of all identified sequences) of clones identified at both timepoints (blue dots, first apheresis and turquoise dots, second apheresis; non-neutralizing clones, *n* = 27 pairs and neutralizing clones, *n* = 2 pairs). Black lines show mean percentage. Wilcoxon matched-pairs signed rank test and two-tailed *t*-test were used to compare non-neutralizing and neutralizing clones, respectively. **h**, Scheme of sample processing for the unbiased B cell repertoire analysis. **i**, Columns show the total count of sequences that resulted from our NGS-based unbiased B cell repertoire analysis. **j**, Phylogenetic tree of clones 4.1 and 4.4 members as identified by different methods from the first and second apheresis timepoints. Antibodies 1–18, 15–26, 2–12 and 20–12 are further shown in **k**. **k**, Activity of bNAbs identified from individual IDC561 against the global panel. Antibodies 1–18 and 2–12 were identified from the first apheresis. Antibodies 15–26 and 20–13 were identified from the second apheresis. Dots at 10 µg ml^−1^ indicate nonneutralized strains (IC_50_ > 10 µg ml^−1^). Geo, geometric; Neutr., neutralizing.

To determine the cause for the decrease in neutralizing activity following ART-mediated viral suppression, we conducted a detailed molecular analysis of the dynamics of bNAb-encoding B cell clones in elite neutralizer IDC561, a patient with an outstanding serum neutralization activity. After 21 years of untreated HIV-1 infection, he received his first apheresis from which the highly broad and potent CD4bs bNAbs 1–18 and 2–12, both belonging to the ‘clone 4’-family, have been isolated (Fig. 5a)^33^. Clone 4 (with subbranches 4.1, 4.2, 4.3 and 4.4) includes a large number of bNAbs and was shown earlier to mediate IDC561’s exceptional serum IgG-neutralizing activity^33^. IDC561 initiated ART 2 months after his first apheresis and received a second apheresis 2.0 years after ART initiation (Fig. 5b). Thus, IDC561 allowed us to longitudinally track the dynamics of bNAb clone 4 after ART-mediated suppression of viremia on a highly detailed level.

**Extended Data Fig. 5:**
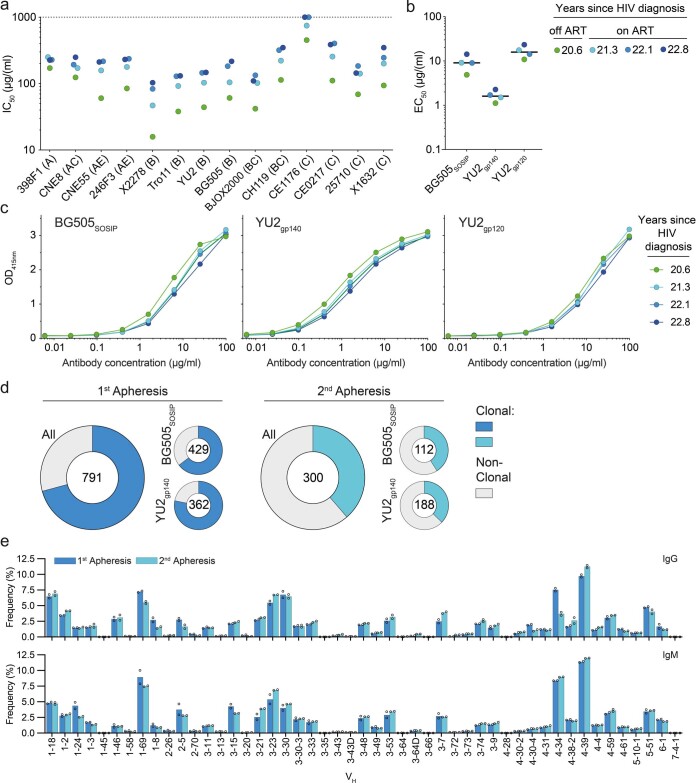
Neutralization dynamics in elite neutralizer IDC561. (**a**) Neutralization activity of IDC561 IgG against the global panel, YU2 and BG505 at four different time points. (**b**) Half maximal effective concentration (50% effective concentration (EC_50_)) of individual IDC561’s IgGs from different timepoints against the indicated HIV-1 Env antigens as determined by ELISA. Each dot represents the mean EC_50_ of two individual experiments. Line indicates the median EC_50_. (**c**) ELISA binding curves of (B) (**d**) Large pie charts show the numbers of heavy-chain sequences identified by single cell sorting. Smaller pie charts indicate the amount of total and clonal heavy-chain sequences as identified by single cell sorting with the respective HIV-1 Env bait. (**e**) Comparison of total B cell repertoire of IDC561 at the 1st and 2nd apheresis timepoints. Top panel shows frequency of IgG heavy chain variable region (VH)-genes. Bottom panel shows IgM VH-genes. Dots show results of replicates for which two independently sorted batches of IgG+ and IgM+ B cells, respectively, were used.

**Extended Data Fig. 6:**
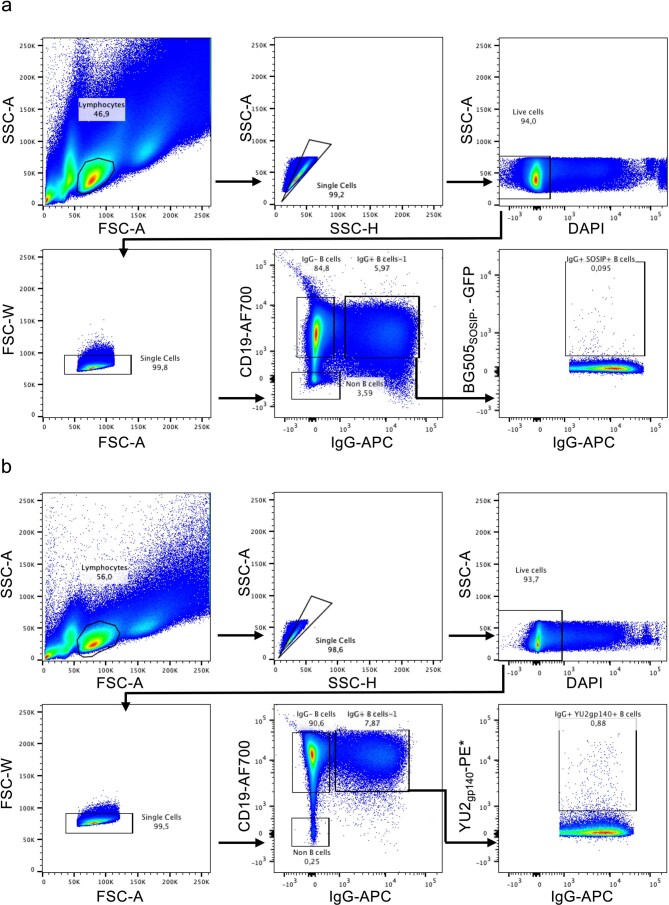
Gating strategy for HIV-1 specific single b cell sorting. Example of the gating strategy used for a single cell sorting of BG505_SOSIP.664_- (**a**) or YU2_gp140_-reactive (**b**) IgG^+^ b cells. * biotinylated YU2_gp140_ labeled with Streptavidin-PE.

After ART initiation, the serum IgG-neutralizing activity became less potent over time against all tested viral strains while IgG binding to HIV-1_Env_ only changed slightly (Fig. 5b,c and Extended Data Fig. 5a–c). We identified a significantly lower percentage of BG505_SOSIP.664_- or YU2_gp140_-reactive memory B cells after ART initiation (mean % of bait-binding, CD19^+^IgG^+^ B cells: BG505_SOSIP.664_: first apheresis: 0.07%, second apheresis: 0.01%, *P* = 0.03; YU2_gp140_: first apheresis 0.65%, second apheresis 0.13%, *P* = 0.04; Fig. 5d,e and Extended Data Fig. 6a,b). HIV-1_Env_ reactive IgG^+^ cells were sorted in a single-cell manner and subjected to *V*_*H*_ (immunoglobulin G heavy-chain variable region)-gene amplification and sequencing. Among all identified heavy-chain sequences, we identified 563 (71%) and 116 (39%) clonal B cells in the first and second apheresis samples that were assigned to 91 and 40 clones, respectively (Fig. 5f and Extended Data Fig. 5d). Of those, 32 clones shared B cell members identified at both apheresis timepoints. Clones that only consisted of B cells identified at a single timepoint were significantly more often found in the first apheresis sample (unique clones versus clones identified at both timepoints: first apheresis: 59 versus 32 clones; second apheresis 8 versus 32 clones, *P* = <0.001; Fig. 5f). We further tested 29 of the 32 shared clones for their neutralizing activity by testing at least one prototypic members of each clone and found different dynamics of the clone sizes between neutralizing and non-neutralizing clones. While shared non-neutralizing clones (defined as clones that neutralize fewer than four of the global panel strains) significantly increased in their relative frequency, the fraction of shared neutralizing clones, which both originated from the clone 4 lineage (clones 4.1 and 4.4), was reduced (Fig. 5g). Most importantly, among all identified clonal heavy chains, the clone 4 proportion significantly dropped by 4.4-fold from 14.9% to 3.4% after initiation of ART (clone 4 members versus nonclone 4 members: first apheresis: 84 versus 479; second apheresis 4 versus 112, *P* = <0.001; Fig. 5f).

Next, we performed an unbiased next-generation sequencing (NGS) approach to longitudinally track changes in the composition of the B cell repertoire. To this end, we applied *V*_*H*_-gene NGS to 450,000 and 400,000 bulk-sorted antigen-experienced (CD20^+^IgG^+^IgM^−^IgD^−^CD27^−^) and naïve (CD20^+^IgM^+^IgD^+^CD27^−^IgG^−^) B cells from the first and second apheresis, respectively (Fig. 5h). In the antigen-experienced B cell repertoire, we observed comparable frequencies of B cells from the *V*_*H*_ 1–46 gene segment (first apheresis: 1286 (3,0%); second apheresis: 989 (3,1%); Fig. 5i and Extended Data Fig. 5e) but, in line with the single HIV-1_Env_-reactive B cell-sequencing results, we found a significantly higher fraction of clone 4 members before the start of ART (clone 4 lineage cells versus other immunoglobulin G heavy-chain variable region (IGHV) 1–46 cells: first apheresis: 9 versus 1277 (0.07%); second apheresis: 1 versus 988 (0.01%), *P* = 0.032; Fig. 5i).

Of note, the clones 4.1 and 4.4 lineage bNAbs identified after ART start had highly similar sequential features compared with clone 4 bNAbs identified from pre-ART samples (Fig. 5j). Moreover, they showed a similar neutralization activity against the global panel (Fig. 5k)

In summary, suppression of viremia due to initiation of ART resulted in declining IgG-neutralizing serum activity in this participant, which is accompanied by a significantly lower fraction of circulating bNAb-encoding B cell clones.

### Anti-HIV-1 serum bNAbs decrease after the suppression of viremia

**Fig. 6:**
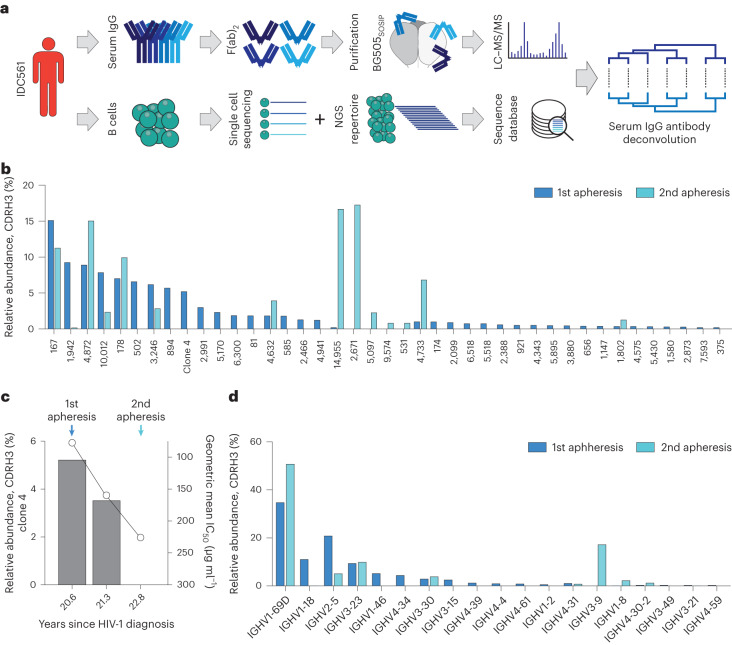
Longitudinal serum antibody repertoire of IDC561. **a**, Deconvoluting the serum antibody repertoire with the combination of LC–MS/MS and sequential data sets. **b**, CDRH3 clonotypic composition and relative frequency of the anti-BG505_SOSIP.664_ plasma antibody repertoire. **c**, Time course of the relative abundance of clone 4 in IDC561 anti-BG505_SOSIP.664_ plasma (left *y* axis) and IgG neutralization activity against the global panel (right *y* axis). **d**, IGHV-gene usage of the anti-BG505_SOSIP.664_ serum IgG repertoire of individual IDC561.

To test if the declining fraction of clone 4-encoding memory B cells is paralleled by lower amounts of circulating clone 4 serum bNAbs, we deconvoluted the circulating serum IgG antibody repertoire by bottom-up liquid chromatography-tandem mass spectrometry (LC–MS/MS) proteomics of antigen-specific serum antibodies using the Ig-seq pipeline^34,35^. In brief, anti-BG505_SOSIP.664_ IgGs from plasma samples were digested into peptides and subjected to high-resolution LC–MS/MS analysis. LC–MS/MS data were interpreted using the available *V*_*H*_-gene sequence information of IDC561. The detection of antibody heavy-chain complementarity determining region 3 (CDRH3)-derived peptides then allowed for the identification of HIV-1_Env_-specific antibody clonotypes that circulate in the plasma at a given timepoint^36,37^ (Fig. 6a).

**Extended Data Fig. 7:**
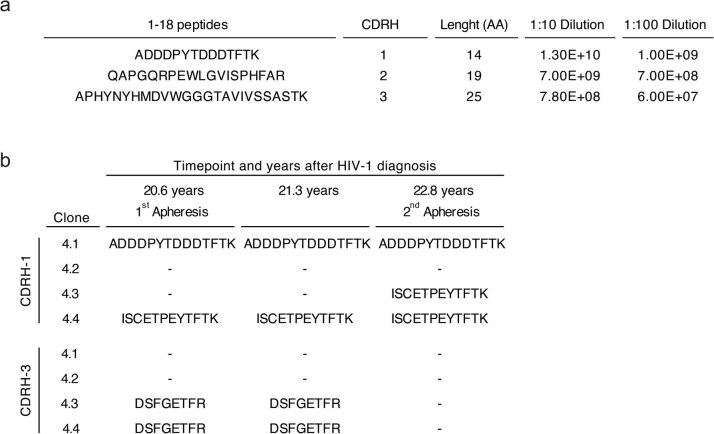
LC-MS/MS detection of clone 4 from individual IDC561. (**a**) Representative tryptic peptides of 1–18 mAbs detected by LC-MS/MS. Human plasma was mixed with purified monoclonal 1–18 antibody at 1:10 or 1:100, mass ratio. Anti- BG505_SOSIP.664_ antibodies were purified by affinity chromatography and tryptic peptides derived from CDR1, CDR2, CDR3 region of 1–18 mAbs were identified by LC-MS/MS. The unique CDRH1 peptide ADDDPYTDDDTFTK was also detected at a 1:1,000 dilution with a low XIC value. (**b**) Unique tryptic CDRH1 and CDRH3 peptides derived from Clone 4 detected by LC-MS/MS at different timepoints.

We identified circulating antibodies of 37 unique B cell clones at the first apheresis timepoint. Of those, 23 (62%) clones became undetectable at the time of the second apheresis. We also identified four newly elicited clones and clones with a much higher relative abundance. Of those the clones 2,671 and 14,955 accounted for more than 30% of the antibody response at this point (Fig. 6b and Supplementary Table 5). Most importantly, clone 4 bNAbs that collectively comprised 5.2% of the circulating antibody repertoire at the first apheresis decreased after ART initiation and their CDRH3 became undetectable at the second apheresis timepoint (Fig. 6b,c). However, while clone 4.1 CDRH3 peptides were not observed at the time of the second apheresis, we could detect trace levels of the exceptionally charged and thus readily ionizable clone 4.1 CDRH1 peptide ‘ADDDPYTDDDTFTK’ (unique to the 4.1 clonal lineage). The absolute concentration of clone 4.1 antibodies at different timepoints was estimated by spike-in of isobaric ADDDPYTDDDTFTK peptide based on the extracted-ion chromatography (XIC) peak area relative to a calibration curve. We determined that the concentration of the 4.1 clone was 0.82 µg ml^−1^ at the time of the first apheresis, decreasing to below the limit of quantitation in the second apheresis. We note that this estimate accounts for antibodies of the 4.1 lineage that comprises multiple antibodies with distinct CDRH3s (which, as mentioned above, were not detected at the second apheresis due to the lower ionization propensity of the CDRH3 peptides, Extended Data Fig. 7a,b). Overall, the lower fraction of clone 4 antibodies detected by serum proteomics is consistent with the weaker IgG neutralization (*r* = −0.97, *P* = 0.17; Fig. 6c). Interestingly, at both apheresis timepoints, the serological repertoire was dominated by IGHV1-69 antibodies that collectively comprised 35% of the entire BG505_SOSIP.664_ response at the first apheresis, rising to 50% at the second apheresis (Fig. 6d). In earlier studies, IGHV1-69 antibodies with comparable somatic hypermutation (SHM) levels to those detected in the serum of IDC561 had been isolated from HIV-1 elite neutralizers by peripheral B cell cloning, shown to bind to the CD4bs and to have appreciable although not exceptional HIV-1 neutralization breadth^38,39^.

We conclude that the declining plasma IgG-neutralizing activity after ART-mediated suppression of viremia correlates with a declining fraction of both bNAb-encoding memory B cells and circulating serum bNAbs.

## Discussion

For many infections and vaccines, dynamics of nAb responses are well-studied and knowledge about nAb durability can be used to estimate the duration of protection and to optimize vaccination regimens^40,41^. For instance, the longitudinal assessment of anti-SARS-CoV-2 nAb responses in convalescent individuals or vaccinated individuals helped to rapidly optimize COVID-19 vaccination strategies^42–44^. However, given that no effective HIV-1 vaccine is available yet and only a few people develop a potent neutralizing response during natural infection, knowledge about the durability of naturally developing anti-HIV-1 nAb responses is missing. To date, most studies assessed anti-HIV-1 serum neutralizing activity in people without ART and were thus only able to draw conclusions about the antibody responses during uncontrolled viral replication and therefore relatively high levels of antigen^20–22,26,27,45–48^.

Antigens from most viruses stimulate the development of binding antibodies and nAbs. However, in most of the few HIV-1-infected individuals that develop a potent neutralizing serum response, a year’s long co-evolution between virus and antibodies was needed to develop broad and potent neutralizing serum responses^5,16,18^. In most individuals studied, this co-evolution was facilitated by long periods without ART resulting in high viral loads/antigen levels and future vaccine strategies may need to mimic this co-evolution^11^. To date, several promising candidates have entered clinical development, but there is still no vaccine available that elicits broad and potent nAbs in humans. If future vaccines achieve to elicit potent bNAbs, the longevity of such antibodies will also be an important criterion for vaccine strategies to offer long-term protection. However, many potent HIV-1 nAbs have high rates of somatic hypermutation including insertions and deletions that make their induction difficult. Furthermore, it is discussed that these features may affect their durability after vaccination or after antigen levels drop, that is, after ART initiation^11,49,50^. Thus, although several promising vaccine candidates are under development, it remains unclear how long vaccine-induced nAb responses would last and offer protection^12,13,51^. Until such nAb-inducing vaccines are available, the dynamics and durability of neutralizing serum responses can only be described in HIV-1-infected individuals who developed a neutralizing serum response upon natural infection. Moreover, by analyzing these neutralizing serum responses in association with the viral load, the impact of antigen levels on nAb durability can be evaluated. To this end, we assessed IgG neutralization in a large HIV-1-infected cohort and longitudinally described the nAb response in neutralizers over time in response to different viral dynamics.

In our cohort of HIV-1-infected persons from various countries, we identified several factors as independent predictors for better IgG neutralization. Most of these factors reflected the ART intake, such as the CD4^+^ T cell count and the current and former treatment status. Although the impact of longer and higher viral loads on nAb responses was similarly reported in previous studies, it has not been shown in a large cohort that ART intake negatively influences IgG neutralization^20–22,24–28^. However, as HIV-1 neutralizing serum activity does not protect from HIV-1 disease progression, ART intake is crucial for HIV-1-infected individuals as ART reduces mortality and morbidity, enhances quality of life, and prevents HIV transmission by suppressing viral replication^52^.

To further investigate the changes in the nAb response over time in response to different levels of viral replication, reflecting different antigen levels, we conducted a longitudinal study of individuals with potent HIV-1-neutralizing IgG activity. We found nAb half-lives in individuals with no- or low-level viremia of 9.3 and 16.9 years, respectively. With 4.0 years, half-life was considerably shorter in individuals who initiated ART and thus experienced a suppression of their viremia during the follow-up period. Thus, neutralizing serum responses are overall relatively stable over time even without large amounts of stimulating antigen. The half-lives of neutralizing antibody titers that we determined were comparable to the half-life of non-neutralizing anti-HIV-1_Env_ antibody responses in HIV-1-positive persons after the initiation of ART^53,54^ and considerably longer than the 1–2 month long half-lifes of non-neutralizing antibody responses after immunization with experimental HIV-1 vaccine candidates^55,56^. If effective nAb-inducing vaccines are available in the future, our data indicate that nAbs can be rather long-lived even if the vast majority of the stimulating antigen vanishes over time.

Dynamics of binding- and neutralizing serum responses have previously been described for other infectious diseases such as COVID-19 (refs. ^43,44,57^). However, in individuals that have a neutralizing serum response against a specific pathogen, it is difficult to dissect binding (including non-nAbs) from neutralizing antibody responses by serological assays. For an accurate assessment of the dynamics of neutralizing and non-neutralizing antibodies, investigating single antibody and B cell clones can be informative as we showed in our analysis of individual IDC561. We found the weaker neutralizing response after ART initiation to be associated with reduced levels of circulating pre-existing bNAbs and a decrease of bNAb-encoding B cells among HIV-1_Env_-specific B cells and among the overall memory B cell repertoire, respectively. Interestingly, we also identified several new or considerably more expanded memory B cell and anti-HIV-1_Env_ circulating antibody clones after ART initiation. This indicates a continuous evolution of HIV-1-directed B cells even during suppressed viral replication as shown before in elite controllers^58,59^. However, low levels of circulating plasma virus or ongoing HIV-1 replication in specific compartments during ART might contribute to this ongoing evolution and might also affect long nAb durability in individuals on ART described herein^60^. Of note, although the fraction of serum bNAbs and bNAb-encoding B cells declined, the newly identified bNAbs of clones 4.1 and 4.2 did not significantly change in their *V*_*H*_-gene sequence and maintained their potent and broad-neutralizing activity after IDC561’s ART initiation. However, it needs to be considered that these findings are derived from only one individual and further individuals with neutralizing serum responses would need to be studied to draw firm conclusions. It is also possible that not all relevant HIV-1-reactive B cells of individual IDC561 have been captured by the sorting strategies applied. Another limitation of our study is the measurement and assessment of the antigen levels in the participants. While viral loads can inform about the antigen levels in serum/plasma, we have not collected further samples to ensure that these patients had also lower antigen levels in lymphatic organs such as lymph nodes or the gut.

In conclusion, our analyses show that anti-HIV-1 IgG-neutralizing activity is associated with viral replication and thus weakens after ART initiation. However, nAb responses are overall relatively stable with half-lives of several years. Thus, our study indicates that future active vaccine strategies that are able to elicit protective titers of nAbs in humans could provide long-term protection from HIV-1 infections.

## Methods

### Human participants

Blood and leukapheresis samples were obtained under research protocols that were approved by the Institutional Review Boards and ethics committees of the respective study site. All participants gave written informed consent, according to CARE (case report) guidelines and in compliance with the Declaration of Helsinki principles. Participants received a small compensation for study participation at some sites. HIV-1-infected individuals were recruited at private practices and/or hospitals in Germany (Cologne, Essen and Frankfurt), Cameroon (Yaoundé), Nepal (Kathmandu) and Tanzania (Mbeya). Overall, sex/gender was not considered in the study design, and we have only collected data on assigned, not self-reported sex. Data on gender were not collected. In total, 2,392 individuals were screened for their neutralizing activity. Of those, 38 individuals were excluded from further analysis due to increased unspecific neutralizing background activity as described below. Among the final dataset of 2,354 participants, there were 737 female and 1,542 male patients. For 75 individuals, no information on their sex was available. Median age of the whole cohort was 41.8 years. Individual IDC561 was already described in detail in ref. ^33^, and data from this first apheresis timepoint were used for comparison with new timepoints. Viral load measurements of the longitudinal cohort of 71 individuals were performed at each respective study site by commercially available assays and had a limit of detection at 20 copies per ml. Mean viral loads, as depicted in Fig. 4c, were calculated by the geometric mean viral load of all individuals in predefined time frames.

### Cell lines

All cells were maintained at 37 °C and 5% CO_2_. HEK293T cells (American Type Culture Collection) in Dulbecco’s modified Eagle medium (DMEM; Thermo Fisher Scientific) supplemented with 10% FBS (Sigma-Aldrich), 1 mM sodium pyruvate, 2 mM l-glutamine and 1× antibiotic–antimycotic (all from Thermo Fisher Scientific). TZM-bl cells were maintained in DMEM supplemented with 10% FBS, 1 mM sodium pyruvate, 2 mM l-glutamine, 50 mg ml^−1^ gentamicin (Merck) and 25 mM HEPES (Millipore). SupT1.CCR5 cells were maintained in R10 (Roswell Park Memorial Institute (RPMI, GE Life Sciences) supplemented with 10% FBS, 2 mM l-glutamine and 100 U ml^−1^ of penicillin and streptomycin). The 293-6E cells (National Research Council of Canada) were maintained in FreeStyle Expression Medium (Thermo Fisher Scientific). The sex of HEK293T, TZM-bl and 293-6E is female, whereas the sex of SUPT1.CCR5 is male.

### Clinical samples

Density-gradient centrifugation was used to isolate peripheral blood mononuclear cells (PBMCs) from full blood or leukapheresis samples. PBMCs were stored at −150 °C in 90% FBS and 10% Dimethyl sulfoxide (DMSO, Sigma-Aldrich) until further use. Plasma and serum samples were stored at −80 °C until further use.

### Serum and plasma IgG isolation for neutralization and binding assays

Serum or plasma samples were heat-inactivated at 56 °C for 40 min. Following, serum/plasma was incubated with Protein G (Protein G Sepharose 4 Fast Flow; Merck GE17-0618) in PBS at 4 °C overnight. For our screening, we used 500 µl of plasma/serum per individual that was incubated with 250 µl of Protein G. On the next day, Protein G, which now carries the participant’s IgGs, was added on chromatography columns and was washed 3× with 1× PBS. Following, IgGs were eluted with 0.1 M glycine (pH = 3) and the final solution was immediately buffered using 1 M Tris buffer (pH = 8). Finally, the buffer was exchanged to 1× PBS using Amicon Ultra spin tubes (Amicon Ultra-15 Centrifugal Filter Units; Merck, UFC9030) until the original buffer was sufficiently replaced (at least 1:200) and less than 0.5% of Tris/Glycin remained. Final concentration was measured using a NanoDrop (VWR). Purified IgGs were stored at 4 °C or −80 °C until further use.

### Pseudovirus production

Pseudoviruses (12-strain global screening panel and f61 fingerprinting panel) were used for neutralization assays. Pseudoviruses were produced in HEK293T cells after cotransfection of the respective HIV-1_env_-plasmids with the pSG3Δenv plasmid as described previously^30,61–64^.

### Neutralization assays

Neutralization assays were performed as described previously^63^. In brief, pseudoviruses and antibodies or purified participants’ IgG were mixed and co-incubated at 37 °C for 1 h in a 96-well plate, followed by the addition of TZM-bl cells. Following a 2-d incubation period, a luciferase-containing lysing reagent was added and luminescence was determined using a luminometer. After subtracting background relative luminescence units of noninfected TZM-bl cells, the percent inhibition or 50% IC_50_s were determined. For our large screening, isolated participants’ IgGs were tested in a single well per virus at a single concentration of 300 µg ml^−1^. Duplicates were performed for 28,362 of 30,602 (93%) tests resulting in a median s.d. between all duplicates of 5.2% inhibition (interquartile range: 2.3–9.2%). Mean result of the duplicates was used for further analysis. To categorize individuals, each participant’s IgG potency against each strain received a score between 0 and 3 points (neutralization at 300 µg ml^−1^: <20% neutralization = 0 points; 20–50% = 1 point, 50–80% = 2 points, >80% = 3 points). The sum of this score against all 12 pseudoviruses then was used to stratify individuals according to their neutralization activity in elite neutralizer (23–36 points), broad neutralizer (15–22 points), cross-neutralizer (8–14 points) or non-neutralizer (0–7 points). All IgGs were tested for unspecific activity against murine leukemia virus (MuLV)-pseudotyped viruses, and if unspecific activity was observed (25% inhibition against MuLV), individuals were excluded from further analysis (*n* = 38).

### Generation of BG505 pseudovirus mutants

Point mutations were introduced into a BG505_T332N_ envelope expression plasmid using the Q5 Site-Directed Mutagenesis Kit (New England Biolabs). Pseudoviruses were produced as described above.

### DMS

HIV DMS was performed as described previously^32,65^. Briefly, HIV-1_BG505_ mutant virus libraries^66^ were incubated with concentrations of each sera in the IC_90–99_ range along with a mock incubation for each library. After incubation, viruses were used to infect SupT1.CCR5 cells. Twelve hours after infection, nonintegrated viral cDNA genomes were isolated using a miniprep, as described previously^67,68^. Viral cDNA genomes were then sequenced using barcoded subamplicon sequencing. These data were analyzed using dms_tools2 (ref. ^69^) to calculate the differential selection for each mutation under each serum selection, or the logarithm of that mutation’s enrichment in the serum selection relative to the mock selection control. See https://github.com/jbloomlab/HIV_Broad_Human_Sera_MAP for the code used for this analysis. For IDC561, IDC508, IDF033 and IDC513, DMS was performed using lentivirus-based DMS^31,68^. DMS results of individuals IDC561, IDC508, IDF033 and IDC513 have previously been shown in ref. ^31^. The lentivirus DMS escape map for IDC508 from ref. ^31^ is displayed in Extended Data Fig. 2c as a single epitope for simplicity. See https://dms-vep.github.io/HIV_Envelope_BF520_DMS_CD4bs_sera/ for the code used for this analysis and interactive plots of the data.

### Area under the curve calculation of participants’ IgG neutralization

We used the area under the neutralization curve (AUC) to compare the neutralizing activity of an individual over time in the cohort of 71 neutralizers as shown previously^70,71^. While neutralization curves were generated for all 71 neutralizers against the global panel strains, the usage of the AUC enables the inclusion of samples with low-level neutralization, where the IC_50_ was not reached due to lower active IgGs. The AUC was computed as the area under the neutralization titration curve with the R package flux (version 0.2.1.), where the logarithmized antibody concentrations are on the *x* axis and the measured neutralization activity on the *y* axis. Based on replicative measurements and the neutralization activity against the control virus MuLV, the AUC is corrected for variance between experiments and background noise of the assay.

To quantify the cluster-specific decline in neutralizing serum IgG activity over time (Fig. 4d and Extended Data Fig. 3d), linear mixed effect models (R-function lme4::lmer) with random slope and specific intercepts for each individual were applied to model the mean AUC as a function of the number of days passed since the baseline visit^72^. Confidence bands were computed using R-function ggeffects::ggpredict^73^. Half-life estimates of neutralizing serum IgG activity were obtained by fitting separate linear mixed models using a log_2_-transformation of the mean AUC as response and computing the negative inverse of the resulting common slope regression coefficient.

### Clustering of individuals according to viral load

All individuals that had a fully suppressed plasma viral load (viral loads below the limit of detection) at all measurements between the two sampling timepoints were assigned to the ‘no viremia’ group. For each of the remaining individuals, a feature vector containing the first, the last, the mean and the median of the available viral loads between the sampling timepoints was created to compare persons with a different number of measurements. *K*-means clustering was applied in the resulting four-dimensional feature space. Different values for the number of clusters *K* were tested, with *K* = 3 being optimal according to the change in within-cluster dissimilarity.

### Single-cell sort

The Pan B Cell Isolation Kit, B Cell Isolation Kit II or IgG^+^ Memory B Cell Isolation Kit (Miltenyi Biotec) was used for the isolation of B cells from PBMCs. Following, B cells were labeled with anti-human CD19-AF700 (1:20, BD Biosciences), anti-human IgG-APC (1:20, BD Biosciences), DAPI (1:100, Thermo Fisher Scientific) and the HIV-1 Env baits BG505_SOSIP.664_-GFP (1.5 µg/100 µl) or biotinylated YU2_gp140_ (labeled with Streptavidin-Phycoerythrin (PE, BD Biosciences), 1.5 µg/100 µl) for 30 min on ice. Env-reactive CD19^+^IgG^+^DAPI^−^ single cells were sorted into 96-well plates containing 4 µl of lysis buffer (0.5× PBS, 10 mM DTT (Thermo Fisher Scientific), 2 U µl^−1^ RNasin (Promega) and 1 U µl^−1^ RNaseOUT (Thermo Fisher Scientific)) per well using a BD FACSAria Fusion with BD FACSDiva software (version 8.0). Plates were stored at −80 °C until further use. Frequency of BG505_SOSIP.664_- (top) or YU2_gp140_-reactive IgG^+^ B cells from the parental gate was calculated using FlowJo-Software (version 10.5.0). The same software was used to illustrate the gating strategy of each respective sort.

### Single-cell cDNA synthesis, PCRs, antibody sequence and clonality analysis

Single-cell cDNA synthesis and following amplification of antibody sequences were performed as described previously^33^. In short, cDNA synthesis was performed using Superscript IV (200 U µl^−1^; Thermo Fisher Scientific) according to the manufacturer’s protocol and additionally added RNAse inhibitors. Antibody sequences for single-cell analysis were amplified by semi-nested PCRs using Platinum Taq DNA Polymerase or Platinum Taq Green Hot Start DNA Polymerase (Thermo Fisher Scientific) and previously described primers and their respective PCR settings^74–76^. Second-round PCR products were sequenced by Sanger sequencing and used for further sequence analyses as described previously^33^. Sequences were annotated with IgBLAST^77^. Clonal analysis was performed on a dataset of combined single B cell sequences from both apheresis timepoints as described previously^33^. All clones were cross-validated by the investigators taking shared mutations and light chain information into account.

The maximum-likelihood phylogenetic tree was generated using nucleotide sequences of heavy-chain V genes (FWRH1–FWRH3) and of the IGHV1-46*01 *Homo sapiens* allele (GenBank X92343.1). All sequences were aligned using ClustalW (Geneious R10; cost matrix: IUB; gap open cost: 15; gap extend cost: 6.66), and the maximum-likelihood phylogenetic tree was calculated using PhyML with 1,000 bootstrap replicates as described in ref. ^78^ (substitution model: general time reversible; Geneious R10) (ref. ^78^). The best-scoring tree was then rooted to IGHV1-46*01.

### Antibody cloning and production

Antibody cloning and production were performed as described previously^33^. In short, PCR products of single B cells were amplified with specific forward primers and reverse primers providing overhangs for subsequent sequence and ligation-independent cloning (SLIC)^74,76^. PCR products or synthesized DNA fragments were cloned into human antibody expression vectors (IgG1, kappa or lambda chain) by SLIC assembly as described previously^79^. Antibodies were produced in 293-6E cells (National Research Council Canada). Five to seven days after transfection with polyethylenimine (PEI), monoclonal antibodies were isolated from supernatants using Protein G as described above and stored at 4 °C until further use.

### Neutralization fingerprinting panel-based antibody epitope prediction

Computational epitope prediction of serum IgG-neutralizing activity was conducted as described previously^61,80^. In brief, the neutralizing IC_50_s of the respective participant’s IgGs were determined against the 20 pseudovirus f61 panel. Following, the similarity of the fingerprint of the tested participants’ IgGs is compared to the fingerprint of ten classes of reference-bNAbs, grouped by their specific epitope; for each class, shown is a prototypic bNAb member. The prevalence of these reference antibody epitopes in a respective participant’s IgG is computationally predicted and assigned a delineation score between 0 (low) and 1 (high). The scores as shown in Fig. 2a represent the percentage ((initial delineation score) × 100).

### HIV-1 Env ELISAs

HIV-1 Env antigens at 2 µg ml^−1^ were coated on high-binding ELISA plates (Corning) in PBS overnight at 4 °C. Subsequently, wells were blocked for 60 min at 37 °C with 3% BSA (Sigma-Aldrich) in PBS. Antibodies or participants’ IgGs to be tested were diluted in PBS and incubated for 60 min at room temperature after addition to the coated plates. Following, horseradish peroxidase-conjugated anti-human IgG (Jackson ImmunoResearch) diluted 1:1,000 in 3% BSA in PBS was added for 60 min at room temperature. After the addition of 2,2′-azino-di-(3-ethylbenzthiazoline sulfonic acid (ABTS) solution (Thermo Fisher Scientific), absorbance was determined on a microplate reader (Tecan). Between each step, plates were washed with 0.05% Tween 20 (Carl Roth) in PBS.

### Unbiased B cell repertoire analyses

We performed an unbiased B cell repertoire analysis at the timepoint of individual IDC561 as described previously^33^. In brief, B cells were isolated from PBMCs using CD19 microbeads (Miltenyi Biotec) and stained with DAPI (1:100; Thermo Fisher Scientific), CD20-AF700 (1:80), IgG-APC (1:20), IgD-Pe-Cy7 (1:20), IgM-FITC (1:5) and CD27-PE (1:40; all BD Biosciences). In total, 450,000 and 400,000 CD20^+^IgG^+^IgM^−^IgD^−^CD27^−^ B cells of the first and second apheresis of IDC561, respectively, were sorted into FBS (Sigma-Aldrich) using a BD FACSAria Fusion. In total, 250,000 and 200,000 cells of the first apheresis sample and 200,000 and 200,000 cells of the second apheresis samples were sorted in separate experiments. These four sorting samples were handled separately in the downstream assays described here. Following, RNA of sorted B cells was isolated with the RNeasy Micro Kit (Qiagen). We then generated cDNA according to the SMARTer RACE 5′/3′ manual using the SMARTScribe Reverse Transcriptase (Takara) with a template-switch oligo including an 18-nucleotide unique molecular identifier. Heavy-chain variable regions were amplified with an IgG-specific nested PCR, and amplicons were used for library preparation and MiSeq 2 × 300 bp sequencing (Illumina). Raw NGS reads were preprocessed and assembled to final sequences as described previously^81^. Clone 4 members were identified from NGS data by their CDRH3. To this end, a database of known clone 4 CDRH3s (as published in ref. ^33^) was set up and the B cell repertoire data of first and second apheresis were searched for IGHV1-46 gene sequences that matched CDRH3s in the clone 4 CDRH3 dataset. For the analysis of the naïve B cell repertoire, CD20^+^IgM^+^IgD^+^CD27^−^IgG^−^ cells were sorted.

### IgG purification, antigen enrichment of F(ab′)2 and MS sample preparation for Ig-seq

IDC561 serum (1 mg) was diluted 1:1 with Dulbecco’s PBS (DPBS; Gibco, Thermo Fisher Scientific), and IgG was isolated by passing through column three times with 1.5 ml protein G agarose (Pierce, Thermo Fisher Scientific). Then, the column was washed with DPBS and eluted with glycine–HCl (pH = 2.7), followed by immediate neutralization with 1 M Tris–HCl (pH = 8.0). To concentrate IgG and remove salts, buffer was exchanged to PBS using a 15 ml 10 K molecular weight cut-off (MWCO) tube (Thermo Fisher Scientific). To generate F(ab′)2, purified IgG was incubated with IdeS (1:50 mass ratio, IdeS:IgG) for 2 h at 37 °C.

BG505_SOSIP.664_ was immobilized on the affinity column for antigen-specific F(ab′)2 enrichment as described previously^34,37,82^. Briefly, 0.5 ml of BG505_SOSIP.664_ (1 mg ml^−1^ in PBS) was added to 50 mg of NHS-activated agarose resin (Pierce, Thermo Fisher Scientific) and rotated overnight at 4 °C. BG505_SOSIP.664_-conjugated NHS resin was washed twice with DPBS and incubated with 1 methanolamine (pH = 8.2) for 30 min to quench NHS resin. BG505_SOSIP.664_-conjugated NHS resin was then transferred to a spin column (Pierce, Thermo Fisher Scientific) and washed 12 times with 1 column volume (400 µl) DPBS, spinning down the resin 1000 g for 30 s for each round. Then, SOSIP-conjugated resin was incubated with prepared IdeS-IgG for 1 h at room temperature and flow through (unbound IgG) was collected. The column was then washed with DPBS 11 times and BG505_SOSIP.664_-specific F(ab′)2 was eluted with 1% formic acid (400 µl fractions). Elution fractions were dried in speed vacuum to remove formic acid and resuspended with 25 µl of LC–MS graded water and adjusted to neutral pH. Flow-through and elution samples were denatured and reduced in 50% (vol/vol) 2,2,2-trifluoroethanol with 5 mM Tris(2-carboxyethyl)phosphine hydrochloride (TCEP) at 55 °C for 1 h and alkylated with 16.5 mM iodoacetamide (MilliporeSigma) for 30 min at room temperature in the dark. Samples were then digested with trypsin (1:30 trypsin/protein) overnight at 37 °C. Formic acid was added (1%) to quench the tryptic digestion, and samples were dried to 20 µl under speed vacuum. For clean-up, tryptic peptides were loaded onto a C18 HyperSep SpinTip (Thermo Fisher Scientific), washed three times with 0.1% formic acid and eluted with releasing buffer (60% acetonitrile and 0.1% formic acid). The tryptic eluates were dried under speed vacuum and resuspended to 50 µl (5% acetonitrile and 0.1% formic acid) for LC–MS/MS analysis.

### Bioinformatic analysis of Ig-seq

Raw IGHV and immunoglobulin light chain variable (IGLV) sequences derived from NGS and single B cell-sequencing data were annotated to human V, D and J germline reference sequences using MiXCR software^83^. Then, the annotated IGHV reads were clustered into lineages (that is, clonotypes) based on a single-linkage hierarchical clustering algorithm, with a requirement of ≥90% identity within the CDRH3 amino acid sequences (measured by Levenshtein edit distance) as reported previously^34,82^. The IGHV and IGLV sequences that were identified with ≥2 reads were combined and used as a personalized search database for database-driven high-resolution LC–MS/MS searches. These were performed using SEQUEST and Percolator in Thermo Proteome Discoverer 1.4 as described^34,37,82^. Briefly, the abundance of each peptide was calculated from the XIC peak area generated by the Precursor Ions Area Detector node in Proteome Discoverer (Thermo Fisher Scientific). This allowed us to measure the abundance for each lineage by summing the XIC area of high-confidence CDRH3 peptide spectra matches on the condition that such CDRH3 peptide sequences are mapped to a single lineage without any ambiguity. BG505_SOSIP.664_-specific IgG was defined as antibody lineages with ≥5-fold higher signal (XIC) in the elution as compared to the flow through.

### Quantification and statistical analysis

To estimate the RRR of neutralizing activity, uninomial and multinomial logit models using mlogit (Stata Statistical Software: Release 17) were performed. As a dependent variable, the neutralizing activity was defined with the non-neutralizers as the base value. The mlogit test using the Wald parameter was applied to assess the likelihood ratio for each variable included in the model. The RRR and 95% CI were reported to show the direction and strength of the association.

Differences in epitope mapping distributions between top-, mid- and low-neutralizers were compared by using the Chi-square test that was also used to compare differences in clone proportions between first and second apheresis sorts of IDC561. Two-sided Student’s *t*-test was used for comparison of BG505- and YU2-reactive B cell frequencies of the different timepoints.

EC_50_ values as determined by ELISA were calculated using the fitting settings for sigmoidal curves, 4PL in GraphPad, Version 9.3.1. IC_50_ values of neutralization activity were determined using a Microsoft Excel macro as described previously^84^.

A one-way analysis of variance (ANOVA) was calculated using SPSS Statistics (version 23; IBM). One-way ANOVA with Tukey’s correction for multiple comparisons, Pearson’s correlation coefficient, Wilcoxon matched-pairs signed rank test and two-sided students *t*-test were calculated using Prism (GraphPad, version 9.3.1).

All *P* values of <0.05 were considered statistically significant.

### Reporting summary

Further information on research design is available in the Nature Portfolio Reporting Summary linked to this article.

## Online content

Any methods, additional references, Nature Portfolio reporting summaries, source data, extended data, supplementary information, acknowledgements, peer review information; details of author contributions and competing interests; and statements of data and code availability are available at 10.1038/s41591-023-02582-3.

### Supplementary information

Supplementary Information Supplementary Tables 1–5.

Reporting Summary

## Data Availability

Aggregated clinical data are available upon request to the corresponding author (F.K.) provided that there is no reasonable risk of de-anonymizing study participants. Individual patient data cannot be shared due to privacy restrictions. The sequence data of the NGS-based unbiased B cell repertoire analysis of IDC561 will be available upon request and after completion of a Data Transfer Agreement between the University Hospital Cologne and the requesting institution/researcher (for requests contact: florian.klein@uk-koeln.de). Nucleotide sequences of all generated antibodies were deposited at GenBank under the accession numbers OR498214–OR498255.

## Code availability

See https://github.com/jbloomlab/HIV_Broad_Human_Sera_MAP for the code used for analysis of HIV DMS data and https://dms-vep.github.io/HIV_Envelope_BF520_DMS_CD4bs_sera/ for the code used for analysis and interactive plots of the lentivirus DMS data.
