# Supplementary material for: Dynamics and durability of HIV-1 neutralization are determined by viral replication
Source: Nat Med. 2023 Nov 13;29(11):2763–74. doi: 10.1038/s41591-023-02582-3 (PMC10667105; doi:10.1038/s41591-023-02582-3)
Supplement: Supplementary file 1 — Supplementary Tables 1–5. [file 41591_2023_2582_MOESM1_ESM.pdf]

nature medicine

Article

https://doi.org/10.1038/s41591-023-02582-3

# Dynamics and durability of HIV-1 neutralization are determined by viral replication

In the format provided by the authors and unedited

**Supplementary Table 1.** Global panel, f61 panel and epitope mapping results of Mid and Top Neutralizers (as indicated in Figure 2)

Left panel: Screening against the global panel. Second column indicates ranking of each individual (among all 2354 screened individuals) and its respective cohort.
Middle panel: Neutralization activity against the f61 fingerprint panel.
Right panel: Epitope mapping (calculated from f61 panel neutralization results)

## Global panel screening

| | Rank of patient | Cohort | 398F1 (A) | 246F3 (AC) | CNE55 (AE) | CNE8 (AE) | X2278 (B) | Tro11 (B) | BJOX2000 (BC) | CH119 (BC) | CE1176 (C) | CE0217 (C) | 25710 (C) | X1632 (G) | Mean Neutr. (%) | Breadth (%) |
|---|---|---|---|---|---|---|---|---|---|---|---|---|---|---|---|---|
| Mid Neutralizer | 26 | G | 93 | 93 | 84 | 73 | 98 | 98 | 86 | 99 | 60 | 40 | 83 | 78 | 82 | 92 |
| Mid Neutralizer | 27 | N | 85 | 85 | 90 | 72 | 91 | 84 | 89 | 75 | 46 | 80 | 93 | 94 | 82 | 92 |
| Mid Neutralizer | 28 | T | 91 | 74 | 100 | 47 | 71 | 89 | 85 | 86 | 85 | 61 | 95 | 92 | 81 | 92 |
| Mid Neutralizer | 29 | T | 95 | 96 | 75 | 45 | 88 | 58 | 88 | 66 | 97 | 92 | 76 | 98 | 81 | 92 |
| Mid Neutralizer | 30 | N | 83 | 89 | 70 | 66 | 83 | 64 | 87 | 70 | 95 | 98 | 82 | 80 | 81 | 100 |
| Mid Neutralizer | 31 | G | 72 | 89 | 78 | 94 | 89 | 82 | 83 | 68 | 79 | 74 | 95 | 64 | 81 | 100 |
| Mid Neutralizer | 33 | T | 81 | 89 | 82 | 77 | 75 | 82 | 93 | 88 | 87 | 37 | 91 | 78 | 80 | 92 |
| Mid Neutralizer | 34 | T | 99 | 94 | 89 | 79 | 100 | 25 | 91 | 41 | 73 | 97 | 88 | 84 | 80 | 83 |
| Mid Neutralizer | 35 | T | 89 | 74 | 52 | 83 | 89 | 87 | 52 | 88 | 92 | 68 | 94 | 90 | 80 | 100 |
| Mid Neutralizer | 36 | T | 86 | 86 | 91 | 84 | 96 | 86 | 53 | 74 | 56 | 92 | 81 | 68 | 79 | 100 |
| Mid Neutralizer | 37 | C | 94 | 84 | 57 | 61 | 87 | 72 | 89 | 69 | 99 | 84 | 96 | 60 | 79 | 100 |
| Mid Neutralizer | 38 | G | 81 | 91 | 70 | 69 | 96 | 69 | 83 | 96 | 75 | 48 | 84 | 88 | 79 | 92 |
| Mid Neutralizer | 39 | T | 99 | 94 | 26 | 46 | 44 | 93 | 99 | 95 | ## | 96 | 98 | 54 | 79 | 75 |
| Mid Neutralizer | 40 | N | 73 | 72 | 92 | 86 | 86 | 81 | 69 | 94 | 70 | 74 | 92 | 56 | 79 | 100 |
| Mid Neutralizer | 42 | T | 88 | 95 | 67 | 89 | 46 | 21 | 99 | 95 | 82 | 95 | 81 | 75 | 78 | 83 |
| Mid Neutralizer | 45 | T | 81 | 91 | 62 | 79 | 70 | 94 | 57 | 88 | 73 | 64 | 92 | 76 | 77 | 100 |
| Mid Neutralizer | 46 | T | 98 | 90 | 70 | 75 | 86 | 89 | 76 | 57 | 96 | 44 | 96 | 49 | 77 | 83 |
| Low Neutralizer | 47 | T | 85 | 79 | 65 | 52 | 70 | 83 | 85 | 93 | 71 | 76 | 87 | 74 | 77 | 100 |
| Low Neutralizer | 53 | T | 97 | 100 | 86 | 99 | 95 | 71 | 53 | 43 | 31 | 91 | 85 | 62 | 76 | 83 |
| Low Neutralizer | 58 | G | 93 | 93 | 57 | 56 | 46 | 94 | 41 | 97 | 98 | 87 | 96 | 51 | 76 | 83 |
| Low Neutralizer | 71 | G | 91 | 79 | 59 | 58 | 64 | 35 | 80 | 92 | 88 | 61 | 89 | 86 | 73 | 92 |
| Low Neutralizer | 76 | G | 85 | 91 | 99 | 92 | 54 | 77 | 74 | 49 | 28 | 39 | 97 | 81 | 72 | 75 |
| Low Neutralizer | 77 | G | 58 | 90 | ## | 95 | 59 | 70 | 65 | 76 | 36 | 28 | 97 | 93 | 72 | 83 |
| Low Neutralizer | 82 | G | 95 | 93 | 28 | 20 | 94 | 65 | 90 | 74 | 92 | 64 | 95 | 50 | 72 | 83 |
| Low Neutralizer | 84 | G | 81 | 86 | 86 | 61 | 97 | 81 | 72 | 82 | 81 | 14 | 80 | 38 | 72 | 83 |
| Low Neutralizer | 87 | G | 94 | 86 | 95 | 75 | 100 | 69 | 64 | 39 | 66 | 0 | 68 | 95 | 71 | 83 |
| Low Neutralizer | 90 | G | 68 | 60 | 73 | 79 | 93 | 88 | 62 | 72 | 59 | 32 | 83 | 71 | 70 | 92 |
| Low Neutralizer | 101 | G | 97 | 90 | 50 | 52 | 65 | 69 | 57 | 64 | 78 | 49 | 89 | 62 | 69 | 83 |
| Low Neutralizer | 104 | G | 97 | 59 | 37 | 59 | 32 | 93 | 89 | 89 | 93 | 29 | 86 | 59 | 68 | 75 |
| Low Neutralizer | 111 | G | 83 | 83 | 72 | 74 | 70 | 76 | 49 | 55 | 71 | 27 | 90 | 58 | 68 | 83 |
| Low Neutralizer | 139 | G | 95 | 94 | 9 | 48 | 48 | 87 | 71 | 28 | 96 | 67 | 81 | 43 | 64 | 58 |
| Low Neutralizer | 173 | G | 99 | 36 | 0 | 0 | 77 | 91 | 98 | 91 | 88 | 57 | 75 | 14 | 61 | 67 |
| Low Neutralizer | 189 | G | 97 | 55 | 70 | 71 | 31 | 33 | 54 | 41 | 77 | 27 | 85 | 61 | 59 | 67 |
| Low Neutralizer | 294 | G | 36 | 99 | 90 | 96 | 40 | 29 | 22 | 36 | 23 | 30 | 64 | 37 | 50 | 33 |

Germany (G) | Tanzania (T) | Nepal (N) | Cameroon (C)

% Neutralization ( at 300 µg/ml): >90 | 90-80 | 80-70 | 70-50 | <50

## f61 panel neutralization

| | Rank of patient | Cohort | KER2008.12 (A) | Q259.d2.17 (A) | Q461.e2 (AD) | 620345.c1 (AE) | CNE5 (AE) | CNE55 (AE) | M02138 (AE) | TH976.17 (AE) | 242-14 (AE) | 7165,18 (B) | YU2.DG (B) | CH038.12 (BC) | CH070.1 (BC) | 0013095-2.11 (C) | 001428-2.42 (C) | 26191-2.48 (C) | 3168.v4.c10 (C) | ZM135M.PL10a (C) | 231965.c1 (D) | 247-23 (D) | Geo. Mean IC50 (µg/ml) | Breadth (%) |
|---|---|---|---|---|---|---|---|---|---|---|---|---|---|---|---|---|---|---|---|---|---|---|---|---|
| Mid Neutralizer | 26 | G | 98 | 140 | >500 | 271 | 54 | 150 | 235 | 122 | 201 | 453 | 110 | >500 | 122 | 215 | 119 | 303 | 380 | 187 | >500 | 260 | 176 | 85 |
| Mid Neutralizer | 27 | N | 195 | 48 | 139 | 85 | 266 | 142 | 171 | 129 | 164 | >500 | 129 | 486 | >500 | 63 | 43 | 125 | 261 | 317 | 328 | 142 | 149 | 90 |
| Mid Neutralizer | 28 | T | >500 | >500 | >500 | >500 | >500 | 21 | 118 | >500 | 97 | >500 | 300 | 27 | 189 | 111 | 219 | 241 | >500 | 237 | >500 | >500 | 117 | 50 |
| Mid Neutralizer | 29 | T | >500 | 79 | >500 | >500 | 65 | 192 | 126 | 235 | 123 | >500 | >500 | 174 | 39 | 75 | 56 | 241 | >500 | 202 | >500 | 104 | 113 | 65 |
| Mid Neutralizer | 30 | N | 141 | 108 | >500 | 370 | 119 | >500 | 392 | 125 | >500 | >500 | >500 | 327 | 37 | 125 | 57 | 273 | 492 | 210 | >500 | >500 | 167 | 65 |
| Mid Neutralizer | 31 | G | >500 | >500 | 185 | >500 | 397 | 359 | 85 | 39 | 100 | >500 | 256 | 431 | >500 | 80 | 65 | 78 | >500 | 106 | >500 | >500 | 137 | 60 |
| Mid Neutralizer | 33 | T | 128 | 122 | 114 | 97 | 84 | 195 | 107 | 27 | 68 | >500 | >500 | >500 | >500 | 215 | 160 | 258 | 253 | 70 | 194 | 368 | 129 | 80 |
| Mid Neutralizer | 34 | T | 72 | 231 | 316 | >500 | 46 | 150 | 219 | >500 | 52 | 378 | 139 | >500 | 34 | 61 | 59 | 245 | 110 | >500 | >500 | >500 | 115 | 70 |
| Mid Neutralizer | 35 | T | 36 | 67 | 385 | 47 | 40 | 360 | 18 | 43 | 43 | 260 | 186 | 77 | 116 | 77 | 19 | 33 | 75 | 18 | 290 | 218 | 75 | 100 |
| Mid Neutralizer | 36 | T | 369 | 30 | 464 | >500 | 282 | 117 | 240 | 16 | 128 | >500 | 77 | >500 | >500 | 119 | 10 | 294 | 121 | 144 | 209 | 194 | 119 | 80 |
| Mid Neutralizer | 37 | C | 144 | >300 | >300 | 195 | >300 | 282 | >300 | 178 | 115 | 216 | 67 | 54 | 26 | 249 | 129 | 196 | >300 | 214 | >300 | >300 | 134 | 65 |
| Mid Neutralizer | 38 | G | 59 | 126 | >500 | 292 | 100 | 209 | 94 | 83 | 162 | 443 | 86 | >500 | >500 | 54 | 21 | 78 | >500 | 255 | 386 | 138 | 123 | 80 |
| Mid Neutralizer | 39 | T | >500 | 96 | 40 | >500 | >500 | >500 | 68 | >500 | 120 | 83 | 105 | 13 | 23 | 86 | 59 | 26 | 438 | 149 | >500 | >500 | 68 | 65 |
| Mid Neutralizer | 40 | N | >500 | 106 | >500 | >500 | 342 | 124 | >500 | 363 | >500 | >500 | 111 | >500 | >500 | 295 | 44 | 268 | 174 | 323 | >500 | 89 | 169 | 55 |
| Mid Neutralizer | 42 | T | >500 | 186 | 45 | 215 | 156 | 345 | 493 | 12 | >500 | >500 | 12 | 310 | >500 | 11 | 5 | 14 | >500 | >500 | 69 | >500 | 60 | 65 |
| Mid Neutralizer | 45 | T | 69 | 41 | 238 | 165 | 252 | 155 | 25 | 112 | 299 | 374 | 274 | >500 | 311 | 30 | 44 | 91 | 36 | 87 | >500 | 170 | 112 | 90 |
| Mid Neutralizer | 46 | T | >500 | >500 | >500 | >500 | 347 | 207 | 148 | >500 | 289 | >500 | 215 | 51 | 209 | 129 | 207 | >500 | >500 | 359 | >500 | >500 | 192 | 50 |
| Low Neutralizer | 47 | T | >500 | >500 | >500 | >500 | >500 | >500 | 188 | >500 | 168 | >500 | >500 | >500 | 207 | 53 | >500 | >500 | 397 | 153 | >500 | >500 | 166 | 30 |
| Low Neutralizer | 53 | T | 12 | 116 | >500 | 46 | 14 | 34 | 230 | >500 | 75 | 98 | >500 | 75 | 46 | >500 | 19 | 33 | 24 | >500 | 153 | >500 | 49 | 70 |
| Low Neutralizer | 58 | G | 152 | 24 | >500 | >500 | 75 | 163 | 102 | 334 | 62 | 338 | 103 | >500 | >500 | 90 | 12 | 429 | >500 | >500 | 71 | 324 | 109 | 70 |
| Low Neutralizer | 71 | G | 139 | 187 | >500 | >500 | 403 | >500 | 121 | 116 | 54 | >500 | 359 | >500 | >500 | 81 | 218 | 128 | >500 | >500 | >500 | >500 | 152 | 50 |
| Low Neutralizer | 76 | G | 21 | 34 | 340 | 111 | 20 | 9 | 80 | 30 | 17 | >500 | >500 | 45 | 7 | 50 | 19 | >500 | >500 | 349 | >500 | 27 | 38 | 75 |
| Low Neutralizer | 77 | G | 82 | 157 | >500 | 61 | 126 | 8 | 72 | 171 | >500 | 280 | >500 | 456 | >500 | >500 | 90 | 485 | 30 | >500 | >500 | 62 | 100 | 65 |
| Low Neutralizer | 82 | G | >500 | 202 | >500 | >500 | >500 | >500 | 172 | >500 | 314 | >500 | 88 | 101 | 120 | 320 | 175 | >500 | >500 | >500 | >500 | >500 | 168 | 40 |
| Low Neutralizer | 84 | G | 421 | 337 | >500 | 318 | 247 | 51 | 268 | 248 | 55 | 478 | 84 | 237 | 44 | 227 | 35 | 224 | 250 | 42 | 153 | 62 | 147 | 95 |
| Low Neutralizer | 87 | G | 123 | 35 | >500 | >500 | >500 | 37 | 30 | 159 | 343 | 56 | 117 | 270 | 116 | 245 | 41 | >500 | >500 | 185 | 239 | 27 | 97 | 75 |
| Low Neutralizer | 90 | G | 395 | 469 | 442 | >500 | 255 | 133 | 95 | 141 | 412 | 334 | 124 | >500 | >500 | 77 | 84 | >500 | 141 | >500 | >500 | 183 | 194 | 70 |
| Low Neutralizer | 101 | G | 109 | 273 | >500 | >500 | 461 | 383 | 87 | 336 | 90 | >500 | >500 | 88 | >500 | 379 | 179 | 437 | >500 | 449 | 254 | >500 | 226 | 65 |
| Low Neutralizer | 104 | G | >500 | 287 | >500 | >500 | >500 | >500 | 274 | >500 | 414 | >500 | 222 | 84 | 420 | 342 | >500 | >500 | >500 | 224 | >500 | >500 | 258 | 40 |
| Low Neutralizer | 111 | G | >500 | 335 | >500 | >500 | >500 | 133 | 48 | 15 | 197 | >500 | 206 | >500 | 90 | 62 | 111 | 97 | 436 | 69 | >500 | >500 | 107 | 60 |
| Low Neutralizer | 139 | G | >500 | 99 | >500 | >500 | >500 | >500 | >500 | 108 | 172 | >500 | 335 | 62 | 290 | >500 | 393 | >500 | >500 | >500 | >500 | >500 | 171 | 35 |
| Low Neutralizer | 173 | G | >500 | 310 | >500 | >500 | >500 | >500 | >500 | >500 | >500 | 241 | 255 | 89 | 371 | >500 | 87 | >500 | 382 | 300 | >500 | 498 | 245 | 45 |
| Low Neutralizer | 189 | G | 290 | >500 | >500 | 63 | 49 | 64 | 64 | 60 | 9 | >500 | >500 | 448 | 16 | 265 | 166 | 37 | >500 | >500 | >500 | >500 | 73 | 60 |
| Low Neutralizer | 294 | G | >500 | >500 | >500 | 43 | 103 | 13 | 58 | 30 | >500 | >500 | >500 | >500 | >500 | 156 | >500 | >500 | >500 | >500 | >500 | 480 | 69 | 35 |

IC50 (µg/ml): < 25 | 25-100 | 100-300 | 300-500 | > 500

## Epitope Mapping

| | Rank of patient | Cohort | VRC01-like | b12-like | HJ16-like | 8ANC195-like | PG9-like | PGT128-like | 2F5-like | 10E8-like | 35O22-like | PGT151-like |
|---|---|---|---|---|---|---|---|---|---|---|---|---|
| Mid Neutralizer | 26 | G | 43 | | 3 | | 40 | | 12 | 1 | | |
| Mid Neutralizer | 27 | N | 60 | | 8 | | 8 | | 7 | 13 | | 6 |
| Mid Neutralizer | 28 | T | 3 | 14 | | | 13 | 23 | | 37 | | 11 |
| Mid Neutralizer | 29 | T | 7 | 8 | | | 59 | | | 25 | | |
| Mid Neutralizer | 30 | N | 29 | | | | 36 | 20 | | 15 | | |
| Mid Neutralizer | 31 | G | 36 | | 25 | | | | | 32 | | 7 |
| Mid Neutralizer | 33 | T | 5 | | 10 | | | | 36 | 18 | | 31 |
| Mid Neutralizer | 34 | T | 11 | | | 8 | 81 | | | | | |
| Mid Neutralizer | 35 | T | 20 | 1 | | | 20 | 7 | | 37 | | 15 |
| Mid Neutralizer | 36 | T | 80 | | | | | | 3 | 10 | | 6 |
| Mid Neutralizer | 37 | C | | | | | | 68 | 1 | 7 | | 24 |
| Mid Neutralizer | 38 | G | 50 | | | | 26 | | | 20 | | 4 |
| Mid Neutralizer | 39 | T | | | 14 | | | 72 | | 9 | | 5 |
| Mid Neutralizer | 40 | N | 66 | | | | 19 | 1 | | 14 | 1 | |
| Mid Neutralizer | 42 | T | 55 | 17 | 22 | 6 | | | | | | |
| Mid Neutralizer | 45 | T | 43 | | | | 17 | | | 38 | 2 | |
| Mid Neutralizer | 46 | T | 7 | 15 | | 6 | 24 | 18 | | 31 | | |
| Low Neutralizer | 47 | T | | | | | 27 | 7 | | 47 | | 19 |
| Low Neutralizer | 53 | T | 1 | 13 | | | 57 | 18 | 12 | | | |
| Low Neutralizer | 58 | G | 30 | 1 | | 7 | 22 | | 10 | | | 30 |
| Low Neutralizer | 71 | G | 29 | | 11 | 1 | 20 | | 13 | 12 | | 15 |
| Low Neutralizer | 76 | G | 5 | | | | 51 | | 28 | 4 | | 12 |
| Low Neutralizer | 77 | G | 18 | | | | 19 | | 41 | 12 | 10 | |
| Low Neutralizer | 82 | G | 18 | 15 | | 2 | 10 | 43 | 5 | 2 | | 5 |
| Low Neutralizer | 84 | G | 11 | 24 | | | 22 | 16 | | 16 | | 11 |
| Low Neutralizer | 87 | G | 25 | | | | 3 | 27 | 34 | 12 | | |
| Low Neutralizer | 90 | G | 40 | | | 13 | 23 | | | 24 | | |
| Low Neutralizer | 101 | G | 14 | 13 | | | | | 1 | 19 | | 52 |
| Low Neutralizer | 104 | G | | 34 | | | | 27 | | 34 | | 5 |
| Low Neutralizer | 111 | G | 45 | | | | 8 | 4 | | 44 | | |
| Low Neutralizer | 139 | G | 21 | 12 | | | | 39 | 18 | | | 9 |
| Low Neutralizer | 173 | G | 8 | | | | | 81 | | 11 | | |
| Low Neutralizer | 189 | G | | 2 | 25 | | 44 | | 22 | 6 | | |
| Low Neutralizer | 294 | G | 15 | 2 | | | 4 | | 36 | 44 | | |

**Supplementary Table 2**. IgG neutralizing activity of selected participants against the global panel during cohort scre

Baseline visit

| ID | % Neutralization 398F1 | 246F3 | CNE55 | CNE8 | X2278 | Tro11 | BJOX2000 | CH119 | CE1176 | CE0217 | 25710 | X1632 | Mean % Neutr. | Coverage (%) |
|---|---|---|---|---|---|---|---|---|---|---|---|---|---|---|
| IDC#0014 | 66 | 79 | 79 | 87 | 94 | 87 | 47 | 49 | 57 | 79 | 84 | 53 | 72 | 83 |
| IDC#0016 | 66 | 24 | 48 | 41 | 81 | 50 | 42 | 68 | 47 | 16 | 74 | 46 | 50 | 33 |
| IDC#0035 | 81 | 66 | 36 | 24 | 54 | 26 | 43 | 51 | 71 | 32 | 59 | 59 | 50 | 58 |
| IDC#0038 | 91 | 65 | 43 | 68 | 57 | 30 | 44 | 53 | 18 | 72 | 86 | 40 | 56 | 58 |
| IDC#0042 | 92 | 77 | 72 | 78 | 90 | 59 | 54 | 32 | 26 | 41 | 68 | 53 | 62 | 75 |
| IDC#0067 | 53 | 45 | 34 | 65 | 23 | 38 | 36 | 48 | 46 | 42 | 68 | 8 | 42 | 25 |
| IDC#0094 | 57 | 42 | 9 | 26 | 64 | 61 | 59 | 68 | 67 | 10 | 65 | 13 | 45 | 58 |
| IDC#0124 | 73 | 50 | 14 | 21 | 39 | 14 | 53 | 69 | 48 | 30 | 80 | 41 | 44 | 42 |
| IDC#0134 | 96 | 67 | 51 | 58 | 46 | 47 | 36 | 59 | 45 | 57 | 88 | 69 | 60 | 67 |
| IDC#0136 | 97 | 98 | 46 | 44 | 100 | 86 | 99 | 94 | 100 | 100 | 91 | 97 | 88 | 83 |
| IDC#0144 | 62 | 86 | 12 | 15 | 76 | 59 | 34 | 32 | 26 | 23 | 20 | 45 | 41 | 33 |
| IDC#0151 | 89 | 60 | 58 | 28 | 26 | 47 | 38 | 56 | 56 | 48 | 79 | 26 | 51 | 50 |
| IDC#0193 | 74 | 32 | 16 | 25 | 32 | 83 | 49 | 28 | 86 | 26 | 70 | 17 | 45 | 33 |
| IDC#0199 | 88 | 66 | 61 | 54 | 90 | 85 | 43 | 72 | 95 | 18 | 84 | 39 | 66 | 75 |
| IDC#0215 | 63 | 45 | 43 | 42 | 70 | 65 | 45 | 70 | 35 | 22 | 53 | 23 | 48 | 42 |
| IDC#0237 | 72 | 7 | 1 | 0 | 10 | 70 | 76 | 83 | 100 | 72 | 100 | 0 | 49 | 58 |
| IDC#0277 | 78 | 44 | 17 | 24 | 80 | 54 | 61 | 42 | 22 | 14 | 42 | 44 | 44 | 33 |
| IDC#0336 | 90 | 76 | 21 | 43 | 38 | 52 | 66 | 73 | 79 | 23 | 79 | 36 | 56 | 58 |
| IDC#0337 | 95 | 94 | 9 | 48 | 48 | 87 | 71 | 28 | 96 | 67 | 81 | 43 | 64 | 58 |
| IDC#0345 | 54 | 54 | 43 | 81 | 37 | 57 | 31 | 36 | 18 | 19 | 72 | 23 | 44 | 42 |
| IDC#0358 | 86 | 33 | 27 | 29 | 47 | 47 | 53 | 46 | 21 | 35 | 24 | 46 | 41 | 17 |
| IDC#0359 | 94 | 50 | 40 | 56 | 58 | 81 | 46 | 67 | 59 | 60 | 87 | 38 | 61 | 75 |
| IDC#0388 | 78 | 72 | 10 | 59 | 68 | 42 | 53 | 59 | 51 | 7 | 70 | 28 | 50 | 67 |
| IDC#0397 | 58 | 34 | 69 | 67 | 48 | 60 | 30 | 94 | 16 | 38 | 69 | 49 | 53 | 50 |
| IDC#0398 | 82 | 62 | 28 | 35 | 53 | 46 | 58 | 50 | 26 | 28 | 50 | 37 | 46 | 42 |
| IDC#0423 | 76 | 83 | 20 | 34 | 59 | 65 | 85 | 95 | 24 | 18 | 61 | 28 | 54 | 58 |
| IDC#0434 | 90 | 59 | 21 | 40 | 47 | 64 | 78 | 80 | 29 | 24 | 77 | 54 | 55 | 58 |
| IDC#0441 | 59 | 51 | 39 | 68 | 59 | 60 | 33 | 41 | 39 | 20 | 63 | 30 | 47 | 50 |
| IDC#0444 | 93 | 93 | 84 | 73 | 98 | 98 | 86 | 99 | 60 | 40 | 83 | 78 | 82 | 92 |
| IDC#0465 | 56 | 49 | 33 | 61 | 65 | 69 | 37 | 37 | 27 | 63 | 57 | 24 | 48 | 50 |
| IDC#0468 | 98 | 33 | 17 | 66 | 47 | 50 | 67 | 75 | 33 | 12 | 75 | 18 | 49 | 50 |
| IDC#0476 | 63 | 54 | 60 | 53 | 96 | 65 | 65 | 36 | 58 | 60 | 60 | 66 | 61 | 92 |
| IDC#0478 | 96 | 42 | 20 | 21 | 51 | 68 | 59 | 44 | 84 | 58 | 92 | 5 | 53 | 58 |
| IDC#0481 | 55 | 42 | 49 | 36 | 94 | 60 | 25 | 49 | 72 | 82 | 79 | 36 | 57 | 50 |
| IDC#0490 | 77 | 62 | 59 | 66 | 67 | 68 | 54 | 71 | 56 | 61 | 92 | 60 | 66 | 100 |
| IDC#0508 | 94 | 98 | 96 | 95 | 92 | 90 | 59 | 89 | 69 | 79 | 87 | 83 | 86 | 100 |
| IDC#0513 | 68 | 60 | 73 | 79 | 93 | 88 | 62 | 72 | 59 | 32 | 83 | 71 | 70 | 92 |
| IDC#0518 | 83 | 83 | 72 | 74 | 70 | 76 | 49 | 55 | 71 | 27 | 90 | 58 | 68 | 83 |
| IDC#0519 | 67 | 71 | 13 | 16 | 87 | 55 | 43 | 60 | 45 | 41 | 78 | 30 | 50 | 50 |
| IDC#0526 | 99 | 36 | 0 | 0 | 77 | 91 | 98 | 91 | 88 | 57 | 75 | 14 | 61 | 67 |
| IDC#0527 | 81 | 91 | 70 | 69 | 96 | 69 | 83 | 96 | 75 | 48 | 84 | 88 | 79 | 92 |
| IDC#0529 | 100 | 42 | 30 | 52 | 50 | 69 | 44 | 45 | 34 | 29 | 53 | 42 | 49 | 33 |
| IDC#0539 | 59 | 57 | 9 | 16 | 83 | 26 | 72 | 52 | 50 | 37 | 28 | 3 | 41 | 42 |
| IDC#0543 | 100 | 92 | 4 | 17 | 23 | 75 | 93 | 62 | 61 | 4 | 43 | 63 | 53 | 58 |
| IDC#0546 | 97 | 90 | 50 | 52 | 65 | 69 | 57 | 64 | 78 | 49 | 89 | 62 | 69 | 83 |
| IDC#0549 | 62 | 56 | 55 | 55 | 57 | 61 | 48 | 53 | 50 | 59 | 84 | 36 | 56 | 75 |
| IDC#0561 | 71 | 89 | 92 | 85 | 99 | 97 | 93 | 82 | 51 | 83 | 87 | 85 | 84 | 100 |
| IDC#0562 | 74 | 62 | 54 | 54 | 39 | 36 | 52 | 52 | 27 | 17 | 90 | 61 | 52 | 67 |
| IDC#0577 | 59 | 32 | 65 | 48 | 55 | 41 | 51 | 47 | 14 | 0 | 74 | 39 | 44 | 42 |
| IDC#0586 | 90 | 66 | 22 | 40 | 37 | 27 | 44 | 35 | 20 | 10 | 67 | 40 | 42 | 25 |
| IDC#0591 | 100 | 96 | 41 | 50 | 47 | 58 | 82 | 51 | 31 | 52 | 68 | 64 | 62 | 67 |
| IDC#0609 | 97 | 57 | 32 | 34 | 62 | 81 | 64 | 69 | 83 | 71 | 86 | 73 | 67 | 83 |
| IDC#0629 | 95 | 70 | 36 | 44 | 48 | 28 | 38 | 37 | 25 | 44 | 66 | 46 | 48 | 25 |
| IDC#0638 | 59 | 47 | 21 | 47 | 34 | 56 | 39 | 33 | 18 | 34 | 43 | 62 | 41 | 25 |
| IDC#0639 | 68 | 37 | 20 | 17 | 26 | 20 | 56 | 61 | 49 | 23 | 72 | 45 | 41 | 33 |
| IDC#0654 | 92 | 67 | 53 | 27 | 44 | 60 | 41 | 30 | 20 | 21 | 74 | 26 | 46 | 42 |
| IDC#0659 | 52 | 43 | 29 | 51 | 79 | 48 | 46 | 42 | 35 | 59 | 79 | 30 | 49 | 42 |
| IDC#0666 | 96 | 51 | 4 | 9 | 94 | 83 | 95 | 90 | 91 | 27 | 38 | 29 | 59 | 58 |
| IDC#0669 | 98 | 99 | 6 | 35 | 33 | 56 | 90 | 91 | 75 | 76 | 31 | 96 | 66 | 67 |
| IDC#0686 | 60 | 57 | 34 | 45 | 53 | 83 | 42 | 37 | 26 | 46 | 90 | 40 | 51 | 42 |
| IDC#0703 | 72 | 43 | 45 | 54 | 54 | 46 | 43 | 42 | 38 | 47 | 76 | 37 | 50 | 33 |
| IDC#0706 | 49 | 40 | 38 | 54 | 66 | 39 | 27 | 40 | 37 | 72 | 86 | 33 | 48 | 33 |
| IDC#0707 | 99 | 77 | 29 | 50 | 84 | 95 | 96 | 66 | 28 | 26 | 77 | 20 | 62 | 58 |
| IDC#0768 | 99 | 12 | 54 | 0 | 94 | 88 | 97 | 87 | 97 | 54 | 97 | 27 | 67 | 75 |
| IDC#0782 | 89 | 74 | 27 | 33 | 37 | 61 | 84 | 89 | 77 | 23 | 89 | 51 | 61 | 67 |
| IDC#0786 | 61 | 97 | 93 | 83 | 40 | 21 | 98 | 66 | 0 | 25 | 90 | 81 | 63 | 67 |
| IDC#0792 | 66 | 18 | 55 | 24 | 49 | 45 | 48 | 31 | 18 | 41 | 71 | 31 | 42 | 25 |
| IDC#0796 | 76 | 54 | 58 | 29 | 74 | 68 | 36 | 76 | 46 | 24 | 79 | 43 | 55 | 58 |
| IDE#0057 | 72 | 89 | 78 | 94 | 89 | 82 | 83 | 68 | 79 | 74 | 95 | 64 | 81 | 100 |
| IDE#0294 | 99 | 89 | 75 | 90 | 98 | 96 | 92 | 95 | 87 | 79 | 99 | 93 | 91 | 100 |
| IDF#0033 | 82 | 97 | 73 | 96 | 95 | 99 | 85 | 57 | 94 | 89 | 93 | 68 | 86 | 100 |

| % Neutr. | Breadth (%) |
|---|---|
| >90 | >90 |
| 75-90 | 80-90 |
| 50-74 | 50-80 |
| 25-49 | 25-49 |
| <25 | <50 |

**Supplementary Table 3**. IgG neutralizing activity of selected participants against the global panel.

**A)** Baseline visit

| ID | Δ to 1st timepoint (years) | IC$_{50}$(µg/ml) 398F1 | 246F3 | CNE55 | CNE8 | X2278 | Tro11 | BJOX2000 | CH119 | CE1176 | CE0217 | 25710 | X1632 | Geo. Mean of detected | Coverage (%) |
|---|---|---|---|---|---|---|---|---|---|---|---|---|---|---|---|
| IDC#0014 | 0 | 145 | 88 | 137 | 50 | 45 | 53 | 420 | 381 | 275 | 114 | 75 | 279 | 129 | 100 |
| IDC#0016 | 0 | 380 | >500 | 445 | 409 | 98 | 349 | 474 | 139 | 337 | >500 | 132 | >500 | 267 | 75 |
| IDC#0035 | 0 | 141 | 188 | >500 | >500 | >500 | >500 | >500 | 383 | 186 | >500 | 186 | 443 | 232 | 50 |
| IDC#0038 | 0 | 49 | 106 | 472 | 44 | 190 | 466 | 461 | 312 | >500 | 115 | 45 | 428 | 168 | 92 |
| IDC#0042 | 0 | 50 | 59 | 83 | 48 | 43 | 160 | 413 | >500 | >500 | 442 | 89 | 241 | 112 | 83 |
| IDC#0067 | 0 | 252 | 260 | 427 | 70 | >500 | 324 | 439 | 447 | 338 | 483 | 80 | >500 | 264 | 83 |
| IDC#0094 | 0 | 154 | 286 | >500 | >500 | 141 | 247 | 190 | 91 | 158 | >500 | 171 | >500 | 170 | 67 |
| IDC#0124 | 0 | 4 | 458 | >500 | >500 | >500 | >500 | 483 | 187 | 423 | >500 | 71 | 488 | 160 | 58 |
| IDC#0134 | 0 | 30 | 264 | 317 | 327 | >500 | 340 | >500 | 307 | 435 | 359 | 57 | 263 | 214 | 83 |
| IDC#0136 | 0 | 12 | 18 | 469 | >500 | 10 | 106 | 14 | 86 | 5 | 19 | 56 | 37 | 31 | 92 |
| IDC#0144 | 0 | 188 | 154 | >500 | >500 | 240 | 271 | >500 | >500 | >500 | >500 | >500 | >500 | 208 | 33 |
| IDC#0151 | 0 | 85 | 276 | 286 | 458 | >500 | 325 | 277 | 326 | 320 | 435 | 95 | >500 | 256 | 83 |
| IDC#0193 | 0 | 156 | 345 | >500 | 385 | >500 | 50 | 261 | 383 | 90 | 490 | 103 | >500 | 198 | 75 |
| IDC#0199 | 0 | 103 | 212 | 196 | 128 | 80 | 52 | 497 | 149 | 52 | >500 | 62 | >500 | 118 | 83 |
| IDC#0215 | 0 | 321 | 410 | 434 | 346 | 179 | 151 | >500 | 122 | 440 | 484 | 230 | >500 | 282 | 83 |
| IDC#0237 | 0 | 155 | >500 | >500 | >500 | >500 | 108 | 119 | 74 | 13 | 133 | 8 | >500 | 56 | 58 |
| IDC#0277 | 0 | 68 | 326 | >500 | >500 | 69 | 154 | 182 | 435 | >500 | >500 | 285 | 357 | 193 | 67 |
| IDC#0336 | 0 | 30 | 117 | >500 | 359 | >500 | 358 | 108 | 99 | 89 | >500 | 70 | 490 | 134 | 75 |
| IDC#0337 | 0 | 85 | 160 | >500 | >500 | >500 | 224 | 368 | >500 | 195 | 457 | 209 | >500 | 214 | 58 |
| IDC#0345 | 0 | 262 | 423 | >500 | 197 | >500 | 363 | >500 | >500 | >500 | >500 | 126 | >500 | 251 | 42 |
| IDC#0358 | 0 | 75 | >500 | >500 | 371 | 429 | 294 | 247 | 411 | >500 | 400 | 377 | 376 | 301 | 75 |
| IDC#0359 | 0 | 20 | 234 | 433 | 182 | 91 | 81 | 239 | 128 | 176 | 280 | 33 | >500 | 126 | 92 |
| IDC#0388 | 0 | 142 | 192 | >500 | 206 | 260 | 318 | 290 | 182 | 331 | >500 | 73 | >500 | 203 | 75 |
| IDC#0397 | 0 | 285 | >500 | 278 | 240 | 441 | 209 | >500 | 50 | >500 | 467 | 99 | 396 | 227 | 75 |
| IDC#0398 | 0 | 125 | 274 | >500 | 383 | >500 | 350 | 413 | 348 | >500 | >500 | 195 | >500 | 278 | 58 |
| IDC#0423 | 0 | 104 | 86 | >500 | 446 | 273 | 88 | 77 | 51 | 457 | 368 | 145 | >500 | 157 | 83 |
| IDC#0434 | 0 | 29 | 242 | >500 | 249 | >500 | 229 | 136 | 113 | >500 | >500 | 118 | 364 | 150 | 67 |
| IDC#0441 | 0 | >500 | >500 | >500 | 89 | >500 | 312 | >500 | >500 | >500 | >500 | 410 | >500 | 225 | 25 |
| IDC#0444 | 0 | 29 | 65 | 192 | 242 | 56 | 25 | 147 | 54 | 325 | >500 | 110 | 151 | 94 | 92 |
| IDC#0465 | 0 | 63 | 378 | >500 | 118 | 106 | 256 | >500 | >500 | >500 | 240 | 321 | >500 | 179 | 58 |
| IDC#0468 | 0 | 12 | 388 | >500 | 62 | 450 | 224 | 153 | 82 | 457 | >500 | 65 | >500 | 131 | 75 |
| IDC#0476 | 0 | 228 | 344 | 411 | 497 | 57 | 260 | 346 | >500 | 333 | 382 | 279 | 267 | 278 | 92 |
| IDC#0478 | 0 | 71 | >500 | >500 | >500 | 425 | 214 | 344 | >500 | 129 | 329 | 74 | >500 | 183 | 58 |
| IDC#0481 | 0 | 249 | >500 | >500 | 366 | 66 | 315 | >500 | >500 | 200 | 135 | 88 | >500 | 172 | 58 |
| IDC#0490 | 0 | 96 | 183 | 113 | 28 | 143 | 58 | 182 | 54 | 120 | 166 | 12 | 118 | 84 | 100 |
| IDC#0508 | 0 | 56 | 59 | 36 | 29 | 114 | 74 | 399 | 85 | 249 | 139 | 93 | 139 | 93 | 100 |
| IDC#0513 | 0 | 160 | 261 | 102 | 57 | 73 | 53 | 254 | 145 | 241 | >500 | 81 | 258 | 130 | 92 |
| IDC#0518 | 0 | 33 | 39 | 97 | 32 | 140 | 80 | 275 | 181 | 113 | >500 | 26 | 268 | 85 | 92 |
| IDC#0519 | 0 | 92 | 142 | >500 | >500 | 77 | 165 | 447 | 202 | 457 | 321 | 72 | >500 | 175 | 75 |
| IDC#0526 | 0 | 57 | 499 | >500 | >500 | 216 | 100 | 86 | 135 | 206 | >500 | 222 | >500 | 155 | 67 |
| IDC#0527 | 0 | 128 | 70 | 203 | 274 | 78 | 162 | 109 | 39 | 226 | >500 | 157 | 106 | 123 | 92 |
| IDC#0529 | 0 | 7 | 496 | >500 | 394 | 414 | 40 | 463 | 303 | >500 | >500 | 246 | >500 | 170 | 67 |
| IDC#0539 | 0 | 150 | 250 | >500 | >500 | 143 | >500 | 194 | 340 | 439 | >500 | >500 | >500 | 232 | 50 |
| IDC#0543 | 0 | <3,9 | 37 | >500 | >500 | >500 | 103 | 42 | 229 | 300 | >500 | 378 | 184 | 86 | 67 |
| IDC#0546 | 0 | 23 | 101 | >500 | 399 | 359 | 271 | 291 | 284 | 204 | 434 | 85 | 334 | 198 | 92 |
| IDC#0549 | 0 | 9 | 422 | 291 | 383 | >500 | 299 | 423 | 355 | 387 | 292 | 103 | 467 | 230 | 92 |
| IDC#0561 | 0 | 106 | 122 | 89 | 117 | 44 | 50 | 81 | 146 | 489 | 155 | 79 | 122 | 108 | 100 |
| IDC#0562 | 0 | 39 | 99 | 179 | 56 | >500 | 263 | 168 | 250 | 486 | >500 | 23 | 236 | 127 | 83 |
| IDC#0577 | 0 | 145 | 448 | 395 | 228 | 355 | 319 | 398 | 442 | >500 | >500 | 82 | 472 | 292 | 83 |
| IDC#0586 | 0 | 78 | 320 | >500 | 387 | >500 | >500 | >500 | >500 | >500 | >500 | 227 | >500 | 216 | 33 |
| IDC#0591 | 0 | 25 | 60 | 470 | 216 | >500 | 266 | 170 | 429 | 475 | 482 | 213 | 347 | 217 | 92 |
| IDC#0609 | 0 | 14 | 243 | >500 | 300 | 469 | 88 | 247 | 225 | 222 | 228 | 46 | 220 | 154 | 92 |
| IDC#0629 | 0 | 17 | 213 | 422 | 278 | >500 | 429 | 363 | 380 | 352 | 316 | 163 | 382 | 242 | 92 |
| IDC#0638 | 0 | 303 | 443 | >500 | 242 | >500 | 266 | >500 | >500 | >500 | >500 | 381 | 111 | 267 | 50 |
| IDC#0639 | 0 | 133 | >500 | >500 | >500 | >500 | >500 | 299 | 305 | 319 | >500 | 148 | 416 | 249 | 50 |
| IDC#0654 | 0 | 44 | 220 | 437 | 406 | >500 | 265 | >500 | >500 | >500 | >500 | 151 | >500 | 202 | 50 |
| IDC#0659 | 0 | 70 | 191 | 457 | 82 | 54 | 108 | 237 | 403 | 380 | 219 | 40 | 443 | 165 | 100 |
| IDC#0666 | 0 | 6 | 367 | >500 | >500 | 6 | <3,9 | 7 | 56 | 59 | >500 | 357 | >500 | 28 | 67 |
| IDC#0669 | 0 | 32 | 14 | >500 | 399 | >500 | 191 | 75 | 92 | 114 | 127 | >500 | 26 | 76 | 75 |
| IDC#0686 | 0 | 166 | 262 | >500 | 342 | 387 | 67 | >500 | >500 | >500 | 294 | 82 | 449 | 212 | 67 |
| IDC#0703 | 0 | >500 | 369 | >500 | 99 | 266 | 490 | >500 | 476 | >500 | 179 | 107 | 422 | 256 | 67 |
| IDC#0706 | 0 | 179 | 368 | >500 | 276 | 383 | 454 | >500 | >500 | 419 | 201 | 75 | 357 | 268 | 75 |
| IDC#0707 | 0 | <3,9 | 68 | >500 | 181 | 96 | 37 | 41 | 234 | 490 | >500 | 133 | >500 | 78 | 75 |
| IDC#0768 | 0 | 17 | >500 | 352 | >500 | 59 | 73 | 61 | 99 | 44 | 375 | 28 | >500 | 74 | 75 |
| IDC#0782 | 0 | 23 | 68 | >500 | 158 | 500 | 234 | 31 | 22 | 54 | 353 | 24 | 222 | 87 | 92 |
| IDC#0786 | 0 | 309 | <3,9 | 4 | 59 | >500 | >500 | 5 | 130 | >500 | >500 | 12 | 26 | 22 | 67 |
| IDC#0792 | 0 | 189 | 421 | 270 | >500 | >500 | 422 | 410 | >500 | >500 | 283 | 93 | >500 | 267 | 58 |
| IDC#0796 | 0 | 23 | 161 | 74 | 282 | 43 | 184 | 460 | 50 | 416 | >500 | 16 | 378 | 112 | 92 |
| IDE#0057 | 0 | 70 | 79 | 164 | 36 | 61 | 141 | 103 | 203 | 142 | 232 | 27 | 253 | 102 | 100 |
| IDE#0294 | 0 | <3,9 | 58 | 188 | 31 | 20 | 39 | 76 | 56 | 91 | 204 | 9 | 29 | 41 | 100 |
| IDF#0033 | 0 | 35 | 41 | 227 | 56 | 51 | 32 | 186 | 384 | 89 | 57 | 83 | 94 | 81 | 100 |

| **IC$_{50}$** (µg/ml) | **Breadth** |
|---|---|
| <25 | >95 |
| 25-100 | 80-95 |
| 100-300 | 65-79 |
| 300-500 | 50-64 |
| >500 | <50 |

**Supplementary Table 3**. IgG neutralizing activity of selected participants against the global panel.

**B)** 2nd visit

| ID | Δ to 1st timepoint (years) | IC$_{50}$(µg/ml) 398F1 | 246F3 | CNE55 | CNE8 | X2278 | Tro11 | BJOX2000 | CH119 | CE1176 | CE0217 | 25710 | X1632 | Geo. Mean of detected | Coverage (%) |
|---|---|---|---|---|---|---|---|---|---|---|---|---|---|---|---|
| IDC#0014 | 1.5 | 186 | 93 | 132 | 56 | 46 | 64 | 419 | 373 | 304 | 133 | 100 | 239 | 140 | 100 |
| IDC#0016 | 1.5 | 435 | >500 | >500 | >500 | 172 | 498 | >500 | 315 | >500 | >500 | 217 | >500 | 303 | 42 |
| IDC#0035 | 1.5 | 187 | 201 | >500 | >500 | >500 | >500 | >500 | 479 | 204 | >500 | 172 | 422 | 254 | 50 |
| IDC#0038 | 1.6 | 46 | 114 | >500 | 57 | 438 | 313 | 458 | 275 | >500 | 106 | 56 | 406 | 162 | 83 |
| IDC#0042 | 1.5 | 58 | 179 | 417 | 278 | 134 | 457 | >500 | >500 | >500 | >500 | 248 | >500 | 210 | 58 |
| IDC#0067 | 2.6 | 316 | 458 | >500 | 287 | >500 | >500 | >500 | >500 | >500 | >500 | 442 | >500 | 368 | 33 |
| IDC#0094 | 3.4 | 228 | 364 | >500 | >500 | 171 | 380 | 215 | 136 | 225 | >500 | 158 | >500 | 220 | 67 |
| IDC#0124 | 1.5 | 14 | 447 | >500 | >500 | >500 | >500 | 458 | 170 | 423 | >500 | 68 | 480 | 183 | 58 |
| IDC#0134 | 1.4 | 37 | 316 | 465 | 370 | >500 | 477 | >500 | 269 | 475 | >500 | 84 | 443 | 255 | 75 |
| IDC#0136 | 0.9 | 20 | 58 | 446 | 483 | 17 | 109 | 32 | 102 | 18 | 36 | 91 | 65 | 65 | 100 |
| IDC#0144 | 1.5 | 240 | 97 | >500 | 476 | 177 | 198 | >500 | >500 | >500 | >500 | >500 | >500 | 208 | 42 |
| IDC#0151 | 1.5 | 97 | 274 | 371 | 419 | >500 | 315 | 335 | 365 | 327 | 482 | 102 | >500 | 276 | 83 |
| IDC#0193 | 1.3 | 261 | >500 | >500 | >500 | >500 | 95 | 417 | >500 | 123 | >500 | 145 | >500 | 179 | 42 |
| IDC#0199 | 2.1 | 151 | 451 | >500 | 276 | 118 | 103 | >500 | 170 | 57 | >500 | 163 | >500 | 156 | 67 |
| IDC#0215 | 1.4 | 347 | 441 | 358 | 438 | 286 | 182 | >500 | 133 | 461 | 473 | 192 | >500 | 305 | 83 |
| IDC#0237 | 1.5 | 127 | >500 | >500 | >500 | >500 | 95 | 117 | 108 | 13 | 154 | 9 | >500 | 59 | 58 |
| IDC#0277 | 1.9 | 63 | 405 | >500 | >500 | 135 | 274 | 265 | >500 | >500 | >500 | 428 | 418 | 239 | 58 |
| IDC#0336 | 2.9 | 40 | 223 | >500 | 495 | >500 | 387 | 177 | 127 | 139 | >500 | 85 | >500 | 161 | 67 |
| IDC#0337 | 1.4 | 66 | 129 | >500 | >500 | >500 | 146 | 411 | >500 | 137 | 467 | 190 | >500 | 180 | 58 |
| IDC#0345 | 3.2 | 420 | >500 | >500 | >500 | >500 | >500 | >500 | >500 | >500 | >500 | >500 | >500 | 420 | 8 |
| IDC#0358 | 1.4 | 105 | 451 | >500 | 483 | 371 | 320 | 238 | 399 | >500 | 387 | 343 | 398 | 326 | 83 |
| IDC#0359 | 2.9 | 12 | 119 | >500 | 146 | 46 | 50 | 406 | 79 | 228 | 363 | 39 | 180 | 98 | 92 |
| IDC#0388 | 1.5 | 165 | 246 | >500 | 271 | 334 | 416 | 362 | 312 | 446 | >500 | 105 | >500 | 272 | 75 |
| IDC#0397 | 1.2 | 349 | >500 | 344 | 376 | 484 | 259 | >500 | 57 | >500 | >500 | 141 | 434 | 258 | 67 |
| IDC#0398 | 1.5 | 208 | 346 | >500 | >500 | >500 | 465 | >500 | 496 | >500 | >500 | 342 | >500 | 355 | 42 |
| IDC#0423 | 1.9 | 122 | 280 | >500 | >500 | >500 | 166 | 170 | 133 | >500 | >500 | 227 | >500 | 175 | 50 |
| IDC#0434 | 2.8 | 38 | 330 | >500 | 376 | 471 | 408 | 248 | 242 | >500 | >500 | 146 | >500 | 230 | 67 |
| IDC#0441 | 1.5 | >500 | >500 | >500 | 75 | >500 | 467 | >500 | >500 | >500 | >500 | >500 | >500 | 187 | 17 |
| IDC#0444 | 1.6 | 83 | 82 | 178 | 173 | 61 | 39 | 160 | 61 | 370 | >500 | 110 | 175 | 112 | 92 |
| IDC#0465 | 2.1 | 135 | 469 | >500 | 287 | 266 | 408 | >500 | >500 | >500 | >500 | >500 | >500 | 287 | 42 |
| IDC#0468 | 1.1 | 26 | >500 | >500 | 177 | >500 | 464 | 372 | 263 | >500 | >500 | 202 | >500 | 187 | 50 |
| IDC#0476 | 1.6 | 245 | 340 | 223 | 409 | 64 | 257 | 324 | >500 | 399 | 363 | 145 | 266 | 249 | 92 |
| IDC#0478 | 1.5 | 64 | >500 | >500 | >500 | >500 | 200 | 366 | >500 | 122 | 392 | 85 | >500 | 163 | 50 |
| IDC#0481 | 1.1 | 339 | >500 | >500 | >500 | >500 | 466 | >500 | >500 | >500 | >500 | 221 | >500 | 327 | 25 |
| IDC#0490 | 1.2 | 177 | >500 | >500 | 412 | >500 | 181 | 481 | 164 | >500 | 472 | 55 | 436 | 242 | 67 |
| IDC#0508 | 1.2 | 26 | 49 | 22 | 56 | 84 | 36 | 357 | 105 | 191 | 146 | 81 | 138 | 78 | 100 |
| IDC#0513 | 0.9 | 150 | 452 | 275 | 309 | 115 | 120 | >500 | 347 | >500 | >500 | 152 | 493 | 233 | 75 |
| IDC#0518 | 2.2 | 113 | 158 | 386 | 153 | >500 | 408 | >500 | 473 | >500 | >500 | 318 | >500 | 252 | 58 |
| IDC#0519 | 1.2 | 92 | 208 | >500 | >500 | 147 | 262 | >500 | 238 | >500 | 427 | 97 | >500 | 184 | 58 |
| IDC#0526 | 1.4 | 22 | 431 | >500 | >500 | 184 | 63 | 56 | 111 | 104 | 402 | 151 | >500 | 118 | 75 |
| IDC#0527 | 1.0 | 94 | 104 | 244 | 184 | 60 | 126 | 115 | 49 | 186 | 147 | 103 | 107 | 115 | 100 |
| IDC#0529 | 1.5 | 6 | >500 | >500 | 421 | 450 | 48 | 480 | 326 | >500 | >500 | 253 | >500 | 155 | 58 |
| IDC#0539 | 1.4 | 147 | 308 | >500 | >500 | 173 | >500 | 217 | 373 | >500 | >500 | >500 | >500 | 229 | 42 |
| IDC#0543 | 1.0 | 6 | 42 | >500 | >500 | >500 | 240 | 81 | 311 | 444 | >500 | >500 | 249 | 107 | 58 |
| IDC#0546 | 1.0 | 24 | 92 | 422 | 387 | 340 | 165 | 293 | 223 | 203 | 391 | 69 | 232 | 185 | 100 |
| IDC#0549 | 1.7 | 11 | 307 | 335 | 396 | 293 | 295 | 455 | 414 | 363 | 307 | 85 | 444 | 237 | 100 |
| IDC#0561 | 1.1 | 167 | 143 | 112 | 150 | 54 | 60 | 113 | 238 | 469 | 224 | 125 | 161 | 143 | 100 |
| IDC#0562 | 1.9 | 232 | 349 | 303 | 172 | >500 | >500 | 368 | 399 | >500 | >500 | 77 | 344 | 252 | 67 |
| IDC#0577 | 2.0 | 141 | 456 | 452 | 328 | 467 | 408 | 477 | >500 | >500 | >500 | 157 | >500 | 328 | 67 |
| IDC#0586 | 1.2 | 81 | 406 | >500 | 424 | >500 | >500 | >500 | >500 | >500 | >500 | 256 | >500 | 244 | 33 |
| IDC#0591 | 0.8 | 14 | 51 | >500 | 344 | >500 | 321 | 157 | 466 | 498 | >500 | 198 | 330 | 179 | 75 |
| IDC#0609 | 0.8 | 6 | 196 | >500 | 298 | 351 | 36 | 212 | 121 | 155 | 190 | 28 | 224 | 107 | 92 |
| IDC#0629 | 1.0 | 24 | 165 | >500 | 271 | >500 | 300 | 420 | 355 | 441 | 356 | 153 | 303 | 226 | 83 |
| IDC#0638 | 1.0 | 159 | >500 | >500 | 367 | >500 | 374 | >500 | >500 | >500 | >500 | 454 | 187 | 284 | 42 |
| IDC#0639 | 1.0 | 284 | >500 | >500 | >500 | >500 | >500 | 310 | 299 | 304 | >500 | 137 | 420 | 278 | 50 |
| IDC#0654 | 0.9 | 36 | 348 | >500 | 451 | >500 | 258 | >500 | >500 | >500 | >500 | 187 | >500 | 194 | 42 |
| IDC#0659 | 2.8 | 82 | 299 | 439 | 92 | 92 | 284 | 330 | 420 | 402 | 227 | 39 | 471 | 206 | 100 |
| IDC#0666 | 0.8 | 4 | 319 | >500 | >500 | 7 | <3.91 | 4 | 37 | 57 | >500 | 363 | >500 | 25 | 67 |
| IDC#0669 | 0.9 | 25 | 23 | >500 | 429 | >500 | 221 | 91 | 97 | 174 | 205 | 451 | 37 | 111 | 83 |
| IDC#0686 | 1.0 | 150 | 286 | >500 | 321 | 441 | 86 | >500 | >500 | >500 | 363 | 76 | 413 | 222 | 67 |
| IDC#0703 | 0.9 | 207 | 471 | >500 | 361 | >500 | 468 | 497 | >500 | >500 | >500 | 244 | 437 | 365 | 58 |
| IDC#0706 | 0.7 | 234 | 302 | 471 | 230 | 335 | 357 | >500 | 367 | 397 | 169 | 66 | 342 | 268 | 92 |
| IDC#0707 | 2.7 | 15 | 109 | >500 | 321 | 290 | 105 | 162 | >500 | >500 | >500 | 221 | >500 | 128 | 58 |
| IDC#0768 | 0.8 | 13 | >500 | 375 | >500 | 66 | 54 | 40 | 76 | 34 | 281 | 25 | >500 | 61 | 75 |
| IDC#0782 | 1.6 | 21 | 94 | >500 | 296 | >500 | 453 | 57 | 51 | 95 | >500 | 49 | 181 | 95 | 75 |
| IDC#0786 | 0.5 | >500 | 5 | 8 | 164 | >500 | >500 | 17 | 321 | >500 | >500 | 21 | 58 | 33 | 58 |
| IDC#0792 | 0.6 | 245 | >500 | 441 | >500 | >500 | >500 | >500 | >500 | >500 | 498 | 164 | >500 | 306 | 33 |
| IDC#0796 | 1.3 | 54 | 431 | 193 | 334 | 261 | 377 | >500 | 150 | >500 | >500 | 158 | >500 | 208 | 67 |
| IDE#0057 | 4.0 | 138 | 485 | >500 | 405 | >500 | >500 | >500 | >500 | 478 | >500 | 313 | >500 | 332 | 42 |
| IDE#0294 | 3.0 | 8 | 91 | 396 | 63 | 50 | 72 | 114 | 160 | 236 | 367 | 22 | 66 | 86 | 100 |
| IDF#0033 | 2.8 | 25 | 36 | 69 | 72 | 52 | 28 | 179 | 207 | 62 | 63 | 66 | 188 | 69 | 100 |

| IC$_{50}$ (µg/ml) | Breadth |
|---|---|
| <25 | >95 |
| 25-100 | 80-95 |
| 100-300 | 65-79 |
| 300-500 | 50-64 |
| >500 | <50 |

**Supplementary Table 3**. IgG neutralizing activity of selected participants against the global panel.

**C)** 3rd visit

| ID | Δ to 1st timepoint (years) | IC50(µg/ml) 398F1 | 246F3 | CNE55 | CNE8 | X2278 | Tro11 | BJOX2000 | CH119 | CE1176 | CE0217 | 25710 | X1632 | Geo. Mean of detected | Coverage (%) |
|---|---|---|---|---|---|---|---|---|---|---|---|---|---|---|---|
| IDC#0014 | NA | NA | NA | NA | NA | NA | NA | NA | NA | NA | NA | NA | NA | NA | NA |
| IDC#0016 | 3.3 | 230 | >500 | >500 | 360 | 185 | >500 | 494 | 267 | >500 | >500 | 254 | >500 | 283 | 50 |
| IDC#0035 | 3.0 | 52 | 286 | >500 | 382 | >500 | >500 | >500 | >500 | 346 | >500 | 214 | >500 | 211 | 42 |
| IDC#0038 | 2.6 | 21 | 111 | >500 | 92 | 481 | 427 | 374 | >500 | >500 | >500 | 83 | >500 | 146 | 58 |
| IDC#0042 | 2.9 | 101 | 358 | >500 | 128 | 92 | 493 | >500 | >500 | >500 | >500 | 279 | >500 | 197 | 50 |
| IDC#0067 | 3.2 | 317 | 348 | >500 | 310 | >500 | >500 | >500 | 482 | >500 | >500 | 301 | >500 | 346 | 42 |
| IDC#0094 | NA | NA | NA | NA | NA | NA | NA | NA | NA | NA | NA | NA | NA | NA | NA |
| IDC#0124 | 2.9 | 121 | 246 | >500 | 357 | >500 | >500 | >500 | 174 | 427 | >500 | 79 | >500 | 199 | 50 |
| IDC#0134 | 2.7 | 54 | 337 | >500 | 277 | >500 | >500 | >500 | 353 | >500 | 482 | 80 | 463 | 227 | 58 |
| IDC#0136 | 1.4 | 13 | 64 | 352 | 76 | 37 | 257 | 52 | 176 | 14 | 72 | 113 | 92 | 72 | 100 |
| IDC#0144 | 2.5 | 93 | 88 | >500 | 450 | 80 | 174 | 421 | 452 | >500 | >500 | 268 | 437 | 219 | 75 |
| IDC#0151 | 2.5 | 21 | 411 | 431 | 425 | >500 | 320 | 249 | 251 | 356 | 451 | 286 | 497 | 276 | 92 |
| IDC#0193 | NA | NA | NA | NA | NA | NA | NA | NA | NA | NA | NA | NA | NA | NA | NA |
| IDC#0199 | NA | NA | NA | NA | NA | NA | NA | NA | NA | NA | NA | NA | NA | NA | NA |
| IDC#0215 | NA | NA | NA | NA | NA | NA | NA | NA | NA | NA | NA | NA | NA | NA | NA |
| IDC#0237 | 2.5 | 65 | >500 | >500 | >500 | >500 | 75 | 109 | 92 | 11 | 156 | 59 | >500 | 65 | 58 |
| IDC#0277 | 2.3 | 110 | 356 | >500 | >500 | 76 | 248 | 224 | >500 | >500 | >500 | 386 | 367 | 218 | 58 |
| IDC#0336 | NA | NA | NA | NA | NA | NA | NA | NA | NA | NA | NA | NA | NA | NA | NA |
| IDC#0337 | 2.4 | 33 | 87 | >500 | >500 | 337 | 162 | 338 | >500 | 103 | 410 | 240 | >500 | 165 | 67 |
| IDC#0345 | NA | NA | NA | NA | NA | NA | NA | NA | NA | NA | NA | NA | NA | NA | NA |
| IDC#0358 | NA | NA | NA | NA | NA | NA | NA | NA | NA | NA | NA | NA | NA | NA | NA |
| IDC#0359 | NA | NA | NA | NA | NA | NA | NA | NA | NA | NA | NA | NA | NA | NA | NA |
| IDC#0388 | 1.9 | 83 | 116 | >500 | 314 | 279 | 316 | 209 | 168 | 288 | >500 | 76 | >500 | 181 | 75 |
| IDC#0397 | 2.3 | 255 | 434 | 218 | 340 | 257 | 264 | 484 | 39 | >500 | >500 | 113 | 413 | 234 | 83 |
| IDC#0398 | 2.2 | 53 | 67 | 445 | 216 | 289 | 193 | 206 | 316 | 353 | 307 | 91 | 251 | 196 | 100 |
| IDC#0423 | 2.4 | 124 | 254 | >500 | 443 | 320 | 232 | 139 | 107 | >500 | >500 | 305 | >500 | 216 | 67 |
| IDC#0434 | NA | NA | NA | NA | NA | NA | NA | NA | NA | NA | NA | NA | NA | NA | NA |
| IDC#0441 | 2.2 | 290 | 250 | >500 | 62 | 439 | 400 | >500 | >500 | >500 | >500 | 365 | >500 | 257 | 50 |
| IDC#0444 | 2.2 | 7 | 101 | 286 | 198 | 29 | 45 | 125 | 57 | 400 | >500 | 169 | 224 | 95 | 92 |
| IDC#0465 | 3.0 | 85 | 468 | >500 | 193 | 354 | 490 | >500 | >500 | >500 | >500 | 445 | >500 | 290 | 50 |
| IDC#0468 | 2.3 | 267 | >500 | >500 | 250 | 255 | >500 | 478 | >500 | >500 | >500 | 417 | >500 | 321 | 42 |
| IDC#0476 | 2.8 | 86 | 277 | 249 | 154 | 39 | 326 | 202 | 437 | 454 | 350 | 274 | 294 | 220 | 100 |
| IDC#0478 | 2.3 | 17 | >500 | >500 | >500 | >500 | 336 | 331 | >500 | 145 | 431 | 125 | >500 | 156 | 50 |
| IDC#0481 | 2.3 | 308 | >500 | >500 | 479 | >500 | 372 | >500 | >500 | >500 | >500 | 132 | >500 | 292 | 33 |
| IDC#0490 | 2.1 | 70 | 418 | 381 | 107 | 364 | 412 | 459 | 347 | >500 | >500 | 39 | 461 | 235 | 83 |
| IDC#0508 | 2.1 | 35 | 85 | 81 | 58 | 162 | 185 | 415 | 134 | 374 | 314 | 117 | 397 | 150 | 100 |
| IDC#0513 | NA | NA | NA | NA | NA | NA | NA | NA | NA | NA | NA | NA | NA | NA | NA |
| IDC#0518 | 3.1 | 128 | 171 | 452 | 192 | >500 | 455 | >500 | >500 | 482 | >500 | 127 | >500 | 245 | 58 |
| IDC#0519 | NA | NA | NA | NA | NA | NA | NA | NA | NA | NA | NA | NA | NA | NA | NA |
| IDC#0526 | 1.7 | 18 | 498 | >500 | >500 | 150 | 71 | 55 | 122 | 138 | 461 | 125 | >500 | 120 | 75 |
| IDC#0527 | 2.3 | 168 | 152 | 389 | 215 | 79 | 120 | 109 | 83 | 254 | 269 | 144 | 149 | 159 | 100 |
| IDC#0529 | 1.9 | <3.9 | >500 | >500 | 157 | >500 | 32 | 449 | 314 | >500 | >500 | 206 | >500 | 91 | 50 |
| IDC#0539 | 2.7 | 223 | 348 | >500 | >500 | 167 | 490 | 222 | 455 | 475 | >500 | >500 | >500 | 315 | 58 |
| IDC#0543 | 2.0 | <3.9 | 52 | >500 | >500 | >500 | 353 | 96 | >500 | >500 | >500 | >500 | 388 | 77 | 42 |
| IDC#0546 | 2.0 | 18 | 72 | >500 | 146 | 407 | 117 | 368 | 218 | 192 | 458 | 62 | 284 | 154 | 92 |
| IDC#0549 | 2.1 | 84 | 209 | >500 | 89 | 322 | 99 | >500 | 381 | >500 | 349 | 46 | >500 | 153 | 67 |
| IDC#0561 | NA | NA | NA | NA | NA | NA | NA | NA | NA | NA | NA | NA | NA | NA | NA |
| IDC#0562 | 2.6 | 128 | 336 | 268 | 165 | >500 | >500 | 483 | 410 | >500 | >500 | 62 | 370 | 233 | 67 |
| IDC#0577 | 2.7 | 109 | 448 | >500 | 277 | >500 | 430 | 487 | >500 | >500 | >500 | 102 | >500 | 257 | 50 |
| IDC#0586 | 2.8 | 27 | 309 | >500 | 306 | >500 | >500 | 440 | >500 | >500 | >500 | 188 | 474 | 215 | 50 |
| IDC#0591 | 1.5 | 7 | 38 | >500 | 177 | >500 | 281 | 131 | 410 | >500 | 448 | 211 | 392 | 144 | 75 |
| IDC#0609 | 2.0 | 10 | 145 | >500 | 154 | 310 | 62 | 281 | 193 | 207 | 257 | 36 | 238 | 124 | 92 |
| IDC#0629 | NA | NA | NA | NA | NA | NA | NA | NA | NA | NA | NA | NA | NA | NA | NA |
| IDC#0638 | NA | NA | NA | NA | NA | NA | NA | NA | NA | NA | NA | NA | NA | NA | NA |
| IDC#0639 | NA | NA | NA | NA | NA | NA | NA | NA | NA | NA | NA | NA | NA | NA | NA |
| IDC#0654 | NA | NA | NA | NA | NA | NA | NA | NA | NA | NA | NA | NA | NA | NA | NA |
| IDC#0659 | NA | NA | NA | NA | NA | NA | NA | NA | NA | NA | NA | NA | NA | NA | NA |
| IDC#0666 | NA | NA | NA | NA | NA | NA | NA | NA | NA | NA | NA | NA | NA | NA | NA |
| IDC#0669 | NA | NA | NA | NA | NA | NA | NA | NA | NA | NA | NA | NA | NA | NA | NA |
| IDC#0686 | NA | NA | NA | NA | NA | NA | NA | NA | NA | NA | NA | NA | NA | NA | NA |
| IDC#0703 | NA | NA | NA | NA | NA | NA | NA | NA | NA | NA | NA | NA | NA | NA | NA |
| IDC#0706 | 1.8 | 188 | 284 | 396 | 81 | 209 | 212 | 434 | 302 | 226 | 284 | 64 | >500 | 213 | 92 |
| IDC#0707 | NA | NA | NA | NA | NA | NA | NA | NA | NA | NA | NA | NA | NA | NA | NA |
| IDC#0768 | 1.7 | 5 | >500 | 325 | 472 | 40 | 37 | 22 | 37 | 22 | 195 | 18 | >500 | 48 | 83 |
| IDC#0782 | 2.2 | 35 | 150 | >500 | 433 | >500 | 462 | 67 | 49 | 123 | >500 | 58 | 316 | 126 | 75 |
| IDC#0786 | 1.6 | 259 | 14 | 276 | 65 | >500 | >500 | 23 | 384 | >500 | >500 | 52 | 110 | 87 | 67 |
| IDC#0792 | 2.1 | 137 | >500 | 484 | >500 | >500 | >500 | 457 | >500 | >500 | 484 | 164 | >500 | 300 | 42 |
| IDC#0796 | NA | NA | NA | NA | NA | NA | NA | NA | NA | NA | NA | NA | NA | NA | NA |
| IDE#0057 | NA | NA | NA | NA | NA | NA | NA | NA | NA | NA | NA | NA | NA | NA | NA |
| IDE#0294 | NA | NA | NA | NA | NA | NA | NA | NA | NA | NA | NA | NA | NA | NA | NA |
| IDF#0033 | NA | NA | NA | NA | NA | NA | NA | NA | NA | NA | NA | NA | NA | NA | NA |

| IC50 (µg/ml) | Breadth |
|---|---|
| <25 | >95 |
| 25-100 | 80-95 |
| 100-300 | 65-79 |
| 300-500 | 50-64 |
| >500 | <50 |

**Supplementary Table 4.** IgG neutralizing activity of selected participants against the global panel.
Area under the curve (AUC) of neutralization curves resulting from IgG titra

**A)** Baseline visit

| ID | Δ to 1st timepoint (years) | AUC | | | | | | | | | | | | |
|---|---|---|---|---|---|---|---|---|---|---|---|---|---|---|
| | | 398F1 | 246F3 | CNE55 | CNE8 | X2278 | Tro11 | BJOX2000 | CH119 | CE1176 | CE0217 | 25710 | X1632 | Mean |
| IDC#0014 | 0 | 0,36 | 0,40 | 0,33 | 0,47 | 0,54 | 0,50 | 0,17 | 0,16 | 0,17 | 0,34 | 0,43 | 0,25 | 0,34 |
| IDC#0016 | 0 | 0,23 | 0,12 | 0,20 | 0,13 | 0,44 | 0,17 | 0,09 | 0,27 | 0,13 | 0,03 | 0,35 | 0,14 | 0,19 |
| IDC#0035 | 0 | 0,36 | 0,28 | 0,00 | 0,03 | 0,18 | 0,09 | 0,13 | 0,10 | 0,21 | 0,05 | 0,28 | 0,21 | 0,16 |
| IDC#0038 | 0 | 0,55 | 0,40 | 0,23 | 0,49 | 0,31 | 0,17 | 0,24 | 0,26 | 0,09 | 0,30 | 0,52 | 0,21 | 0,31 |
| IDC#0042 | 0 | 0,50 | 0,46 | 0,45 | 0,52 | 0,53 | 0,35 | 0,22 | 0,18 | 0,19 | 0,24 | 0,40 | 0,24 | 0,36 |
| IDC#0067 | 0 | 0,38 | 0,24 | 0,25 | 0,35 | 0,20 | 0,24 | 0,12 | 0,21 | 0,21 | 0,17 | 0,42 | 0,02 | 0,24 |
| IDC#0094 | 0 | 0,36 | 0,25 | 0,00 | 0,05 | 0,29 | 0,25 | 0,25 | 0,38 | 0,28 | 0,01 | 0,29 | 0,05 | 0,20 |
| IDC#0124 | 0 | 0,61 | 0,23 | 0,12 | 0,14 | 0,22 | 0,13 | 0,14 | 0,29 | 0,17 | 0,18 | 0,44 | 0,17 | 0,24 |
| IDC#0134 | 0 | 0,59 | 0,18 | 0,26 | 0,16 | 0,15 | 0,16 | 0,06 | 0,27 | 0,11 | 0,14 | 0,48 | 0,18 | 0,23 |
| IDC#0136 | 0 | 0,73 | 0,66 | 0,21 | 0,11 | 0,78 | 0,40 | 0,71 | 0,43 | 0,84 | 0,68 | 0,50 | 0,55 | 0,55 |
| IDC#0144 | 0 | 0,31 | 0,34 | 0,00 | 0,02 | 0,28 | 0,21 | 0,06 | 0,07 | 0,05 | 0,02 | 0,11 | 0,09 | 0,13 |
| IDC#0151 | 0 | 0,43 | 0,24 | 0,23 | 0,17 | 0,10 | 0,21 | 0,19 | 0,24 | 0,17 | 0,15 | 0,41 | 0,06 | 0,22 |
| IDC#0193 | 0 | 0,36 | 0,21 | 0,07 | 0,09 | 0,07 | 0,49 | 0,16 | 0,16 | 0,38 | 0,05 | 0,37 | 0,03 | 0,20 |
| IDC#0199 | 0 | 0,36 | 0,23 | 0,31 | 0,26 | 0,47 | 0,49 | 0,05 | 0,28 | 0,47 | 0,00 | 0,45 | 0,06 | 0,28 |
| IDC#0215 | 0 | 0,27 | 0,17 | 0,31 | 0,10 | 0,32 | 0,30 | 0,11 | 0,38 | 0,10 | 0,04 | 0,31 | 0,07 | 0,21 |
| IDC#0237 | 0 | 0,30 | 0,06 | 0,00 | 0,00 | 0,00 | 0,37 | 0,28 | 0,41 | 0,73 | 0,29 | 0,81 | 0,00 | 0,27 |
| IDC#0277 | 0 | 0,50 | 0,22 | 0,00 | 0,10 | 0,45 | 0,30 | 0,34 | 0,16 | 0,04 | 0,08 | 0,23 | 0,23 | 0,22 |
| IDC#0336 | 0 | 0,56 | 0,33 | 0,01 | 0,11 | 0,22 | 0,22 | 0,34 | 0,38 | 0,36 | 0,05 | 0,44 | 0,16 | 0,26 |
| IDC#0337 | 0 | 0,45 | 0,31 | 0,02 | 0,03 | 0,14 | 0,27 | 0,18 | 0,13 | 0,24 | 0,04 | 0,28 | 0,10 | 0,18 |
| IDC#0345 | 0 | 0,32 | 0,22 | 0,13 | 0,21 | 0,22 | 0,22 | 0,16 | 0,16 | 0,10 | 0,02 | 0,37 | 0,11 | 0,18 |
| IDC#0358 | 0 | 0,45 | 0,10 | 0,14 | 0,06 | 0,18 | 0,19 | 0,18 | 0,14 | 0,06 | 0,10 | 0,24 | 0,13 | 0,16 |
| IDC#0359 | 0 | 0,62 | 0,29 | 0,20 | 0,25 | 0,39 | 0,42 | 0,24 | 0,31 | 0,31 | 0,19 | 0,57 | 0,16 | 0,33 |
| IDC#0388 | 0 | 0,46 | 0,28 | 0,06 | 0,22 | 0,28 | 0,24 | 0,16 | 0,25 | 0,15 | 0,04 | 0,43 | 0,09 | 0,22 |
| IDC#0397 | 0 | 0,34 | 0,09 | 0,23 | 0,16 | 0,10 | 0,31 | 0,04 | 0,52 | 0,06 | 0,10 | 0,36 | 0,16 | 0,20 |
| IDC#0398 | 0 | 0,39 | 0,23 | 0,13 | 0,11 | 0,16 | 0,23 | 0,13 | 0,17 | 0,07 | 0,04 | 0,28 | 0,08 | 0,17 |
| IDC#0423 | 0 | 0,43 | 0,40 | 0,20 | 0,09 | 0,24 | 0,38 | 0,39 | 0,51 | 0,12 | 0,11 | 0,34 | 0,09 | 0,28 |
| IDC#0434 | 0 | 0,58 | 0,28 | 0,00 | 0,19 | 0,26 | 0,27 | 0,31 | 0,35 | 0,13 | 0,06 | 0,38 | 0,22 | 0,25 |
| IDC#0441 | 0 | 0,22 | 0,09 | 0,19 | 0,30 | 0,21 | 0,24 | 0,03 | 0,06 | 0,02 | 0,02 | 0,21 | 0,03 | 0,14 |
| IDC#0444 | 0 | 0,63 | 0,46 | 0,30 | 0,20 | 0,50 | 0,62 | 0,30 | 0,52 | 0,19 | 0,04 | 0,40 | 0,29 | 0,37 |
| IDC#0465 | 0 | 0,46 | 0,25 | 0,09 | 0,34 | 0,34 | 0,27 | 0,10 | 0,12 | 0,15 | 0,24 | 0,26 | 0,06 | 0,22 |
| IDC#0468 | 0 | 0,70 | 0,25 | 0,01 | 0,46 | 0,26 | 0,28 | 0,34 | 0,41 | 0,12 | 0,03 | 0,45 | 0,11 | 0,28 |
| IDC#0476 | 0 | 0,37 | 0,18 | 0,19 | 0,08 | 0,48 | 0,22 | 0,12 | 0,08 | 0,14 | 0,13 | 0,27 | 0,27 | 0,21 |
| IDC#0478 | 0 | 0,44 | 0,08 | 0,00 | 0,09 | 0,20 | 0,27 | 0,12 | 0,09 | 0,32 | 0,13 | 0,47 | 0,00 | 0,18 |
| IDC#0481 | 0 | 0,32 | 0,16 | 0,21 | 0,08 | 0,49 | 0,27 | 0,02 | 0,07 | 0,27 | 0,28 | 0,41 | 0,11 | 0,23 |
| IDC#0490 | 0 | 0,31 | 0,20 | 0,24 | 0,34 | 0,24 | 0,31 | 0,17 | 0,36 | 0,24 | 0,14 | 0,59 | 0,23 | 0,28 |
| IDC#0508 | 0 | 0,49 | 0,50 | 0,56 | 0,57 | 0,35 | 0,44 | 0,13 | 0,44 | 0,24 | 0,30 | 0,40 | 0,32 | 0,40 |
| IDC#0513 | 0 | 0,39 | 0,18 | 0,38 | 0,40 | 0,45 | 0,50 | 0,24 | 0,32 | 0,21 | 0,06 | 0,42 | 0,28 | 0,32 |
| IDC#0518 | 0 | 0,54 | 0,47 | 0,34 | 0,51 | 0,28 | 0,37 | 0,26 | 0,30 | 0,29 | 0,05 | 0,56 | 0,24 | 0,35 |
| IDC#0519 | 0 | 0,53 | 0,36 | 0,21 | 0,04 | 0,44 | 0,33 | 0,26 | 0,33 | 0,18 | 0,27 | 0,47 | 0,22 | 0,30 |
| IDC#0526 | 0 | 0,49 | 0,14 | 0,00 | 0,00 | 0,26 | 0,40 | 0,41 | 0,33 | 0,21 | 0,11 | 0,25 | 0,07 | 0,22 |
| IDC#0527 | 0 | 0,31 | 0,36 | 0,24 | 0,28 | 0,39 | 0,29 | 0,28 | 0,50 | 0,19 | 0,14 | 0,27 | 0,32 | 0,30 |
| IDC#0529 | 0 | 0,76 | 0,13 | 0,07 | 0,07 | 0,17 | 0,41 | 0,12 | 0,20 | 0,06 | 0,02 | 0,22 | 0,00 | 0,19 |
| IDC#0539 | 0 | 0,40 | 0,19 | 0,01 | 0,03 | 0,35 | 0,18 | 0,28 | 0,21 | 0,15 | 0,05 | 0,18 | 0,00 | 0,17 |
| IDC#0543 | 0 | 0,82 | 0,53 | 0,13 | 0,01 | 0,05 | 0,35 | 0,53 | 0,31 | 0,21 | 0,03 | 0,26 | 0,28 | 0,29 |
| IDC#0546 | 0 | 0,63 | 0,37 | 0,20 | 0,13 | 0,18 | 0,26 | 0,24 | 0,28 | 0,22 | 0,10 | 0,45 | 0,22 | 0,27 |
| IDC#0549 | 0 | 0,57 | 0,12 | 0,12 | 0,08 | 0,13 | 0,11 | 0,09 | 0,26 | 0,11 | 0,09 | 0,41 | 0,13 | 0,18 |
| IDC#0561 | 0 | 0,43 | 0,33 | 0,42 | 0,37 | 0,56 | 0,53 | 0,39 | 0,28 | 0,12 | 0,27 | 0,44 | 0,31 | 0,37 |
| IDC#0562 | 0 | 0,53 | 0,37 | 0,30 | 0,43 | 0,23 | 0,25 | 0,33 | 0,32 | 0,14 | 0,03 | 0,62 | 0,30 | 0,32 |
| IDC#0577 | 0 | 0,46 | 0,18 | 0,19 | 0,21 | 0,28 | 0,26 | 0,23 | 0,18 | 0,06 | 0,04 | 0,43 | 0,17 | 0,22 |
| IDC#0586 | 0 | 0,52 | 0,16 | 0,08 | 0,10 | 0,08 | 0,04 | 0,11 | 0,11 | 0,02 | 0,01 | 0,31 | 0,10 | 0,14 |
| IDC#0591 | 0 | 0,62 | 0,48 | 0,25 | 0,19 | 0,24 | 0,25 | 0,34 | 0,19 | 0,05 | 0,13 | 0,32 | 0,27 | 0,28 |
| IDC#0609 | 0 | 0,67 | 0,29 | 0,08 | 0,20 | 0,23 | 0,44 | 0,25 | 0,34 | 0,32 | 0,24 | 0,51 | 0,24 | 0,32 |
| IDC#0629 | 0 | 0,64 | 0,28 | 0,18 | 0,23 | 0,10 | 0,20 | 0,18 | 0,27 | 0,21 | 0,17 | 0,34 | 0,20 | 0,25 |
| IDC#0638 | 0 | 0,29 | 0,21 | 0,14 | 0,36 | 0,15 | 0,30 | 0,14 | 0,13 | 0,05 | 0,01 | 0,26 | 0,33 | 0,20 |
| IDC#0639 | 0 | 0,43 | 0,13 | 0,07 | 0,10 | 0,00 | 0,10 | 0,20 | 0,22 | 0,21 | 0,00 | 0,35 | 0,23 | 0,17 |
| IDC#0654 | 0 | 0,59 | 0,26 | 0,21 | 0,09 | 0,18 | 0,26 | 0,11 | 0,09 | 0,04 | 0,02 | 0,34 | 0,14 | 0,19 |
| IDC#0659 | 0 | 0,50 | 0,31 | 0,19 | 0,45 | 0,52 | 0,39 | 0,30 | 0,17 | 0,21 | 0,33 | 0,56 | 0,15 | 0,34 |
| IDC#0666 | 0 | 0,73 | 0,18 | 0,00 | 0,05 | 0,66 | 0,75 | 0,72 | 0,52 | 0,52 | 0,07 | 0,16 | 0,08 | 0,37 |
| IDC#0669 | 0 | 0,56 | 0,65 | 0,00 | 0,15 | 0,17 | 0,27 | 0,41 | 0,39 | 0,34 | 0,31 | 0,16 | 0,57 | 0,33 |
| IDC#0686 | 0 | 0,43 | 0,27 | 0,13 | 0,12 | 0,24 | 0,46 | 0,14 | 0,07 | 0,06 | 0,21 | 0,42 | 0,16 | 0,22 |
| IDC#0703 | 0 | 0,26 | 0,23 | 0,26 | 0,35 | 0,35 | 0,16 | 0,09 | 0,13 | 0,11 | 0,30 | 0,45 | 0,21 | 0,24 |
| IDC#0706 | 0 | 0,38 | 0,24 | 0,17 | 0,25 | 0,21 | 0,16 | 0,02 | 0,17 | 0,13 | 0,31 | 0,42 | 0,16 | 0,22 |
| IDC#0707 | 0 | 0,86 | 0,49 | 0,05 | 0,30 | 0,39 | 0,56 | 0,50 | 0,25 | 0,11 | 0,08 | 0,39 | 0,10 | 0,34 |
| IDC#0768 | 0 | 0,69 | 0,12 | 0,24 | 0,02 | 0,46 | 0,45 | 0,46 | 0,39 | 0,51 | 0,19 | 0,59 | 0,11 | 0,35 |
| IDC#0782 | 0 | 0,64 | 0,46 | 0,15 | 0,31 | 0,24 | 0,26 | 0,56 | 0,60 | 0,46 | 0,14 | 0,63 | 0,27 | 0,39 |
| IDC#0786 | 0 | 0,37 | 0,77 | 0,82 | 0,52 | 0,12 | 0,05 | 0,78 | 0,37 | 0,09 | 0,07 | 0,66 | 0,58 | 0,43 |
| IDC#0792 | 0 | 0,41 | 0,11 | 0,29 | 0,02 | 0,23 | 0,17 | 0,16 | 0,12 | 0,08 | 0,17 | 0,46 | 0,05 | 0,19 |
| IDC#0796 | 0 | 0,54 | 0,35 | 0,43 | 0,27 | 0,48 | 0,33 | 0,25 | 0,50 | 0,24 | 0,13 | 0,61 | 0,24 | 0,36 |
| IDE#0057 | 0 | 0,47 | 0,43 | 0,33 | 0,55 | 0,45 | 0,38 | 0,40 | 0,33 | 0,32 | 0,21 | 0,58 | 0,30 | 0,40 |
| IDE#0294 | 0 | 0,81 | 0,52 | 0,33 | 0,56 | 0,67 | 0,60 | 0,50 | 0,46 | 0,38 | 0,28 | 0,76 | 0,56 | 0,54 |
| IDF#0033 | 0 | 0,56 | 0,50 | 0,24 | 0,48 | 0,52 | 0,58 | 0,29 | 0,14 | 0,42 | 0,43 | 0,52 | 0,35 | 0,42 |

| AUC |
|---|
| >0.5 |
| 0.4-0.5 |
| 0.3-0.39 |
| 0.15-0.29 |
| <0.2 |

**Supplementary Table 4.** IgG neutralizing activity of selected participants against the global panel.
Area under the curve (AUC) of neutralization curves resulting from IgG titra

**B)** $2^{nd}$ visit

| ID | Δ to 1st timepoint (years) | AUC 398F1 | 246F3 | CNE55 | CNE8 | X2278 | Tro11 | BJOX2000 | CH119 | CE1176 | CE0217 | 25710 | X1632 | Mean |
|---|---|---|---|---|---|---|---|---|---|---|---|---|---|---|
| IDC#0014 | 1.5 | 0,35 | 0,37 | 0,35 | 0,48 | 0,54 | 0,45 | 0,18 | 0,17 | 0,17 | 0,29 | 0,41 | 0,26 | 0,33 |
| IDC#0016 | 1.5 | 0,16 | 0,07 | 0,17 | 0,08 | 0,39 | 0,12 | 0,05 | 0,16 | 0,08 | 0,02 | 0,26 | 0,09 | 0,14 |
| IDC#0035 | 1.5 | 0,35 | 0,24 | 0,18 | 0,00 | 0,14 | 0,09 | 0,17 | 0,16 | 0,19 | 0,04 | 0,35 | 0,19 | 0,17 |
| IDC#0038 | 1.6 | 0,53 | 0,39 | 0,24 | 0,43 | 0,27 | 0,22 | 0,23 | 0,27 | 0,13 | 0,34 | 0,48 | 0,18 | 0,31 |
| IDC#0042 | 1.5 | 0,52 | 0,26 | 0,24 | 0,21 | 0,32 | 0,16 | 0,06 | 0,05 | 0,06 | 0,08 | 0,27 | 0,09 | 0,19 |
| IDC#0067 | 2.6 | 0,32 | 0,16 | 0,00 | 0,15 | 0,14 | 0,07 | 0,10 | 0,09 | 0,08 | 0,11 | 0,28 | 0,00 | 0,13 |
| IDC#0094 | 3.4 | 0,37 | 0,26 | 0,02 | 0,11 | 0,34 | 0,26 | 0,30 | 0,35 | 0,31 | 0,07 | 0,34 | 0,08 | 0,24 |
| IDC#0124 | 1.5 | 0,59 | 0,16 | 0,07 | 0,01 | 0,12 | 0,02 | 0,10 | 0,32 | 0,14 | 0,03 | 0,44 | 0,07 | 0,17 |
| IDC#0134 | 1.4 | 0,58 | 0,16 | 0,15 | 0,12 | 0,16 | 0,13 | 0,05 | 0,21 | 0,09 | 0,10 | 0,45 | 0,16 | 0,20 |
| IDC#0136 | 0.9 | 0,67 | 0,52 | 0,22 | 0,15 | 0,69 | 0,40 | 0,60 | 0,41 | 0,70 | 0,55 | 0,43 | 0,48 | 0,48 |
| IDC#0144 | 1.5 | 0,19 | 0,38 | 0,00 | 0,06 | 0,28 | 0,18 | 0,06 | 0,04 | 0,07 | 0,03 | 0,06 | 0,07 | 0,12 |
| IDC#0151 | 1.5 | 0,41 | 0,24 | 0,18 | 0,08 | 0,07 | 0,16 | 0,13 | 0,18 | 0,15 | 0,09 | 0,38 | 0,07 | 0,18 |
| IDC#0193 | 1.3 | 0,30 | 0,14 | 0,00 | 0,08 | 0,05 | 0,40 | 0,11 | 0,07 | 0,34 | 0,02 | 0,29 | 0,02 | 0,15 |
| IDC#0199 | 2.1 | 0,33 | 0,13 | 0,24 | 0,16 | 0,41 | 0,39 | 0,03 | 0,26 | 0,43 | 0,00 | 0,29 | 0,00 | 0,22 |
| IDC#0215 | 1.4 | 0,29 | 0,14 | 0,29 | 0,09 | 0,18 | 0,26 | 0,11 | 0,37 | 0,12 | 0,06 | 0,33 | 0,06 | 0,19 |
| IDC#0237 | 1.5 | 0,34 | 0,05 | 0,00 | 0,00 | 0,00 | 0,37 | 0,32 | 0,40 | 0,72 | 0,29 | 0,79 | 0,00 | 0,27 |
| IDC#0277 | 1.9 | 0,46 | 0,21 | 0,00 | 0,09 | 0,34 | 0,22 | 0,29 | 0,09 | 0,04 | 0,03 | 0,17 | 0,19 | 0,18 |
| IDC#0336 | 2.9 | 0,54 | 0,31 | 0,16 | 0,13 | 0,22 | 0,25 | 0,33 | 0,37 | 0,37 | 0,07 | 0,41 | 0,22 | 0,28 |
| IDC#0337 | 1.4 | 0,46 | 0,27 | 0,00 | 0,01 | 0,06 | 0,25 | 0,11 | 0,06 | 0,27 | 0,05 | 0,24 | 0,07 | 0,15 |
| IDC#0345 | 3.2 | 0,26 | 0,19 | 0,13 | 0,11 | 0,16 | 0,16 | 0,13 | 0,15 | 0,11 | 0,00 | 0,26 | 0,05 | 0,14 |
| IDC#0358 | 1.4 | 0,48 | 0,12 | 0,16 | 0,03 | 0,18 | 0,21 | 0,20 | 0,14 | 0,06 | 0,12 | 0,24 | 0,13 | 0,17 |
| IDC#0359 | 2.9 | 0,69 | 0,39 | 0,22 | 0,29 | 0,51 | 0,52 | 0,27 | 0,46 | 0,29 | 0,17 | 0,55 | 0,32 | 0,39 |
| IDC#0388 | 1.5 | 0,41 | 0,22 | 0,01 | 0,16 | 0,22 | 0,21 | 0,10 | 0,21 | 0,08 | 0,01 | 0,39 | 0,08 | 0,17 |
| IDC#0397 | 1.2 | 0,36 | 0,08 | 0,22 | 0,14 | 0,15 | 0,29 | 0,06 | 0,49 | 0,05 | 0,11 | 0,38 | 0,14 | 0,21 |
| IDC#0398 | 1.5 | 0,33 | 0,19 | 0,00 | 0,07 | 0,12 | 0,18 | 0,09 | 0,11 | 0,02 | 0,02 | 0,20 | 0,02 | 0,11 |
| IDC#0423 | 1.9 | 0,42 | 0,20 | 0,07 | 0,03 | 0,18 | 0,31 | 0,28 | 0,34 | 0,13 | 0,01 | 0,28 | 0,07 | 0,19 |
| IDC#0434 | 2.8 | 0,55 | 0,25 | 0,09 | 0,14 | 0,27 | 0,25 | 0,29 | 0,28 | 0,17 | 0,05 | 0,35 | 0,22 | 0,24 |
| IDC#0441 | 1.5 | 0,22 | 0,06 | 0,00 | 0,43 | 0,16 | 0,24 | 0,03 | 0,05 | 0,04 | 0,02 | 0,18 | 0,00 | 0,12 |
| IDC#0444 | 1.6 | 0,46 | 0,42 | 0,27 | 0,38 | 0,46 | 0,59 | 0,34 | 0,45 | 0,19 | 0,02 | 0,42 | 0,24 | 0,35 |
| IDC#0465 | 2.1 | 0,38 | 0,17 | 0,01 | 0,20 | 0,15 | 0,13 | 0,01 | 0,03 | 0,06 | 0,10 | 0,17 | 0,02 | 0,12 |
| IDC#0468 | 1.1 | 0,55 | 0,18 | 0,00 | 0,27 | 0,17 | 0,14 | 0,24 | 0,27 | 0,06 | 0,01 | 0,32 | 0,08 | 0,19 |
| IDC#0476 | 1.6 | 0,27 | 0,21 | 0,22 | 0,17 | 0,47 | 0,28 | 0,15 | 0,08 | 0,15 | 0,13 | 0,34 | 0,24 | 0,23 |
| IDC#0478 | 1.5 | 0,51 | 0,08 | 0,00 | 0,01 | 0,20 | 0,28 | 0,10 | 0,07 | 0,35 | 0,11 | 0,43 | 0,00 | 0,18 |
| IDC#0481 | 1.1 | 0,33 | 0,05 | 0,09 | 0,02 | 0,20 | 0,10 | 0,02 | 0,04 | 0,10 | 0,03 | 0,28 | 0,02 | 0,11 |
| IDC#0490 | 1.2 | 0,33 | 0,11 | 0,14 | 0,10 | 0,14 | 0,19 | 0,04 | 0,18 | 0,08 | 0,07 | 0,41 | 0,19 | 0,17 |
| IDC#0508 | 1.2 | 0,62 | 0,50 | 0,63 | 0,53 | 0,45 | 0,56 | 0,15 | 0,42 | 0,25 | 0,32 | 0,45 | 0,35 | 0,44 |
| IDC#0513 | 0.9 | 0,44 | 0,19 | 0,30 | 0,19 | 0,34 | 0,40 | 0,10 | 0,25 | 0,16 | 0,03 | 0,34 | 0,23 | 0,25 |
| IDC#0518 | 2.2 | 0,45 | 0,35 | 0,22 | 0,29 | 0,16 | 0,13 | 0,16 | 0,20 | 0,17 | 0,00 | 0,32 | 0,19 | 0,22 |
| IDC#0519 | 1.2 | 0,50 | 0,31 | 0,19 | 0,00 | 0,35 | 0,29 | 0,21 | 0,29 | 0,14 | 0,20 | 0,43 | 0,17 | 0,26 |
| IDC#0526 | 1.4 | 0,64 | 0,17 | 0,02 | 0,01 | 0,30 | 0,46 | 0,47 | 0,42 | 0,35 | 0,19 | 0,30 | 0,10 | 0,29 |
| IDC#0527 | 1.0 | 0,39 | 0,40 | 0,27 | 0,32 | 0,48 | 0,34 | 0,37 | 0,51 | 0,30 | 0,31 | 0,39 | 0,37 | 0,37 |
| IDC#0529 | 1.5 | 0,76 | 0,13 | 0,10 | 0,13 | 0,12 | 0,42 | 0,17 | 0,20 | 0,06 | 0,01 | 0,24 | 0,00 | 0,20 |
| IDC#0539 | 1.4 | 0,40 | 0,19 | 0,06 | 0,04 | 0,34 | 0,17 | 0,26 | 0,20 | 0,11 | 0,05 | 0,17 | 0,00 | 0,17 |
| IDC#0543 | 1.0 | 0,77 | 0,51 | 0,07 | 0,05 | 0,00 | 0,24 | 0,42 | 0,27 | 0,18 | 0,02 | 0,22 | 0,23 | 0,25 |
| IDC#0546 | 1.0 | 0,65 | 0,41 | 0,22 | 0,15 | 0,18 | 0,30 | 0,24 | 0,29 | 0,23 | 0,15 | 0,47 | 0,26 | 0,30 |
| IDC#0549 | 1.7 | 0,57 | 0,22 | 0,24 | 0,14 | 0,25 | 0,20 | 0,11 | 0,22 | 0,15 | 0,18 | 0,42 | 0,13 | 0,24 |
| IDC#0561 | 1.1 | 0,37 | 0,26 | 0,40 | 0,31 | 0,50 | 0,49 | 0,35 | 0,28 | 0,07 | 0,20 | 0,39 | 0,28 | 0,33 |
| IDC#0562 | 1.9 | 0,42 | 0,28 | 0,25 | 0,25 | 0,16 | 0,13 | 0,19 | 0,21 | 0,05 | 0,01 | 0,46 | 0,21 | 0,22 |
| IDC#0577 | 2.0 | 0,44 | 0,12 | 0,10 | 0,17 | 0,20 | 0,20 | 0,20 | 0,15 | 0,05 | 0,01 | 0,35 | 0,10 | 0,17 |
| IDC#0586 | 1.2 | 0,50 | 0,15 | 0,12 | 0,08 | 0,11 | 0,09 | 0,11 | 0,11 | 0,05 | 0,01 | 0,30 | 0,09 | 0,14 |
| IDC#0591 | 0.8 | 0,71 | 0,50 | 0,24 | 0,18 | 0,14 | 0,23 | 0,33 | 0,20 | 0,13 | 0,12 | 0,31 | 0,21 | 0,28 |
| IDC#0609 | 0.8 | 0,71 | 0,31 | 0,08 | 0,18 | 0,25 | 0,52 | 0,29 | 0,36 | 0,35 | 0,29 | 0,57 | 0,22 | 0,34 |
| IDC#0629 | 1.0 | 0,59 | 0,32 | 0,16 | 0,22 | 0,16 | 0,22 | 0,10 | 0,20 | 0,12 | 0,14 | 0,27 | 0,14 | 0,22 |
| IDC#0638 | 1.0 | 0,38 | 0,16 | 0,09 | 0,15 | 0,00 | 0,20 | 0,10 | 0,15 | 0,08 | 0,02 | 0,22 | 0,31 | 0,16 |
| IDC#0639 | 1.0 | 0,32 | 0,16 | 0,15 | 0,03 | 0,20 | 0,16 | 0,17 | 0,22 | 0,19 | 0,02 | 0,37 | 0,25 | 0,19 |
| IDC#0654 | 0.9 | 0,58 | 0,20 | 0,14 | 0,09 | 0,17 | 0,25 | 0,07 | 0,09 | 0,05 | 0,02 | 0,30 | 0,12 | 0,17 |
| IDC#0659 | 2.8 | 0,45 | 0,26 | 0,17 | 0,33 | 0,45 | 0,29 | 0,20 | 0,15 | 0,14 | 0,26 | 0,52 | 0,08 | 0,27 |
| IDC#0666 | 0.8 | 0,77 | 0,22 | 0,01 | 0,03 | 0,68 | 0,76 | 0,73 | 0,54 | 0,56 | 0,06 | 0,18 | 0,08 | 0,38 |
| IDC#0669 | 0.9 | 0,62 | 0,63 | 0,08 | 0,10 | 0,12 | 0,25 | 0,38 | 0,37 | 0,30 | 0,20 | 0,23 | 0,56 | 0,32 |
| IDC#0686 | 1.0 | 0,41 | 0,28 | 0,12 | 0,13 | 0,23 | 0,41 | 0,12 | 0,06 | 0,04 | 0,18 | 0,43 | 0,19 | 0,22 |
| IDC#0703 | 0.9 | 0,39 | 0,18 | 0,16 | 0,17 | 0,12 | 0,15 | 0,09 | 0,17 | 0,17 | 0,05 | 0,27 | 0,18 | 0,18 |
| IDC#0706 | 0.7 | 0,33 | 0,19 | 0,14 | 0,27 | 0,23 | 0,20 | 0,06 | 0,23 | 0,14 | 0,32 | 0,41 | 0,16 | 0,22 |
| IDC#0707 | 2.7 | 0,71 | 0,40 | 0,04 | 0,18 | 0,27 | 0,40 | 0,32 | 0,18 | 0,09 | 0,05 | 0,31 | 0,06 | 0,25 |
| IDC#0768 | 0.8 | 0,70 | 0,04 | 0,21 | 0,01 | 0,47 | 0,49 | 0,53 | 0,42 | 0,57 | 0,15 | 0,63 | 0,01 | 0,35 |
| IDC#0782 | 1.6 | 0,60 | 0,36 | 0,11 | 0,22 | 0,20 | 0,10 | 0,46 | 0,49 | 0,34 | 0,07 | 0,47 | 0,27 | 0,31 |
| IDC#0786 | 0.5 | 0,35 | 0,66 | 0,71 | 0,37 | 0,11 | 0,00 | 0,65 | 0,21 | 0,07 | 0,00 | 0,59 | 0,48 | 0,35 |
| IDC#0792 | 0.6 | 0,39 | 0,04 | 0,13 | 0,01 | 0,16 | 0,10 | 0,10 | 0,07 | 0,06 | 0,09 | 0,37 | 0,00 | 0,13 |
| IDC#0796 | 1.3 | 0,49 | 0,29 | 0,34 | 0,23 | 0,33 | 0,25 | 0,22 | 0,38 | 0,18 | 0,06 | 0,46 | 0,20 | 0,29 |
| IDE#0057 | 4.0 | 0,38 | 0,21 | 0,10 | 0,19 | 0,22 | 0,17 | 0,19 | 0,15 | 0,19 | 0,02 | 0,32 | 0,14 | 0,19 |
| IDE#0294 | 3.0 | 0,75 | 0,44 | 0,21 | 0,50 | 0,58 | 0,49 | 0,43 | 0,43 | 0,29 | 0,16 | 0,68 | 0,47 | 0,45 |
| IDF#0033 | 2.8 | 0,64 | 0,55 | 0,46 | 0,42 | 0,53 | 0,66 | 0,39 | 0,30 | 0,47 | 0,44 | 0,46 | 0,36 | 0,47 |

| AUC |
|---|
| >0.5 |
| 0.4-0.5 |
| 0.3-0.39 |
| 0.15-0.29 |
| |

**Supplementary Table 4.** IgG neutralizing activity of selected participants against the global panel.
Area under the curve (AUC) of neutralization curves resulting from IgG titra

**C)** 3rd visit

| ID | Δ to 1st timepoint (years) | AUC: 398F1 | 246F3 | CNE55 | CNE8 | X2278 | Tro11 | BJOX2000 | CH119 | CE1176 | CE0217 | 25710 | X1632 | Mean |
|---|---|---|---|---|---|---|---|---|---|---|---|---|---|---|
| IDC#0014 | NA | NA | NA | NA | NA | NA | NA | NA | NA | NA | NA | NA | NA | NA |
| IDC#0016 | 3.3 | 0,33 | 0,08 | 0,07 | 0,09 | 0,28 | 0,08 | 0,06 | 0,25 | 0,08 | 0,02 | 0,22 | 0,06 | 0,14 |
| IDC#0035 | 3.0 | 0,53 | 0,18 | 0,06 | 0,19 | 0,06 | 0,06 | 0,13 | 0,18 | 0,16 | 0,01 | 0,29 | 0,12 | 0,16 |
| IDC#0038 | 2.6 | 0,62 | 0,34 | 0,00 | 0,32 | 0,19 | 0,20 | 0,17 | 0,00 | 0,00 | 0,00 | 0,00 | 0,16 | 0,17 |
| IDC#0042 | 2.9 | 0,47 | 0,28 | 0,15 | 0,27 | 0,39 | 0,16 | 0,07 | 0,02 | 0,02 | 0,09 | 0,23 | 0,16 | 0,19 |
| IDC#0067 | 3.2 | 0,32 | 0,18 | 0,11 | 0,18 | 0,18 | 0,10 | 0,09 | 0,12 | 0,07 | 0,03 | 0,30 | 0,03 | 0,14 |
| IDC#0094 | NA | NA | NA | NA | NA | NA | NA | NA | NA | NA | NA | NA | NA | NA |
| IDC#0124 | 2.9 | 0,46 | 0,23 | 0,01 | 0,13 | 0,17 | 0,11 | 0,20 | 0,33 | 0,24 | 0,08 | 0,47 | 0,21 | 0,22 |
| IDC#0134 | 2.7 | 0,50 | 0,17 | 0,07 | 0,21 | 0,11 | 0,13 | 0,07 | 0,19 | 0,13 | 0,08 | 0,41 | 0,13 | 0,18 |
| IDC#0136 | 1.4 | 0,67 | 0,40 | 0,20 | 0,29 | 0,53 | 0,20 | 0,46 | 0,29 | 0,59 | 0,42 | 0,34 | 0,40 | 0,40 |
| IDC#0144 | 2.5 | 0,38 | 0,37 | 0,00 | 0,08 | 0,44 | 0,25 | 0,17 | 0,10 | 0,04 | 0,02 | 0,14 | 0,14 | 0,18 |
| IDC#0151 | 2.5 | 0,66 | 0,15 | 0,17 | 0,12 | 0,15 | 0,23 | 0,23 | 0,27 | 0,22 | 0,11 | 0,41 | 0,10 | 0,24 |
| IDC#0193 | NA | NA | NA | NA | NA | NA | NA | NA | NA | NA | NA | NA | NA | NA |
| IDC#0199 | NA | NA | NA | NA | NA | NA | NA | NA | NA | NA | NA | NA | NA | NA |
| IDC#0215 | NA | NA | NA | NA | NA | NA | NA | NA | NA | NA | NA | NA | NA | NA |
| IDC#0237 | 2.5 | 0,48 | 0,06 | 0,00 | 0,13 | 0,00 | 0,42 | 0,36 | 0,41 | 0,73 | 0,28 | 0,82 | 0,01 | 0,31 |
| IDC#0277 | 2.3 | 0,43 | 0,22 | 0,00 | 0,12 | 0,39 | 0,25 | 0,29 | 0,09 | 0,08 | 0,02 | 0,19 | 0,20 | 0,19 |
| IDC#0336 | NA | NA | NA | NA | NA | NA | NA | NA | NA | NA | NA | NA | NA | NA |
| IDC#0337 | 2.4 | 0,54 | 0,41 | 0,01 | 0,06 | 0,14 | 0,31 | 0,21 | 0,08 | 0,34 | 0,08 | 0,29 | 0,17 | 0,22 |
| IDC#0345 | NA | NA | NA | NA | NA | NA | NA | NA | NA | NA | NA | NA | NA | NA |
| IDC#0358 | NA | NA | NA | NA | NA | NA | NA | NA | NA | NA | NA | NA | NA | NA |
| IDC#0359 | NA | NA | NA | NA | NA | NA | NA | NA | NA | NA | NA | NA | NA | NA |
| IDC#0388 | 1.9 | 0,46 | 0,34 | 0,00 | 0,26 | 0,40 | 0,18 | 0,25 | 0,27 | 0,13 | 0,02 | 0,44 | 0,10 | 0,24 |
| IDC#0397 | 2.3 | 0,34 | 0,11 | 0,18 | 0,16 | 0,17 | 0,22 | 0,11 | 0,53 | 0,02 | 0,07 | 0,35 | 0,14 | 0,20 |
| IDC#0398 | 2.2 | 0,51 | 0,47 | 0,19 | 0,22 | 0,36 | 0,23 | 0,25 | 0,23 | 0,17 | 0,11 | 0,36 | 0,22 | 0,28 |
| IDC#0423 | 2.4 | 0,44 | 0,21 | 0,00 | 0,09 | 0,20 | 0,19 | 0,32 | 0,38 | 0,06 | 0,00 | 0,28 | 0,08 | 0,19 |
| IDC#0434 | NA | NA | NA | NA | NA | NA | NA | NA | NA | NA | NA | NA | NA | NA |
| IDC#0441 | 2.2 | 0,32 | 0,29 | 0,12 | 0,42 | 0,24 | 0,18 | 0,15 | 0,17 | 0,13 | 0,03 | 0,25 | 0,10 | 0,20 |
| IDC#0444 | 2.2 | 0,69 | 0,41 | 0,25 | 0,23 | 0,59 | 0,53 | 0,36 | 0,50 | 0,18 | 0,05 | 0,36 | 0,30 | 0,37 |
| IDC#0465 | 3.0 | 0,39 | 0,16 | 0,06 | 0,24 | 0,19 | 0,14 | 0,02 | 0,09 | 0,11 | 0,12 | 0,20 | 0,00 | 0,14 |
| IDC#0468 | 2.3 | 0,31 | 0,14 | 0,00 | 0,12 | 0,16 | 0,11 | 0,16 | 0,14 | 0,01 | 0,01 | 0,20 | 0,05 | 0,12 |
| IDC#0476 | 2.8 | 0,44 | 0,30 | 0,32 | 0,32 | 0,59 | 0,20 | 0,24 | 0,17 | 0,13 | 0,19 | 0,30 | 0,24 | 0,29 |
| IDC#0478 | 2.3 | 0,70 | 0,15 | 0,01 | 0,01 | 0,20 | 0,21 | 0,15 | 0,18 | 0,30 | 0,12 | 0,36 | 0,00 | 0,20 |
| IDC#0481 | 2.3 | 0,40 | 0,11 | 0,20 | 0,20 | 0,25 | 0,18 | 0,05 | 0,07 | 0,12 | 0,09 | 0,35 | 0,00 | 0,17 |
| IDC#0490 | 2.1 | 0,44 | 0,07 | 0,14 | 0,25 | 0,26 | 0,13 | 0,19 | 0,24 | 0,08 | 0,02 | 0,45 | 0,21 | 0,21 |
| IDC#0508 | 2.1 | 0,52 | 0,41 | 0,38 | 0,44 | 0,31 | 0,22 | 0,18 | 0,32 | 0,14 | 0,09 | 0,34 | 0,14 | 0,29 |
| IDC#0513 | NA | NA | NA | NA | NA | NA | NA | NA | NA | NA | NA | NA | NA | NA |
| IDC#0518 | 3.1 | 0,41 | 0,28 | 0,19 | 0,24 | 0,13 | 0,10 | 0,15 | 0,16 | 0,14 | 0,00 | 0,33 | 0,12 | 0,19 |
| IDC#0519 | NA | NA | NA | NA | NA | NA | NA | NA | NA | NA | NA | NA | NA | NA |
| IDC#0526 | 1.7 | 0,67 | 0,10 | 0,00 | 0,08 | 0,37 | 0,45 | 0,47 | 0,35 | 0,31 | 0,10 | 0,32 | 0,02 | 0,27 |
| IDC#0527 | 2.3 | 0,27 | 0,26 | 0,05 | 0,20 | 0,39 | 0,34 | 0,31 | 0,39 | 0,20 | 0,16 | 0,31 | 0,28 | 0,26 |
| IDC#0529 | 1.9 | 0,82 | 0,03 | 0,00 | 0,17 | 0,05 | 0,42 | 0,15 | 0,13 | 0,01 | 0,02 | 0,19 | 0,00 | 0,17 |
| IDC#0539 | 2.7 | 0,42 | 0,23 | 0,00 | 0,09 | 0,30 | 0,24 | 0,27 | 0,21 | 0,13 | 0,06 | 0,24 | 0,05 | 0,19 |
| IDC#0543 | 2.0 | 0,83 | 0,50 | 0,00 | 0,07 | 0,00 | 0,13 | 0,33 | 0,13 | 0,11 | 0,00 | 0,16 | 0,15 | 0,20 |
| IDC#0546 | 2.0 | 0,68 | 0,43 | 0,12 | 0,27 | 0,16 | 0,33 | 0,22 | 0,25 | 0,23 | 0,12 | 0,48 | 0,24 | 0,29 |
| IDC#0549 | 2.1 | 0,49 | 0,33 | 0,21 | 0,39 | 0,32 | 0,37 | 0,24 | 0,26 | 0,17 | 0,23 | 0,51 | 0,13 | 0,30 |
| IDC#0561 | NA | NA | NA | NA | NA | NA | NA | NA | NA | NA | NA | NA | NA | NA |
| IDC#0562 | 2.6 | 0,41 | 0,29 | 0,27 | 0,32 | 0,20 | 0,14 | 0,22 | 0,18 | 0,08 | 0,02 | 0,47 | 0,24 | 0,24 |
| IDC#0577 | 2.7 | 0,48 | 0,15 | 0,10 | 0,21 | 0,25 | 0,24 | 0,21 | 0,21 | 0,04 | 0,02 | 0,41 | 0,14 | 0,21 |
| IDC#0586 | 2.8 | 0,56 | 0,28 | 0,00 | 0,29 | 0,14 | 0,07 | 0,20 | 0,10 | 0,03 | 0,00 | 0,39 | 0,18 | 0,19 |
| IDC#0591 | 1.5 | 0,78 | 0,58 | 0,19 | 0,24 | 0,19 | 0,25 | 0,35 | 0,27 | 0,13 | 0,13 | 0,36 | 0,22 | 0,31 |
| IDC#0609 | 2.0 | 0,70 | 0,34 | 0,12 | 0,32 | 0,27 | 0,44 | 0,31 | 0,38 | 0,32 | 0,26 | 0,53 | 0,25 | 0,35 |
| IDC#0629 | NA | NA | NA | NA | NA | NA | NA | NA | NA | NA | NA | NA | NA | NA |
| IDC#0638 | NA | NA | NA | NA | NA | NA | NA | NA | NA | NA | NA | NA | NA | NA |
| IDC#0639 | NA | NA | NA | NA | NA | NA | NA | NA | NA | NA | NA | NA | NA | NA |
| IDC#0654 | NA | NA | NA | NA | NA | NA | NA | NA | NA | NA | NA | NA | NA | NA |
| IDC#0659 | NA | NA | NA | NA | NA | NA | NA | NA | NA | NA | NA | NA | NA | NA |
| IDC#0666 | NA | NA | NA | NA | NA | NA | NA | NA | NA | NA | NA | NA | NA | NA |
| IDC#0669 | NA | NA | NA | NA | NA | NA | NA | NA | NA | NA | NA | NA | NA | NA |
| IDC#0686 | NA | NA | NA | NA | NA | NA | NA | NA | NA | NA | NA | NA | NA | NA |
| IDC#0703 | NA | NA | NA | NA | NA | NA | NA | NA | NA | NA | NA | NA | NA | NA |
| IDC#0706 | 1.8 | 0,42 | 0,32 | 0,18 | 0,39 | 0,42 | 0,17 | 0,18 | 0,25 | 0,27 | 0,23 | 0,44 | 0,13 | 0,28 |
| IDC#0707 | NA | NA | NA | NA | NA | NA | NA | NA | NA | NA | NA | NA | NA | NA |
| IDC#0768 | 1.7 | 0,83 | 0,07 | 0,22 | 0,10 | 0,53 | 0,55 | 0,62 | 0,56 | 0,67 | 0,25 | 0,68 | 0,04 | 0,43 |
| IDC#0782 | 2.2 | 0,56 | 0,31 | 0,10 | 0,21 | 0,18 | 0,07 | 0,44 | 0,48 | 0,30 | 0,04 | 0,46 | 0,18 | 0,28 |
| IDC#0786 | 1.6 | 0,43 | 0,64 | 0,24 | 0,42 | 0,00 | 0,12 | 0,59 | 0,19 | 0,01 | 0,07 | 0,50 | 0,39 | 0,30 |
| IDC#0792 | 2.1 | 0,46 | 0,09 | 0,12 | 0,12 | 0,23 | 0,15 | 0,17 | 0,04 | 0,06 | 0,12 | 0,38 | 0,02 | 0,16 |
| IDC#0796 | NA | NA | NA | NA | NA | NA | NA | NA | NA | NA | NA | NA | NA | NA |
| IDE#0057 | NA | NA | NA | NA | NA | NA | NA | NA | NA | NA | NA | NA | NA | NA |
| IDE#0294 | NA | NA | NA | NA | NA | NA | NA | NA | NA | NA | NA | NA | NA | NA |
| IDF#0033 | NA | NA | NA | NA | NA | NA | NA | NA | NA | NA | NA | NA | NA | NA |

**AUC**

| AUC |
|---|
| >0.5 |
| 0.4-0.5 |
| 0.3-0.39 |
| 0.15-0.29 |
| <0.2 |

**Supplementary Table 5**. Anti-BG505$_{SOSIP.664}$ plasma IgG repertoire of individual IDC561

| | Relative Abundance, CDRH3 (%) | | | |
|---|---|---|---|---|
| Clone | 1st apheresis | 2nd apheresis | V gene | Mutation rate (%) |
| 167 | 15.14 | 11.27 | IGHV1-69 | 11.4 |
| 1942 | 9.27 | 0.19 | IGHV1-18 | 11.7 |
| 4872 | 8.94 | 15.06 | IGHV1-69 | 11.0 |
| 10012 | 7.87 | 2.35 | IGHV2-5 | 14.9 |
| 178 | 7.03 | 9.95 | IGHV3-23 | 11.4 |
| 502 | 6.61 | 0 | IGHV2-5 | 10.0 |
| 3246 | 6.21 | 2.85 | IGHV2-5 | 10.8 |
| 894 | 5.74 | 0 | IGHV1-69 | 7.8 |
| Clone 4 | 5.24 | 0 | IGHV1-46 | 26.2 |
| 2991 | 3.02 | 0.10 | IGHV1-69 | 27.5 |
| 5170 | 2.34 | 0 | IGHV4-34 | 4.7 |
| 6300 | 1.89 | 0 | IGHV3-23 | 3.5 |
| 81 | 1.87 | 0.03 | IGHV1-18 | 16.5 |
| 4632 | 1.86 | 3.94 | IGHV3-30 | 14.0 |
| 585 | 1.82 | 0 | IGHV3-15 | 8.1 |
| 2466 | 1.32 | 0.01 | IGHV4-39 | 8.8 |
| 4941 | 1.26 | 0 | IGHV4-34 | 14.0 |
| 14955 | 0.22 | 16.71 | IGHV1-69 | 13.0 |
| 2671 | 0 | 17.29 | IGHV3-9 | 11.6 |
| 5097 | 0 | 2.30 | IGHV1-8 | 11.8 |
| 9574 | 0 | 0.86 | IGHV1-69 | 7.3 |
| 531 | 0 | 0.85 | IGHV4-31 | 12.5 |
| 4733 | 1.04 | 6.84 | IGHV1-69 | 10.2 |
| 174 | 1.01 | 0 | IGHV4-4 | 11.6 |
| 2099 | 0.91 | 0 | IGHV4-61 | 8.2 |
| 6518 | 0.78 | 0 | IGHV3-15 | 5.9 |
| 5518 | 0.77 | 0 | IGHV3-30 | 9.5 |
| 2388 | 0.61 | 0 | IGHV1-2 | 13.2 |
| 921 | 0.56 | 0 | IGHV4-31 | 8.8 |
| 4343 | 0.55 | 0 | IGHV4-31 | 13.1 |
| 5895 | 0.49 | 0 | IGHV1-69 | 14.7 |
| 3880 | 0.49 | 0 | IGHV3-23 | 8.4 |
| 656 | 0.43 | 0 | IGHV4-34 | 7.8 |
| 1147 | 0.41 | 0 | IGHV4-34 | 24.3 |
| 1802 | 0.39 | 1.29 | IGHV4-30-2 | 15.5 |
| 4575 | 0.37 | 0.10 | IGHV3-49 | 0.1 |
| 5430 | 0.36 | 0 | IGHV3-21 | 20.1 |
| 1580 | 0.33 | 0 | IGHV3-30 | 19.2 |
| 2873 | 0.30 | 0 | IGHV4-59 | 21.3 |
| 7593 | 0.24 | 0 | IGHV2-5 | 3.5 |
| 375 | 0.23 | 0 | IGHV1-8 | 9.3 |
