# Supplementary material for: Dynamics and durability of HIV-1 neutralization are determined by viral replication
Source: Nat Med. 2023 Nov 13;29(11):2763–74. doi: 10.1038/s41591-023-02582-3 (PMC10667105; doi:10.1038/s41591-023-02582-3)
Supplement: Supplementary file 2 — Reporting Summary [file 41591_2023_2582_MOESM2_ESM.pdf]

nature portfolio

Corresponding author(s): Professor Florian Klein

Last updated by author(s): Aug 29, 2023

Reporting Summary

Nature Portfolio wishes to improve the reproducibility of the work that we publish. This form provides structure for consistency and transparency in reporting. For further information on Nature Portfolio policies, see our Editorial Policies and the Editorial Policy Checklist.

Statistics

For all statistical analyses, confirm that the following items are present in the figure legend, table legend, main text, or Methods section.

n/a	Confirmed	
☐	☒	The exact sample size (*n*) for each experimental group/condition, given as a discrete number and unit of measurement
☐	☒	A statement on whether measurements were taken from distinct samples or whether the same sample was measured repeatedly
☐	☒	The statistical test(s) used AND whether they are one- or two-sided<br>*Only common tests should be described solely by name; describe more complex techniques in the Methods section.*
☐	☒	A description of all covariates tested
☐	☒	A description of any assumptions or corrections, such as tests of normality and adjustment for multiple comparisons
☐	☒	A full description of the statistical parameters including central tendency (e.g. means) or other basic estimates (e.g. regression coefficient) AND variation (e.g. standard deviation) or associated estimates of uncertainty (e.g. confidence intervals)
☐	☒	For null hypothesis testing, the test statistic (e.g. *F*, *t*, *r*) with confidence intervals, effect sizes, degrees of freedom and *P* value noted<br>*Give P values as exact values whenever suitable.*
☒	☐	For Bayesian analysis, information on the choice of priors and Markov chain Monte Carlo settings
☒	☐	For hierarchical and complex designs, identification of the appropriate level for tests and full reporting of outcomes
☐	☒	Estimates of effect sizes (e.g. Cohen's *d*, Pearson's *r*), indicating how they were calculated

Our web collection on statistics for biologists contains articles on many of the points above.

Software and code

Policy information about availability of computer code

Data collection	Flow cytometry data were gained by FACS Aria Fusion with BD FACSDiva software (Version 8.0). ELISA plates were measured by Tecan's Sunrise absorbance microplate reader and associated software. To measure neutralization activity, luminescence was measured using a BertholdTech TriStar2S and associated software. B cell receptor repertoire sequence data were generated by an unbiased template-switch-based NGS approach (Ehrhardt et al, Nature Medicine 2019). Antibody sequence data were gained by PCR sequencing at GATC or Eurofins genomics. For some cohorts, clinical data was documented in an electronic case report form (eCRF) using the online cohort platform ClinicalSurveys.net, which is hosted by QuestBack, Oslo, Norway on servers of UHC, Cologne, Germany, as part of a software-as-a-service agreement.

Data analysis

Analyses and quantifications were performed with FlowJo10, Microsoft Excel for Mac (v14.7.3), Geneious Prime software (v.2020.2.4). Further we used Stata Statistical Software: Release 17, IBM SPSS Statistics Version 23 or Graphpad Prism Version 9.3.1 for statistical analyses.
Raw read pre-processing of B-cell NGS data was performed with an in-house pipeline primarily based on self-written Python scripts, IgBLAST, Clustal Omega, and the pRESTO toolkit. Raw IGHV and immunoglobulin light chain variable (IGLV) sequences derived from NGS and single B cell sequencing data were annotated to human V, D, and J germline reference sequences using MiXCR software. The neutralization AUC was computed as the area under the neutralization titration curve with the R package flux (version 0.2.1.).
To quantify the cluster-specific decline in neutralizing serum IgG activity over time, linear mixed effect models (R-function lme4::lmer) with random slope and patient-specific intercept were applied to model the mean AUC as a function of the number of days passed since the baseline visit (available at: https://www.jstatsoft.org/article/view/v067i01). Confidence bands were computed using R-function ggeffects::ggpredict (available at: https://zenodo.org/record/1301148#.ZAHRHi9XZpQ). For sequence alignment, we used ClustalW (Geneious R10; cost matrix: IUB; gap open cost: 15; gap extend cost: 6.66) and the maximum-likelihood phylogenetic tree was calculated using PhyML with 1,000 bootstrap replicates (Guindon et al., 2010) (substitution model: general time reversible [GTR]; Geneious R10). The best-scoring tree was then rooted to IGHV1-46*01.

For manuscripts utilizing custom algorithms or software that are central to the research but not yet described in published literature, software must be made available to editors and reviewers. We strongly encourage code deposition in a community repository (e.g. GitHub). See the Nature Portfolio guidelines for submitting code & software for further information.

Data

Policy information about availability of data

All manuscripts must include a data availability statement. This statement should provide the following information, where applicable:

- Accession codes, unique identifiers, or web links for publicly available datasets
- A description of any restrictions on data availability
- For clinical datasets or third party data, please ensure that the statement adheres to our policy

Aggregated clinical data are available upon request to the corresponding author (F.K.) provided that there is no reasonable risk of de-anonymizing study participants. Individual patient data cannot be shared due to privacy restrictions. The sequence data of the NGS-based unbiased B cell repertoire analysis of IDC561 will be available upon request and after completion of a Data Transfer Agreement between the University Hospital Cologne and the requesting institution/researcher (for requests contact: florian.klein@uk-koeln.de). Nucleotide sequences of all generated antibodies were deposited at GenBank under the accession numbers OR498214–OR498255.

Human research participants

Policy information about studies involving human research participants and Sex and Gender in Research.

Reporting on sex and gender

For our study, we collected data about the sex of the patients. Data on gender was not collected. Thus, we use the term "sex" throughout the final version of the manuscript only. In our study, we included 737 female and 1542 male patients. For 75 patients no information on their sex was available. Sex/gender was not considered in the design of the bio sample collection protocol and samples were collected irrespective of sex and/or gender.

Population characteristics

The population characteristics at study inclusion of the specific cohorts from Germany, Nepal, Tanzania and Cameroon are described are as following: Male sex: Germany 86%, Tanzania 42%, Nepal 57%, Cameroon 26%; Median age: Germany 48 years, Tanzania 35 years, Nepal 38 years, Cameroon 38 years; Median time since HIV-1 diagnosis: Germany 8.3 years, Tanzania 0 years, Nepal 3.6 years, Cameroon 2.5 years; Median CD4 counts: Germany 622/µl, Tanzania 240/µl, Nepal 532/µl, 496/µl); Time on ART: Germany 5.7 years, Tanzania 0.15 years, Nepal 3.64 years, Cameroon 2.5 years.

Recruitment

Patients were recruited at private practices and/or hospitals in Germany (Cologne, Essen, and Frankfurt), Cameroon (Yaoundé), Nepal (Kathmandu), and Tanzania (Mbeya). Patients were asked to participate in the study during their regular visit at the specitic clinic/hospital. Patients were enrolled in our study without any prior selection, and there was no self-selection bias present because the determination of a patient's eligibility for the study was made by the treating physician. Nevertheless, the choice of eligible patients made by the treating physician could potentially introduce bias in our patient selection. Although we acknowledge this potential bias in our recruitment approach, we have a strong conviction that it did not impact the outcomes of our study.

Ethics oversight

Blood and leukapheresis samples were obtained under protocols approved by the Institutional Review Board (IRB) of the University of Cologne (protocols 13-364 and 16-054) and the local IRBs and all participants provided written informed consent.

Note that full information on the approval of the study protocol must also be provided in the manuscript.

Field-specific reporting

Please select the one below that is the best fit for your research. If you are not sure, read the appropriate sections before making your selection.

☒ Life sciences ☐ Behavioural & social sciences ☐ Ecological, evolutionary & environmental sciences

For a reference copy of the document with all sections, see nature.com/documents/nr-reporting-summary-flat.pdf

Life sciences study design

All studies must disclose on these points even when the disclosure is negative.

Sample size	No sample size determination was performed. The sample size of over 2300 HIV-infected patients was chosen to identify a significant number of individuals with broad- and elite neutralizing activity. Previous studies indicate that only around 20-30% of HIV-1 infected individuals develop this activity. The larger sample allowed us to compare patients with varying neutralizing serum levels, determine epitope specificity in top neutralizers, and enable long-term follow-up of patients with broad- and elite neutralizing activity.
Data exclusions	For NGS data, reads were initally filtered for a mean Phred score of 25 and read-lengths of at least 250 bp. Consensus sequences (based on UMIs) were excluded, when the corresponding UMI was found less than 3 times. No other data was excluded from the analyses.
Replication	B cell analysis pipeline validation was performed in bilogical duplicates as described in the methods section.<br>Several replicates of the FACS Analysis of HIV-1 specific B-cells of patient IDC561 were performed on different dates.<br>For our screening, duplicates were performed for 28.362 of 30602 (93%) tests on a different plate using a different well for each patient resulting in a median standard-deviation between all duplicates of 5,2% inhibition (interquartile range: 2,3%-9,2%). For some patients duplicates were not possible due to limited plasma/serum availability. Mean result of the duplicates was used for further analysis.<br>For the assessment of the neutralizing activity over time in the 71 elite-/broad neutralizer, duplicates were performed on a different plate side by side, and the mean of the duplicates was used for further analysis.<br>Neutralization results from monoclonal antibodies were first screened for their neutralizing activity without dublicates. Whenever a neutralization of more than 30% of screened viruses was found, dublicates were performed and IC50 was determined by using the well established Excel Macro from the Montefiori Lab (Montefiori, D. C. in HIV Protocols (eds Vinayaka R. Prasad & Ganjam V. Kalpana) 395-405 (Humana Press, 2009)).<br>All attemps to replicate our results were successfull.
Randomization	In our analysis, we assessed IgG neutralizing activity in study participants by examining their serum/plasma, leading to a study design without interventions and, consequently, no requirement for randomization.
Blinding	In our study, laboratory personnel conducting experiments were aware of the study ID for each participant they handled, but they were blinded to the participant's group allocation and any clinical data. The process of allocating each participant to a specific group and matching them with clinical data was conducted by the lead author after the completion of analyses to ensure an unbiased interpretation of the results.

Reporting for specific materials, systems and methods

We require information from authors about some types of materials, experimental systems and methods used in many studies. Here, indicate whether each material, system or method listed is relevant to your study. If you are not sure if a list item applies to your research, read the appropriate section before selecting a response.

Materials & experimental systems

n/a	Involved in the study	
☐	☒	Antibodies
☐	☒	Eukaryotic cell lines
☒	☐	Palaeontology and archaeology
☒	☐	Animals and other organisms
☐	☒	Clinical data
☒	☐	Dual use research of concern

Methods

n/a	Involved in the study	
☒	☐	ChIP-seq
☐	☒	Flow cytometry
☒	☐	MRI-based neuroimaging

Antibodies

Antibodies used	Anti-human CD20-AF700 (clone 2H7), BD Bioscience, Cat#560631, RRID: AB_2687799<br>Anti-human CD19-AF700 (Clone HIB19), BD Bioscience, Cat#557921, RRID: AB_396942<br>Anti-human IgG-APC (clone G18-145), BD Bioscience, Cat#550931, RRID: AB_2738854<br>Anti-Human IgD-Pe-Cy7 (Clone IA6-2), BD Bioscience, Cat#561314; RRID: AB_10642457<br>Anti-Human IgM-FITC (Clone G20-127), BD Bioscience, Cat#555782; RRID: AB_396117<br>Anti-Human CD27-PE (Clone M-T271), BD Bioscience, Cat#560985; RRID: AB_10563213<br>Peroxidase AffiniPure Goat Anti-Human IgG, Fc[illegible] fragment specific, Jackson ImmunoResearch, Cat#109-035-098; RRID: AB_2337586<br>Monoclonal anti-HIV-1 Env 561_01_18 (1-18) as described in Schommers et al., Cell 2020 (Genbank Accession no heavy chain: MN867953.1, light chain: MN868009.1)<br>Monoclonal anti-HIV-1 Env 561_02_12 (2-12) as described in Schommers et al., Cell 2020 (Genbank Accession no heavy chain: MN867964.1, light chain: MN868018.1)
Validation	All antibodies from<br>BD Biosciences and Jackson ImmunoResearch were checked for human reactivity during quality control.<br>Alexa Fluor® 700 Mouse Anti-Human CD20 (https://www.bdbiosciences.com/content/bdb/paths/generate-tds-document.ca.560631.pdf):<br>Alternate Name: MS4A1; B1; Bp35; LEU-16; S7

Clone: 2H7
Immunogen: Human 6.16c1.3 B cell line
Isotype: Mouse (C57BL/6) IgG2b, κ
Reactivity: QC Testing: Human
Tested in Development: Rhesus, Cynomolgus, Baboon

Anti-human CD19-AF700 (Clone HIB19) (https://www.bdbiosciences.com/content/bdb/paths/generate-tds-document.nz.557921.pdf)
Clone: HIB19
Isotype: Mouse IgG1, κ
Reactivity: QC Testing: Human

Anti-human IgG-APC (clone G18-145) (https://www.bdbiosciences.com/en-eu/products/reagents/flow-cytometry-reagents/research-reagents/single-color-antibodies-ruo/apc-mouse-anti-human-igg.550931)
Reactivity: Human (QC Testing)
Isotyp: Mouse IgG1, κ

Anti-Human IgD-Pe-Cy7 (Clone IA6-2) (https://www.bdbiosciences.com/content/bdb/paths/generate-tds-document.de.561314.pdf)
Alternate Name: IGHD; Ig delta chain C region; Immunoglobulin heavy constant deltaa
Clone: IA6-2
Isotype: Mouse IgG2a, κ
Reactivity: QC Testing: Human

Anti-Human IgM-FITC (Clone G20-127) (https://www.bdbiosciences.com/content/bdb/paths/generate-tds-document.br.555782.pdf)
Clone: G20-127
Isotype: Mouse IgG1, κ
Reactivity: QC Testing: Human

Anti-Human CD27-PE (Clone M-T271)(https://www.bdbiosciences.com/content/bdb/paths/generate-tds-document.us.560985.pdf)
Alternate Name: TNFRSF7; TNF receptor superfamily, member 7; T14; Tp55; S152
Clone: M-T271
Immunogen: Human T-CLL cells
Isotype: Mouse (BALB/c) IgG1, κ
Reactivity: QC Testing: Human
Tested in Development: Rhesus, Cynomolgus, Baboon

Peroxidase AffiniPure Goat Anti-Human IgG, Fc[illegible] fragment specific, (https://www.jacksonimmuno.com/catalog/products/109-035-003)
Specificity: Based on Immunoelectrophoresis and/or ELISA, the antibody reacts with whole molecule human IgG. It also reacts with the light chains of other human immunoglobulins. No antibody was detected against non-immunoglobulin serum proteins. The antibody may cross-react with immunoglobulins from other species.

Moreover, all antibodies are routinely tested by flow cytometry according to the manufacturers protocoll. Anti-HIV-1 Env antibodies were tested against the 12 virus global panel to ensure their activity.

Eukaryotic cell lines

Policy information about cell lines and Sex and Gender in Research

Cell line source(s)	TZM-bl cells (NIH AIDS Reagent Program), HEK293T (American Type Culture Collection), 293-6E cells (National Research Council of Canada), SupT1.CCR5 cells (James Hoxie). The sex of HEK293T, TZM-bl, 293-6E is female, while sex of SUPT1.CCR5 is male.
Authentication	Cell lines were not authenticated.
Mycoplasma contamination	Cell lines were not checked for Mycoplasma contamination.
Commonly misidentified lines (See ICLAC register)	No commonly misidentified cell lines were used in this study.

Clinical data

Policy information about clinical studies
All manuscripts should comply with the ICMJE guidelines for publication of clinical research and a completed CONSORT checklist must be included with all submissions.

Clinical trial registration	Studies have not been registered.
Study protocol	Available on request from the corresponding author.
Data collection	Data collection were performed at each respective site via local data monitoring systems or via the centralized online cohort platform ClinicalSurveys.net (see above). Data were collected and harmonized at the end by personnel of the University Hospital Cologne.
Outcomes	Non-Interventional study. No outcome paramenters were defined.

Flow Cytometry

Plots

Confirm that:

☒ The axis labels state the marker and fluorochrome used (e.g. CD4-FITC).

☒ The axis scales are clearly visible. Include numbers along axes only for bottom left plot of group (a 'group' is an analysis of identical markers).

☒ All plots are contour plots with outliers or pseudocolor plots.

☒ A numerical value for number of cells or percentage (with statistics) is provided.

Methodology

Sample preparation	PBMCs were isolated by standard density gradient centrifugation using Histopaque (Sigma Aldrich) and LeucoSep tubes (Greiner Bio-one). Cells were stored at -150°C in 90% (v/v) FBS (Sigma Aldrich) and 10% (v/v) DMSO (Sigma Aldrich). For NGS of the b cell repertoire, cells were stained with DAPI (Thermo Fisher), CD20-AF 700, IgG-APC, IgD-Pe-Cy7, IgM-FITC, and CD27-PE (all BD Biosciences). 450.000 and 400.000 CD20+IgG+IgM-IgD-CD27- B cells of the 1st and 2nd apheresis of IDC561, respectively, were sorted into FBS (Sigma-Aldrich) using a BD FACSAria Fusion. For the analysis of the naïve B cell repertoire CD20+IgM+IgD+CD27-IgG- cells were sorted.<br>For single b cell sort: Cells were stained with anti-human CD19-AF700 (BD), anti-human IgG-APC (BD), DAPI (Thermo Fisher), and the HIV-1 env baits BG505SOSIP.664-GFP or biotinylated YU2gp140 (labeled with Streptavidin-PE (BD)) for 30 min on ice. Env-reactive CD19+IgG+DAPI- single cells were sorted into 96-well plates containing 4 µl of lysis buffer.
Instrument	FACSAria Fusion (Becton Dickinson)
Software	BD FACSDIVA, FlowJo10
Cell population abundance	Post-sort fractions were not re-analyzed.
Gating strategy	For NGS of B cell repertoire: Gating on lymphocytes, live cells, CD20+ and then IgG+ for antigen experienced or IgD+IgM +CD27-IgG- for naive B cells.<br><br>For single b cell sort: Gating on lymphocytes, live cells, CD19+ and IgG+ and then GFP+ (for BG505SOSIP.664-GFP) or PE+ (for YU2gp140) for HIV-1 reactive cells.

☒ Tick this box to confirm that a figure exemplifying the gating strategy is provided in the Supplementary Information.
